# Comparative efficacy of Traditional Chinese Medicine classical prescriptions for angina pectoris: a network meta-analysis

**DOI:** 10.3389/fphar.2026.1794371

**Published:** 2026-09-28

**Authors:** Hai-Yi Liang, Xin-Yu Ji, Si-Qi Liu, Shu-Han Zhao, Fu-Yi Yang, Bai Du, Shuai Shi

**Affiliations:** 1 Guang’anmen Hospital, China Academy of Chinese Medical Sciences, Beijing, China; 2 Graduate School, Beijing University of Chinese Medicine, Beijing, China; 3 Institute of Basic Research in Clinical Medicine, China Academy of Chinese Medical Sciences, Beijing, China

**Keywords:** angina pectoris, blood lipids, classical prescriptions, coronary heart disease, network meta analysis

## Abstract

**Objective:**

To evaluate the efficacy and safety of classical prescriptions from Shang han Lun (Treatise on Febrile Diseases) and Jin gui Yao lue (Synopsis of the Golden Chamber) combined with conventional biomedicine in the treatment of angina pectoris of coronary heart disease.

**Materials and Methods:**

Eight commonly used Chinese and English databases were searched for relevant clinical studies published before 25 December 2024. Revman 5.4 and Stata14.0 software were used for data analysis.

**Results:**

A total of 131 studies involving 11,292 participants (5,660 in intervention groups and 5,632 in control groups) were included. Most classical prescriptions combined with conventional biomedical therapy showed favorable associations with improvements in angina pectoris efficiency, electrocardiogram (ECG) efficiency, and Traditional Chinese Medicine (TCM) syndrome outcomes compared with conventional treatment alone. Subgroup analyses showed partially different patterns of relative efficacy among prescriptions, with LGZG demonstrating more consistent performance in stable angina pectoris, while other prescriptions showed variable rankings in unstable angina pectoris. For lipid-related and inflammatory outcomes, results were inconsistent across studies, and current evidence remains insufficient to support robust comparative conclusions among classical prescriptions. Regarding safety outcomes, GZFL combined with conventional biomedical therapy showed a relatively favorable safety profile, while other classical prescriptions demonstrated comparable rates of adverse events to conventional therapy.

**Conclusion:**

Classical prescriptions combined with conventional biomedical therapy may be associated with improvements in clinical outcomes of angina pectoris, with an overall acceptable safety profile observed across most intervention. However, due to limitations in study quality, heterogeneity, and indirect comparisons, these findings should be interpreted with caution. Further high-quality, multicenter randomized controlled trials are required to confirm these observations.

**Systematic Review Registration:**

https://www.crd.york.ac.uk/PROSPERO/view/CRD420251029216.

## 1 Introduction

Over the past decades, coronary heart disease (CHD) has remained the leading cause of global morbidity and mortality. According to the latest Global Burden of Disease estimates (2023), cardiovascular diseases affect more than 600 million individuals worldwide and cause approximately 19.2 million deaths annually, with ischemic heart disease remaining the leading subtype (2025). The burden has continued to increase over time, largely driven by population aging and growth despite improvements in age-standardized rates. Risk factor control, lifestyle modification, secondary prevention, and revascularization can reduce the risk of angina and improve prognosis (Knuuti et al., 2020). However, long-term follow-up shows that patients with CHD generally have low compliance with lifestyle management, such as smoking cessation, regular exercise, low-salt and low-fat diet (Kotseva et al., 2019). Anti-ischemic drugs, including nitrates, β-blockers, and calcium channel blockers, as well as percutaneous coronary intervention, are effective in relieving myocardial ischemia-related symptoms. However, symptom relief is not universal, and treatment-related adverse effects remain a concern, including hemodynamic disturbances, bronchospasm, peripheral edema, bleeding risk, and procedure-related complications such as contrast-induced nephropathy and in-stent restenosis. Long-term pharmacotherapy may also be associated with metabolic and organ-related adverse effects, including hepatic dysfunction, new-onset diabetes, and neurocognitive impairment (Frishman, 2003; De Backer, 2011; Münzel et al., 2011; Storey et al., 2011; Byrne et al., 2015; McCullough et al., 2016; Giugliano et al., 2017; Knuuti et al., 2020). Therefore, despite advances in pharmacological and interventional therapies, a proportion of patients with CHD continue to experience persistent symptoms or treatment-related adverse effects. This highlights the need for additional adjunctive therapeutic strategies to further improve symptom management and quality of life.

The works “Shang han Lun (Treatise on Febrile Diseases)” and “Jin gui Yao lue (Synopsis of the Golden Chamber)” authored by Zhongjing Zhang represent foundational works in Traditional Chinese Medicine (TCM) and provide the theoretical basis for classical prescriptions that remain widely used in contemporary clinical practice. In TCM theory, the concepts of “chest obstruction (Xiong Bi)” and “heart pain (Xin Tong)” are considered to correspond to clinical manifestations similar to angina pectoris in modern medicine. Although due to the lack of coronary angiography diagnostic methods in ancient times, differences exist between traditional diagnostic frameworks and biomedical disease definitions, these concepts continue to inform adjunctive clinical practice. In recent years, randomized controlled trials (RCTs) (Chen et al., 2022; Du, 2022; Gu et al., 2022), systematic reviews, and meta-analyses (Liu et al., 2016; Hao, 2022; Chen, 2023; Mao et al., 2023; Wang et al., 2024) have suggested that classical prescriptions such as Gualou Xiebai Banxia Tang (GLXBBX), Gualou Xiebai Baijiu Tang (GLXBBJ), and Zhishi Xiebai Guizhi Tang (ZSXBGZ) may be associated with improvements in angina-related symptoms and quality of life when used in combination with conventional biomedical therapy.

Experimental studies in animal models have provided mechanistic evidence suggesting that classical TCM prescriptions may modulate biological processes related to cardiovascular risk factors, including lipid metabolism and inflammatory responses. Xiao Chaihu Tang (XCH) has been reported to regulate lipid metabolism and inflammation-related pathways including the downregulation of FASN, ACACA, IL-6, and NF-κB expression (Gao et al., 2020). Ling Gui Zhu Gan Tang (LGZG) may attenuate hepatic lipid accumulation through modulation of STING-mediated inflammatory signaling in macrophages (Ju et al., 2018). The combination of Guizhi Fuling Wan (GZFL) and Xiao Xian Xiong Tang (XXX) has been shown to enhance the expression of endothelial nitric oxide synthase and elevate nitric oxide concentrations in the myocardial tissue, thereby conferring a protective effect on the vascular endothelium (Zhu et al., 2018). Zhigancao Tang (ZGC) has been reported to influence circulating cardiovascular-related biomarkers such as apolipoprotein B, endothelin-1, NF-κB, and homocysteine in patients with CHD (Fan et al., 2021). In addition, Renshen Tang (RS) may regulate lipid metabolism–related signaling pathways, including the TRPV1–LXR/IDOL/LDLR axis, potentially contributing to reduced plaque formation in atherosclerosis models (Yuan et al., 2025).

Currently, there is a lack of high-quality evidence directly comparing different classical TCM prescriptions for angina pectoris, with most available RCTs exhibiting substantial heterogeneity in study design, intervention regimens, comparator types, and outcome measures. Against this backdrop, we performed a network meta-analysis of RCTs to compare classical TCM prescriptions used as adjunctive therapies for angina pectoris, aiming to provide a structured summary of the available evidence to support comparative evaluation and evidence-informed research.

## 2 Materials and methods

This study was conducted in accordance with the Preferred Reporting Items for Systematic Reviews and Meta-Analyses (PRISMA) guidelines and was prospectively registered in PROSPERO (Systematic Review Registration: PROSPERO 2025 CRD420251029216).

### 2.1 Inclusion and exclusion criteria

#### 2.1.1 Inclusion criteria

Studies met the following criteria were included: (1) Participants: The study subjects were patients with a clinically confirmed diagnosis of angina pectoris due to coronary heart disease (CHD). The diagnostic criteria for stable angina (SAP) were based on the “2019 ESC Guidelines for the Diagnosis and Management of Chronic Coronary Syndromes” (Knuuti et al., 2020) while those for unstable angina (UA) were based on the “2023 ESC Guidelines for the Management of Acute Coronary Syndromes (Byrne et al., 2023).” (2) Intervention: The observation group received treatment of Traditional Chinese Medicine (TCM) classical prescriptions in addition to the same conventional standard biomedical treatment as the control group. (3) Comparator: The control group received conventional standard biomedical treatment alone. (4) Outcomes: The primary outcomes included the angina pectoris efficacy rate, electrocardiogram (ECG) efficacy rate, and TCM syndrome efficacy rate. These efficacy rates were assessed in accordance with the “General Principles of Clinical Research of New Chinese Medicine (China Food and Drug Administration, 2015)”, “Standard of Diagnosis and Efficacy of Angina pectoris of Traditional Chinese Medicine Heart Disease (Shen and Yao, 2004).” TCM syndrome is assessed using a syndrome scoring scale based on clinical symptoms and signs, with detailed evaluation criteria provided in the Supplementary Material. Secondary outcomes comprised low-density lipoprotein cholesterol (LDL-C), high-density lipoprotein cholesterol (HDL-C), triglycerides (TG), total cholesterol (TC), high-sensitivity C-reactive protein (Hs-CRP), and safety (rate of participants with adverse events). Eligible studies had to report at least one of the above outcomes. 5) Study design: RCTs.

Only studies published in Chinese or English were considered.

#### 2.1.2 Exclusion criteria

Studies were excluded if they met any of the following criteria: (1) inclusion of patients with a confirmed diagnosis of acute myocardial infarction or other severe concomitant diseases; (2) non-randomized study designs, including animal experiments, case reports, conference abstracts, reviews, retrospective studies, meta-analyses, systematic reviews, experience summaries, and theoretical discussions; (3) full text not available; (4) duplicate publications; (5) failure to meet the diagnostic criteria or outcome requirements described above; (6) studies in which any treatment arm included fewer than two participants.

### 2.2 Literature search strategy

The databases used for the search of RCTs studies include PubMed, Embase, Cochrane Library, Web of Science, China National Knowledge Infrastructure (CNKI), China Science Periodical Database (CSPD), China Biology Medicine (CBM), and China Science and Technology Journal Database (VIP).

Chinese search terms mainly include: coronary heart disease, angina pectoris, Gualou Xiebai Baijiu Tang, Gualou Xiebai Banxia Tang, Zhishi Xiebai Guizhi Tang, Fuling Xingren Gancao Tang, Ju Zhi Jiang Tang, Yiyi Fuzi San, Guizhi Shengjiang Zhishi Tang, Wutou Chishizhi Wan (Comprising other dosage forms), Jiu Tong Wan (Comprising other dosage forms), Zhizi Dahuang Tang, Guizhi Fuling Wan (Comprising other dosage forms), Chaihu Guizhi Tang, Chaihu Jia Longgu Muli Tang, Da Chaihu Tang, Xiao Chaihu Tang, Sini Tang, Sini Jia Renshen Tang, Danggui Sini Tang, Guizhi Jia Gui Tang, Xiao Xian Xiong Tang, Zhizi Chi Tang, Banxia Xie Xin Tang, Ling Gui Zhu Gan Tang, Renshen Tang, Zhigancao Tang. English search terms mainly include: angina pectoris, Traditional Chinese Medicine, Tang, Tang, Wan, San, Jian, Yin. The period of literature search is from the establishment of the database to 25 December 2024.

### 2.3 Data collection and quality assessment

Two researchers independently screened the literature in strict accordance with the inclusion and exclusion criteria, and the disagreement was decided by the third researcher. EndnoteX9 software and manual screening were used to check and eliminate the imported literature. Microsoft Excel 2019 software was used to extract the main authors, publication year, sample size, intervention measures, baseline characteristics of the subjects, trial protocols, outcome indicators, adverse reactions and other information of the included studies. To enhance transparency and reproducibility of botanical interventions, we systematically extracted and reported the composition of each prescription involved in the included studies. The completed ConPhyMP checklist is provided in the Supplementary Material. Regarding the composition of the classic prescriptions involved, the taxonomic verification report and the preparation method can be found in Supplementary Tables S1 and S2.

Based on the “Risk of Bias Assessment” tool in the Cochrane Systematic Reviewer’s Manual 5.10, the quality of the literature was evaluated. The content included: 1) random allocation method; 2) concealment of allocation method; 3) blinding of the researchers implementing the intervention and the research subjects; 4) blinding of the evaluation of results; 5) completeness of the result data; 6) selective reporting of research results; 7) other risk of bias. The literature was evaluated as “low risk”, “unclear risk,” or “high risk.”

### 2.4 Data synthesis and analysis

Revman Manager 5.4 was used to draw the literature quality evaluation chart. For binary data (angina pectoris efficiency rate, ECG efficiency rate, TCM syndrome efficiency rate, and safety) and continuous data (LDL-C, HDL-C, TG, TC, and Hs-CRP), odds ratio (OR) and standard mean difference (SMD) were used as effect sizes, respectively. Each effect size was expressed by the corresponding 95% confidence interval (CI). Stata14.0 was used to conduct the network meta-analysis. The node size was positively correlated with the sample size of interventions, and the connection thickness between nodes was positively correlated with the number of RCTs comparing the corresponding two interventions. When each intervention forms a closed loop, inconsistency test is needed to evaluate the consistency of direct comparison and indirect comparison results. Calculate the cumulative area under the probability curve of each intervention measure, draw the map of surface under the cumulative results curve (SUCRA), and estimate the overall results of the efficacy of each intervention measure. Draw the comparison-corrected funnel plot to evaluate publication bias. Global I^2^ was used to assess overall heterogeneity. If I^2^ > 50%, large heterogeneity was considered. Sensitivity analysis was used to evaluate the stability of data pooling.

## 3 Results

### 3.1 Baseline characteristics

According to the established literature search strategy, a total of 3,067 articles were retrieved. A total of 131 articles were included after removing duplicates and reading the abstract and full text of the literature to eliminate those that did not meet the inclusion criteria. The PRISMA retrieval process is shown in Figure 1.

**FIGURE 1:**
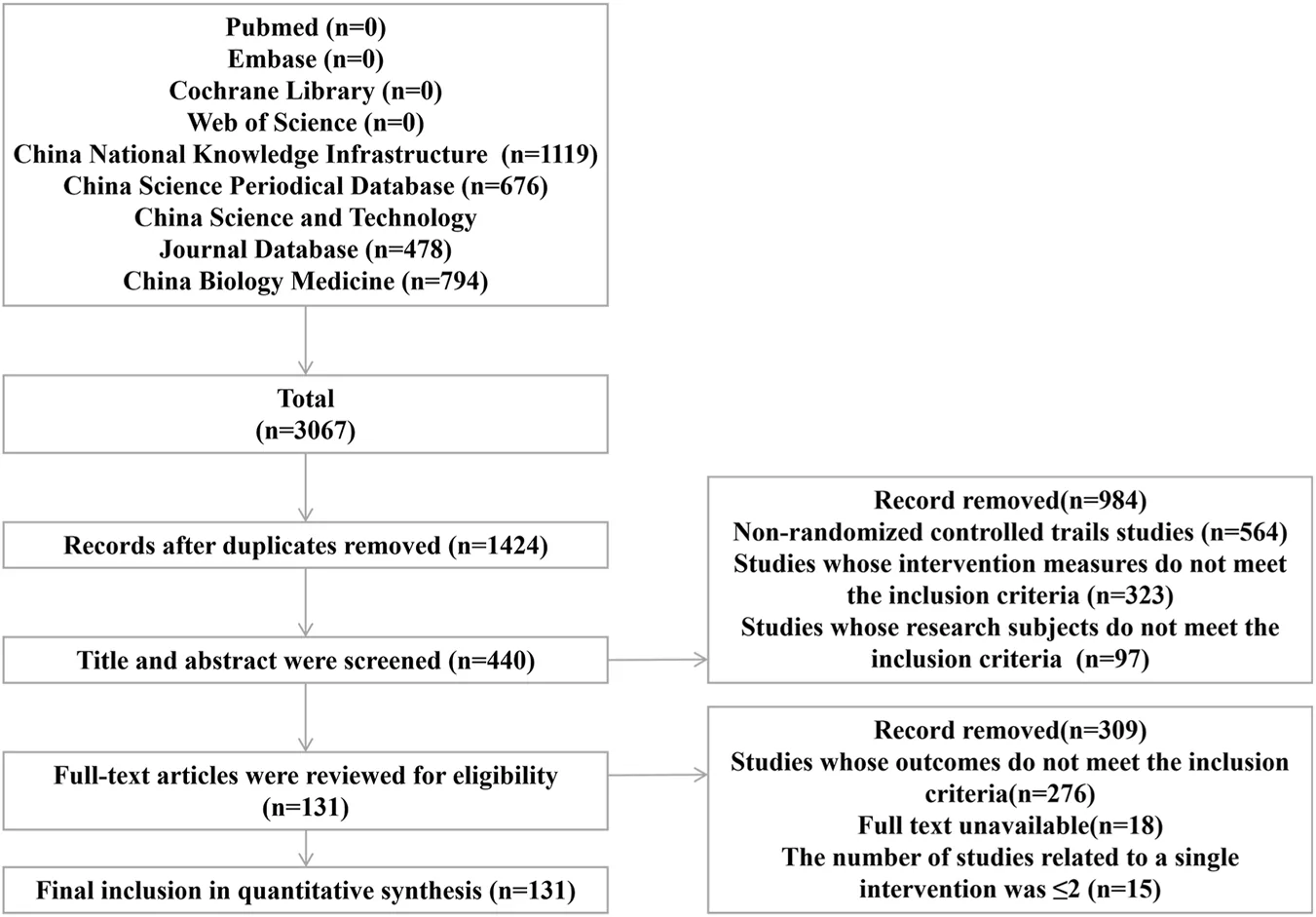
Literature selection process.

A total of 131 randomized controlled trials involving 11,292 participants (5,660 in intervention groups and 5,632 in control groups) were included in this network meta-analysis. All studies were conducted in China and evaluated classical TCM prescriptions as adjunctive therapies in combination with conventional standard biomedical treatment. Control groups primarily received guideline-directed medical therapy for CHD. All interventions were administered orally, and most studies reported short-term treatment courses, although treatment duration varied across trials.

Study populations generally included patients with stable or unstable angina or coronary heart disease with angina symptoms. Reported outcomes mainly focused on clinical symptom improvement, frequency of angina attacks, and electrocardiographic changes, with some studies also reporting lipid metabolism and inflammatory biomarkers.

A total of 11 prescriptions were involved: Danggui Sini Tang (DGSN, 6 studies); Gualou Xiebai Baijiu Tang (GLXBBJ, 6 studies); Gualou Xiebai Banxia Tang (GLXBBX, 51 studies); Guizhi Fuling Wan (GZFL, 9 studies); Ling Gui Zhu Gan Tang (LGZG, 11 studies); Renshen Tang (RS, 5 studies); Sini Tang (SN, 5 studies); Xiao Chaihu Tang (XCH, 2 studies); Xiao Xian Xiong Tang (XXX, 8 studies); Zhigancao Tang (ZGC, 5 studies); Zhishi Xiebai Guizhi Tang (ZSXBGZ, 23 studies). 48 studies reported angina pectoris efficiency rate; 46 studies reported ECG efficiency rate; 48 studies reported TCM syndromes efficiency rate; 56 studies reported the LDL-C level; 46 studies reported the HDL-C level; 61 studies reported the TG level; 61 studies reported the TC level; 18 studies reported the Hs-CRP level; 52 studies reported safety.

Detailed characteristics of the included studies are presented in Supplementary Table S3.

### 3.2 Risk of bias

The risk assessment of 131 RCTs is shown in Figure 2 and Supplementary Figure S1. Among them, 61 studies used the random number table method, 2 studies used the computer table method, 3 studies used Doll’s clinical case random table, 2 studies used the lottery method, 1 study used the coin-tossing method, 1 study used the dice method, 3 studies randomly assigned groups based on the order of admission time, 1 study randomly assigned groups based on odd and even numbers, and 57 studies did not mention the specific randomization method. All literature did not indicate the implementation of allocation concealment. No double-blind method was used in all studies. Seventy-nine studies did not report any adverse events. Therefore, the risk of bias due to selective reporting and other potential sources of bias was rated as unclear. As the included studies faithfully reported the outcomes pre-specified in their protocols, the completeness of outcome data was assessed as low risk of bias.

**FIGURE 2:**
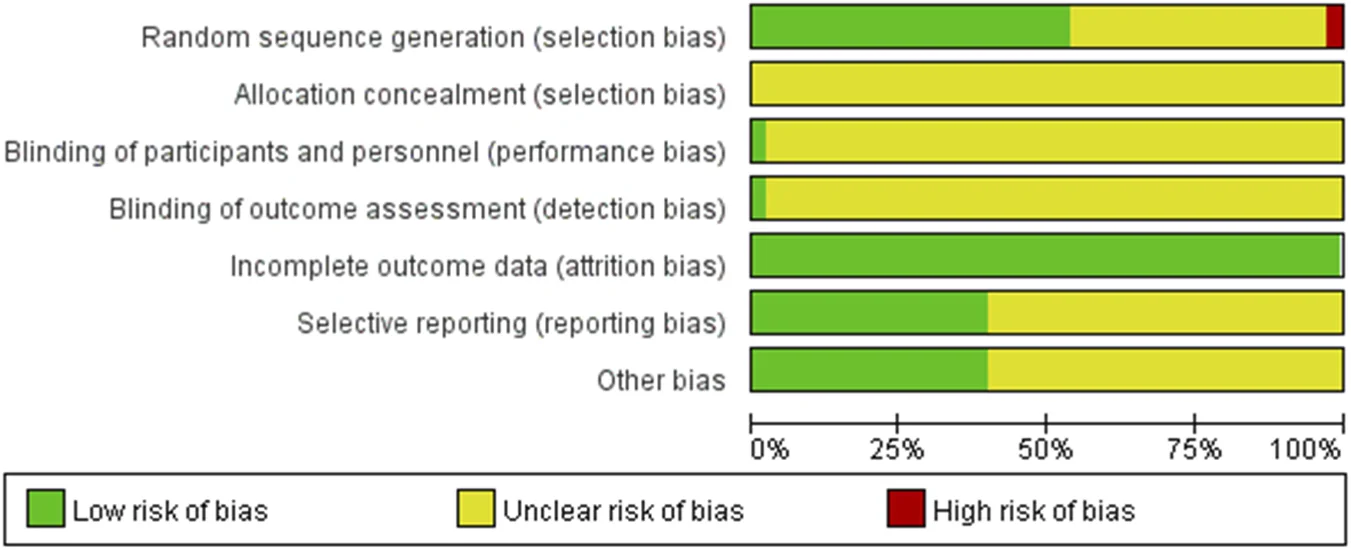
Risk of bias.

### 3.3 Network meta-analysis

Figure 3 shows the network evidence map of each intervention based on the direct comparison relationship. It can be seen that there is no closed loop. There is no direct comparison of each intervention in the original study, as a result, inconsistency test is not applicable. The consistency effect model was used to conduct the network meta-analysis. We further performed subgroup analyses stratified by angina classification (SAP and UA), and the corresponding network evidence plots are presented in Supplementary Figures S2 and S3. SUCRA was applied to describe the probabilistic distribution of treatment effects across interventions for each outcome in an exploratory manner without implying definitive results or hierarchical superiority, given the absence of closed-loop evidence.

**FIGURE 3:**
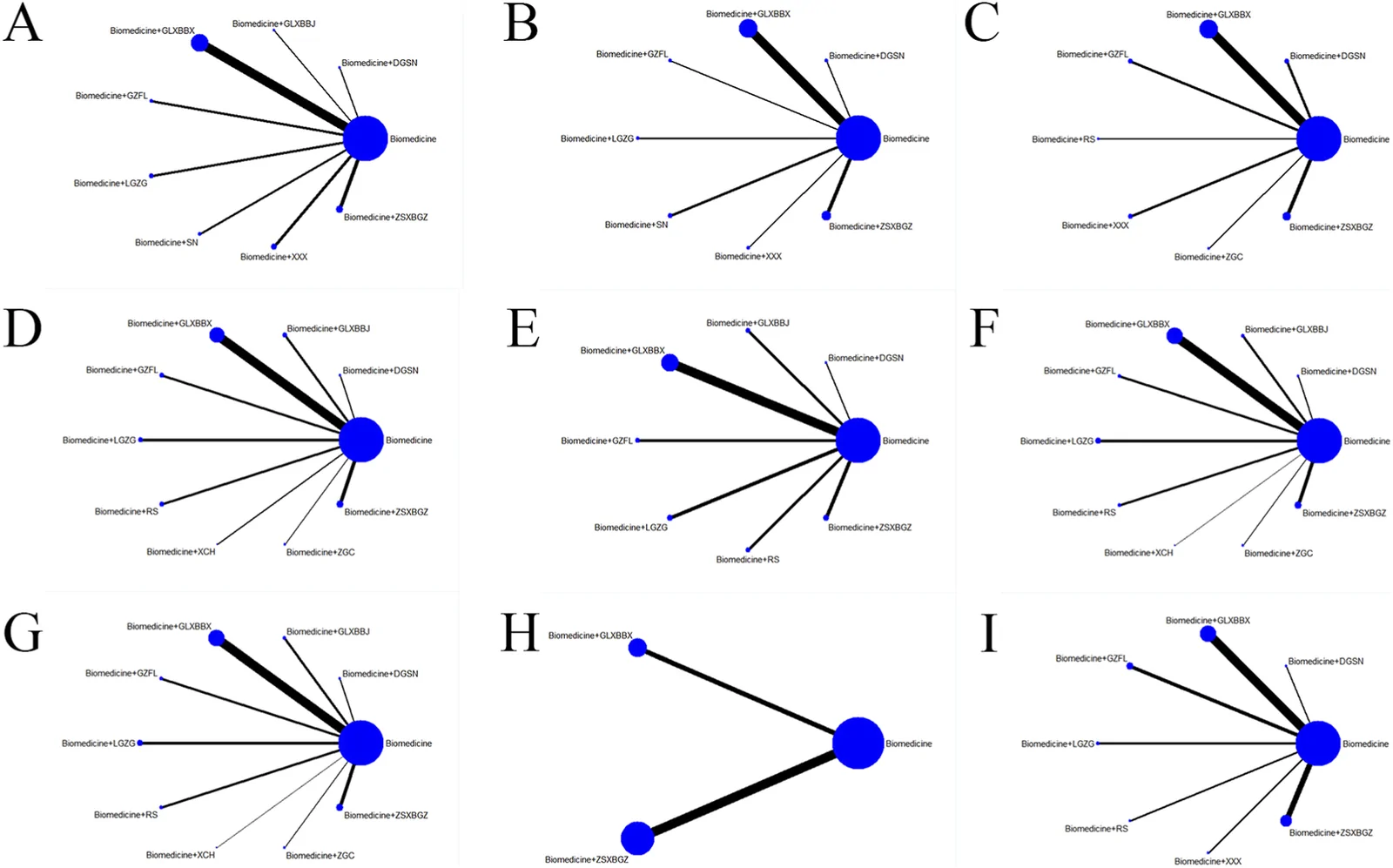
Network evidence plot. **(A)** Angina pectoris efficiency rate. **(B)** ECG efficiency rate. **(C)** TCM syndrome efficiency rate. **(D)** LDL-C. **(E)** HDL-C. **(F)** TG. **(G)** TC. **(H)** Hs-CRP. **(I)** Safety.

#### 3.3.1 Angina pectoris efficiency rate

48 studies reported angina pectoris efficiency rate, involving eight prescriptions (SN, XXX, DGSN, ZSXBGZ, GZFL, GLXBBX, GLXBBJ, LGZG). Compared with Biomedicine, Biomedicine + SN [OR = 0.42, 95% CI = (0.21, 0.82)], Biomedicine + XXX [OR = 0.46, 95% CI = (0.30, 0.71)], Biomedicine + DGSN [OR = 0.29, 95% CI = (0.13, 0.65)], Biomedicine + ZSXBGZ [OR = 0.22, 95% CI = (0.13, 0.39)], Biomedicine + GZFL [OR = 0.28, 95% CI = (0.14, 0.54)], Biomedicine + GLXBBX [OR = 0.29, 95% CI = (0.21, 0.39)], Biomedicine + GLXBBJ [OR = 0.26, 95% CI = (0.11, 0.62)], and Biomedicine + LGZG [OR = 0.20, 95% CI = (0.12, 0.36)] were associated with better angina pectoris efficiency rate, with significant differences, as shown in Table 1. Cumulative probability curve results (Figure 4A): Biomedicine + LGZG (82.1%), Biomedicine + ZSXBGZ (76.0%), Biomedicine + GLXBBJ (64.4%), Biomedicine + GZFL (60.6%), Biomedicine + GLXBBX (56.2%), Biomedicine + DGSN (55.5%), Biomedicine + SN (31.9%), Biomedicine + XXX (23.2) %), Biomedicine (0.1%).

**TABLE 1:** The league table of Angina pectoris efficiency rate.

Intervention	OR (95%CI)*				
	Biomedicine	Biomedicine+DGSN	Biomedicine+GLXBBJ	Biomedicine+GLXBBX	Biomedicine+GZFL
Biomedicine	1				
Biomedicine+DGSN	0.29 (0.13,0.65)	1			
Biomedicine+GLXBBJ	0.26 (0.11,0.62)	0.87 (0.27,2.85)	1		
Biomedicine+GLXBBX	0.29 (0.21,0.39)	0.98 (0.42,2.29)	1.12 (0.44,2.85)	1	
Biomedicine+GZFL	0.28 (0.14,0.54)	0.94 (0.33,2.66)	1.08 (0.36,3.26)	0.96 (0.46,2.03)	1
Biomedicine+LGZG	0.20 (0.12,0.36)	0.70 (0.26,1.84)	0.80 (0.28,2.27)	0.71 (0.37,1.36)	0.74 (0.31,1.79)
Biomedicine+SN	0.42 (0.21,0.82)	1.42 (0.50,4.03)	1.62 (0.53,4.93)	1.45 (0.68,3.07)	1.51 (0.58,3.94)
Biomedicine+XXX	0.46 (0.30,0.71)	1.57 (0.63,3.87)	1.79 (0.67,4.79)	1.60 (0.93,2.75)	1.67 (0.74,3.74)
Biomedicine+ZSSXBGZ	0.22 (0.13,0.39)	0.76 (0.29,2.01)	0.87 (0.31,2.47)	0.78 (0.41,1.48)	0.81 (0.34,1.95)

Intervention	OR (95%CI)*			
	Biomedicine+LGZG	Biomedicine+SN	Biomedicine+XXX	Biomedicine+ZSSXBGZ
Biomedicine+LGZG	1			
Biomedicine+SN	2.04 (0.84,4.95)	1		
Biomedicine+XXX	2.25 (1.10,4.62)	1.11 (0.49,2.49)	1	
Biomedicine+ZSSXBGZ	1.10 (0.49,2.44)	0.54 (0.22,1.30)	0.49 (0.24,0.99)	1

* OR < 1 indicates a favorable effect of the row intervention. A 95% CI crossing 1 suggests a non-significant difference (P > 0.05).

**FIGURE 4:**
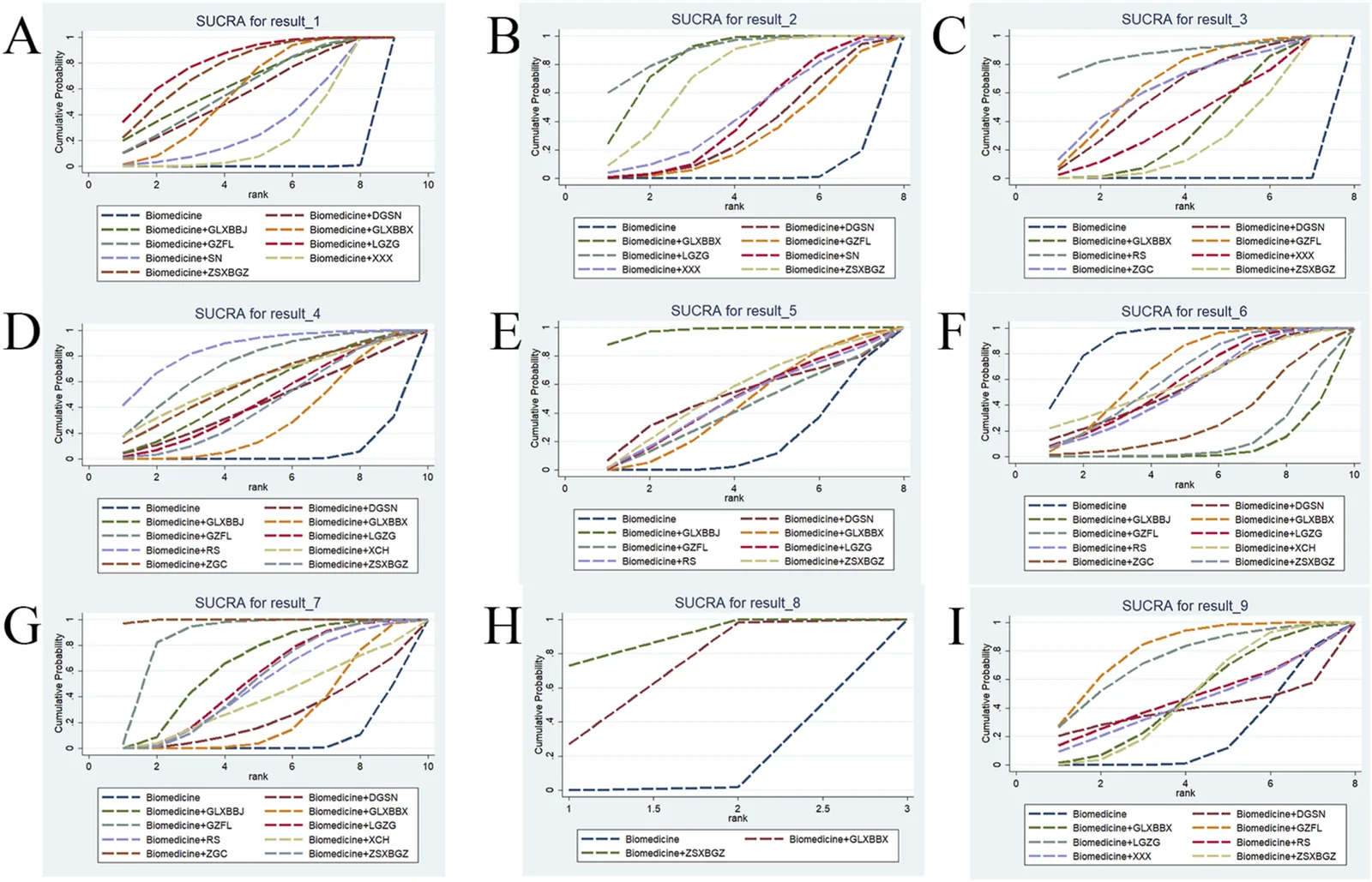
Results from SUCRA. (SUCRA results are presented as exploratory, probability-based summaries in an open network meta-analysis framework). **(A)** Angina pectoris efficiency rate. **(B)** ECG efficiency rate. **(C)** TCM syndrome efficiency rate. **(D)** LDL-C. **(E)** HDL-C. **(F)** TG. **(G)** TC. **(H)** Hs-CRP. **(I)** Safety.

Of the 33 studies reporting angina pectoris efficiency rate in patients with SAP, Biomedicine + DGSN was examined in only one trial and was therefore excluded. A total of 32 SAP-related studies involving seven combination prescriptions (GLXBBJ, GLXBBX, GZFL, LGZG, SN, XXX, ZSXBGZ) were eligible for the subgroup analysis. As presented in Supplementary Table S4A, all combination therapies except Biomedicine + XXX demonstrated significantly superior efficacy relative to Biomedicine alone, with effect estimates ranging from OR = 0.20 for Biomedicine + ZSXBGZ [95% CI (0.09–0.46)] and Biomedicine + LGZG [95% CI (0.12–0.36)] to OR = 0.41 for Biomedicine + SN [95% CI (0.21–0.82)]. Biomedicine + XXX did not differ significantly from Biomedicine alone [OR = 0.57, 95% CI (0.30–1.07)]. Cumulative probability curve results identified Biomedicine + LGZG as the potential intervention which may achieve better efficiency (81.8%).

Of the 15 studies reporting angina pectoris efficiency rate in patients with UA, two combination regimens (Biomedicine + DGSN and Biomedicine + GZFL) were each examined in only one trial and were therefore excluded from the subgroup analysis. The remaining 13 studies, involving three prescriptions (GLXBBX, XXX, ZSXBGZ), were included in the analysis. Based on the league table (Supplementary Table S4B), all three combination therapies significantly outperformed Biomedicine alone: Biomedicine + ZSXBGZ [OR = 0.24, 95% CI = (0.12, 0.52)], Biomedicine + XXX [OR = 0.38, 95% CI = (0.21, 0.70)], and Biomedicine + GLXBBX [OR = 0.24, 95% CI = (0.14, 0.42)]. According to the cumulative probability curve results, Biomedicine + GLXBBX (79.4%) showed a relatively higher value compared with other combinations.

#### 3.3.2 ECG efficiency rate

46 studies reported ECG efficiency rate, involving seven prescriptions (SN, XXX, DGSN, ZSXBGZ, GZFL, GLXBBX, LGZG). As shown in Table 2, in terms of electrocardiogram, Biomedicine + GLXBBX [OR = 0.34, 95% CI = (0.27, 0.45)], Biomedicine + LGZG [OR = 0.30, 95% CI = (0.17, 0.52)], Biomedicine + SN [OR = 0.56, 95% CI = (0.35, 0.90)], and Biomedicine + ZSXBGZ [OR = 0.39, 95% CI = (0.28, 0.56)] were all superior to Biomedicine treatment; there were significant differences. Cumulative probability curve results (Figure 4B): Biomedicine + LGZG (89.6%), Biomedicine + GLXBBX (83.9%), Biomedicine + ZSXBGZ (71.3%), Biomedicine + XXX (45.3%), Biomedicine + SN (42.2%), Biomedicine + DGSN (34.5%), Biomedicine + GZFL (30.1), Biomedicine (3.1%).

**TABLE 2:** The league table of ECG efficiency rate.

Intervention	OR (95%CI)*				
	Biomedicine	Biomedicine+DGSN	Biomedicine+GLXBBX	Biomedicine+GZFL	Biomedicine+LGZG
Biomedicine	1				
Biomedicine+DGSN	0.63 (0.35,1.14)	1			
Biomedicine+GLXBBX	0.34 (0.27,0.45)	0.55 (0.29,1.05)	1		
Biomedicine+GZFL	0.68 (0.36,1.25)	1.07 (0.45,2.53)	1.96 (1.00,3.83)	1	
Biomedicine+LGZG	0.30 (0.17,0.52)	0.48 (0.21,1.07)	0.87 (0.48,1.59)	0.44 (0.19,1.01)	1
Biomedicine+SN	0.56 (0.35,0.90)	0.89 (0.42,1.91)	1.64 (0.96,2.79)	0.83 (0.38,1.81)	1.88 (0.91,3.85)
Biomedicine+XXX	0.55 (0.28,1.07)	0.88 (0.36,2.13)	1.60 (0.79,3.25)	0.82 (0.33,2.02)	1.84 (0.78,4.32)
Biomedicine+ZSXBGZ	0.39 (0.28,0.56)	0.63 (0.31,1.25)	1.15 (0.74,1.76)	0.58 (0.29,1.19)	1.31 (0.69,2.51)

Intervention	OR (95%CI)*		
	Biomedicine+SN	Biomedicine+XXX	Biomedicine+ZSXBGZ
Biomedicine+SN	1		
Biomedicine+XXX	0.98 (0.44,2.20)	1	
Biomedicine+ZSXBGZ	0.70 (0.39,1.26)	0.72 (0.34,1.51)	1

* OR < 1 indicates a favorable effect of the row intervention. A 95% CI crossing 1 suggests a non-significant difference (P > 0.05).

A total of 35 studies in patients with SAP contributed data on ECG efficiency rate. With the exclusion of Biomedicine + DGSN, which was investigated in only one trial, 34 SAP-related studies involving six combination prescriptions (GLXBBX, GZFL, LGZG, SN, XXX, ZSXBGZ) entered the subgroup analysis. As summarized in Supplementary Table S5A, four combination therapies yielded significantly higher ECG efficiency rates than Biomedicine alone: Biomedicine + LGZG [OR = 0.30, 95% CI (0.17–0.52)], Biomedicine + GLXBBX [OR = 0.33, 95% CI (0.25–0.44)], Biomedicine + ZSXBGZ [OR = 0.43, 95% CI (0.28–0.64)], and Biomedicine + SN [OR = 0.57, 95% CI (0.35–0.90)]. By contrast, neither Biomedicine + XXX [OR = 0.57, 95% CI (0.28–1.15)] nor Biomedicine + GZFL [OR = 0.66, 95% CI (0.32–1.38)] differed significantly from Biomedicine alone. Cumulative probability curve results indicated that Biomedicine + LGZG demonstrated a higher probability value (88.2%).

Of the 11 studies reporting ECG efficiency rate in patients with UA, two regimens (Biomedicine + GZFL and Biomedicine + XXX) were each limited to a single trial and therefore excluded. The subgroup analysis of the remaining 9 UA-related studies covered three combination prescriptions: DGSN, GLXBBX, and ZSXBGZ. According to Supplementary Table S5B, both Biomedicine + ZSXBGZ (OR = 0.32, 95% CI 0.17, 0.61) and Biomedicine + GLXBBX (OR = 0.39, 95% CI 0.23, 0.64) demonstrated significantly superior ECG outcomes relative to Biomedicine alone, while the benefit of Biomedicine + DGSN was not statistically significant (OR = 0.58, 95% CI 0.28, 1.20). In the cumulative probability curve results, Biomedicine + ZSXBGZ was evaluated as the intervention with better efficiency (85.3%).

#### 3.3.3 TCM syndromes efficiency rate

48 studies reported TCM syndromes efficiency rate, involving seven prescriptions (RS, XXX, DGSN, ZSXBGZ, GZFL, ZGC, GLXBBX). As shown in Table 3, TCM syndromes efficiency rate of the combination of classical prescriptions and Biomedicine was superior to that of Biomedicine alone. The odds ratios (OR) and 95% confidence intervals (CI) were as follows: Biomedicine + RS [OR = 0.11, 95% CI = (0.02, 0.51)], Biomedicine + XXX [OR = 0.31, 95% CI = (0.16, 0.62)], Biomedicine + DGSN [OR = 0.25, 95% CI = (0.13, 0.46)], Biomedicine + ZSXBGZ [OR = 0.37, 95% CI = (0.25, 0.54)], Biomedicine + GZFL [OR = 0.23, 95% CI = (0.13, 0.39)], Biomedicine + ZGC [OR = 0.23, 95% CI = (0.09, 0.55)], Biomedicine + GLXBBX [OR = 0.33, 95% CI = (0.24, 0.44)]. The differences were statistically significant. Cumulative probability curve results (Figure 4C): Biomedicine + RS (88.2%), Biomedicine + GZFL (69.4%), Biomedicine + ZGC (66.3%), Biomedicine + DGSN (62.4%), Biomedicine + XXX (44.8%), Biomedicine + GLXBBX (39.7%), Biomedicine + ZSXBGZ (29.1%), Biomedicine (0.0%).

**TABLE 3:** The league table of TCM syndrome efficiency rate.

Intervention	OR (95%CI)*				
	Biomedicine	Biomedicine+DGSN	Biomedicine+GLXBBX	Biomedicine+GZFL	Biomedicine+RS
Biomedicine	1				
Biomedicine+DGSN	0.25 (0.13,0.46)	1			
Biomedicine+GLXBBX	0.33 (0.24,0.44)	1.33 (0.67,2.63)	1		
Biomedicine+GZFL	0.23 (0.13,0.39)	0.92 (0.40,2.08)	0.69 (0.37,1.27)	1	
Biomedicine+RS	0.11 (0.02,0.51)	0.44 (0.09,2.31)	0.33 (0.07,1.59)	0.48 (0.10,2.45)	1
Biomedicine+XXX	0.31 (0.16,0.62)	1.27 (0.50,3.20)	0.96 (0.45,2.02)	1.38 (0.58,3.31)	2.86 (0.53,15.31)
Biomedicine+ZGC	0.23 (0.09,0.55)	0.92 (0.31,2.71)	0.69 (0.27,1.77)	1.00 (0.35,2.83)	2.07 (0.35,12.15)
Biomedicine+ZSXBGZ	0.37 (0.25,0.54)	1.50 (0.73,3.10)	1.13 (0.70,1.83)	1.64 (0.85,3.16)	3.38 (0.70,16.38)

Intervention	OR (95%CI)*			
	Biomedicine+XXX	Biomedicine+ZGC	Biomedicine+ZSXBGZ	Biomedicine+XXX
Biomedicine+XXX	1			
Biomedicine+ZGC	0.72 (0.23,2.23)	1		
Biomedicine+ZSXBGZ	1.18 (0.54,2.60)	1.64 (0.62,4.32)	1	

* OR < 1 indicates a favorable effect of the row intervention. A 95% CI crossing 1 suggests a non-significant difference (P > 0.05).

Data on TCM syndrome efficiency rate were available from 28 studies in patients with SAP. Two regimens, Biomedicine + RS and Biomedicine + XXX, were each represented by a single trial and were thus excluded, leaving 26 SAP-related studies covering four combination prescriptions (DGSN, GLXBBX, GZFL, ZSXBGZ) for the subgroup analysis. Supplementary Table S6A shows that every combination therapy significantly outperformed Biomedicine alone: Biomedicine + ZSXBGZ [OR = 0.36, 95% CI (0.21–0.62)], Biomedicine + GZFL [OR = 0.22, 95% CI (0.12–0.41)], Biomedicine + GLXBBX [OR = 0.30, 95% CI (0.21–0.45)], and Biomedicine + DGSN [OR = 0.24, 95% CI (0.10–0.61)]. In the cumulative probability curve results, Biomedicine + GZFL was associated with a relatively higher probability (81.2%).

Of the 20 studies reporting TCM syndrome efficiency rate in patients with UA, Biomedicine + RS was investigated in only one trial and was therefore excluded. The subgroup analysis included 19 UA-related studies and covered five combination prescriptions (DGSN, GLXBBX, XXX, ZGC, ZSXBGZ). As shown in Supplementary Table S6B, all five combination therapies significantly outperformed Biomedicine alone: Biomedicine + ZSXBGZ [OR = 0.38, 95% CI (0.21–0.68)], Biomedicine + ZGC [OR = 0.23, 95% CI (0.09–0.55)], Biomedicine + XXX [OR = 0.28, 95% CI (0.13–0.61)], Biomedicine + GLXBBX [OR = 0.38, 95% CI (0.22–0.65)], and Biomedicine + DGSN [OR = 0.25, 95% CI (0.10–0.59)]. Cumulative probability curve results showed that Biomedicine + ZGC was related to a higher SUCRA value (77.2%).

#### 3.3.4 LDL-C

LDL-C levels were reported in 56 studies involving nine prescriptions (ZSXBGZ, ZGC, XCH, RS, LGZG, GZFL, GLXBBX, GLXBBJ, DGSN). As shown in Table 4, the efficacy of Biomedicine + RS was significantly better than Biomedicine alone [SMD = −10.04, 95% CI = (−16.14, −3.94)]; Biomedicine + GZFL was significantly better than Biomedicine + ZSXBGZ [SMD = −8.13, 95% CI = (−14.07, −2.19)]; Biomedicine + GLXBBX was significantly better than Biomedicine + ZSXBGZ [SMD = −3.09, 95% CI = (−6.12, −0.07)]; and Biomedicine + LGZG was significantly better than Biomedicine + GLXBBX [SMD = −6.95, 95% CI = −13.75, −0.14]]. No significant differences were found among the other pairwise comparisons. Cumulative probability curve results (Figure 4D): Biomedicine + RS (84.9%), Biomedicine + GZFL (73.3%), Biomedicine + XCH (60.6%), Biomedicine + ZGC (59.8%), Biomedicine + GLXBBJ (55.9%), Biomedicine + LGZG (45.0%), Biomedicine + DGSN (44.2%), Biomedicine + ZSXBGZ (41.7%), Biomedicine + GLXBBX (30.1%), Biomedicine (4.5%).

**TABLE 4:** The league table of LDL-C level.

Intervention	SMD (95%CI)*				
	Biomedicine+ZSXBGZ	Biomedicine+ZGC	Biomedicine+XCH	Biomedicine+RS	Biomedicine+LGZG
Biomedicine	0				
Biomedicine+ZSXBGZ	−4.22 (−8.89, 0.44)	0			
Biomedicine+ZGC	−6.44 (−14.06, 1.19)	−2.21 (−11.15, 6.72)	0		
Biomedicine+XCH	−6.78 (−16.10, 2.55)	−2.55 (−12.98, 7.87)	−0.34 (−12.38, 11.70)	0	
Biomedicine+RS	−10.04 (−16.14, −3.94)	−5.82 (−13.49, 1.86)	−3.60 (−13.36, 6.16)	−3.27 (−14.40, 7.87)	0
Biomedicine+LGZG	−4.58 (−9.96, 0.79)	−0.36 (−7.48, 6.76)	1.86 (−7.47, 11.18)	2.19 (−8.57, 12.95)	5.46 (−2.67, 13.59)
Biomedicine+GZFL	−8.13 (−14.07, −2.19)	−3.90 (−11.46, 3.65)	−1.69 (−11.35, 7.98)	−1.35 (−12.40, 9.70)	1.91 (−6.58, 10.41)
Biomedicine+GLXBBX	−3.09 (−6.12, −0.07)	1.13 (−4.43, 6.69)	3.34 (−4.86, 11.54)	3.68 (−6.12, 13.48)	6.95 (0.14, 13.75)
Biomedicine+GLXBBJ	−5.62 (−11.51, 0.27)	−1.40 (−8.91, 6.12)	0.81 (−8.82, 10.45)	1.15 (−9.88, 12.18)	4.42 (−4.06, 12.90)
Biomedicine+DGSN	−4.38 (−11.99, 3.23)	−0.16 (−9.09, 8.77)	2.06 (−8.72, 12.83)	2.39 (−9.64, 14.43)	5.66 (−4.09, 15.41)

Intervention	SMD (95%CI)*				
	Biomedicine+LGZG	Biomedicine+RS	Biomedicine+XCH	Biomedicine+ZGC	Biomedicine+ZSXBGZ
Biomedicine+LGZG	0				
Biomedicine+GZFL	−3.54 (−11.56, 4.47)	0			
Biomedicine+GLXBBX	1.49 (−4.68, 7.66)	5.03 (−1.63, 11.70)	0		
Biomedicine+GLXBBJ	−1.04 (−9.02, 6.94)	2.50 (−5.86, 10.87)	−2.53 (−9.15, 4.09)	0	
Biomedicine+DGSN	0.20 (−9.12, 9.52)	3.74 (−5.91, 13.40)	−1.29 (−9.48, 6.90)	1.24 (−8.39, 10.87)	0

* SMD < 0 indicates a favorable effect of the row intervention. A 95% CI crossing 0 suggests a non-significant difference (P > 0.05).

A total of 43 studies in patients with SAP assessed LDL-C level change, all of which were included in the subgroup analysis covering eight combination prescriptions (DGSN, GLXBBJ, GLXBBX, GZFL, LGZG, RS, XCH, ZSXBGZ). As detailed in Supplementary Table S7A, two interventions achieved significantly greater LDL-C reductions compared with Biomedicine alone: Biomedicine + RS [SMD = −25.35, 95% CI (−34.73 to −15.98)] and Biomedicine + GZFL [SMD = −9.87, 95% CI (−15.97 to −3.77)]. The remaining six regimens showed no statistically significant differences from Biomedicine alone. Cumulative probability curve results indicated that Biomedicine + RS was associated with a probability of 99.9%.

Of the 13 studies reporting LDL-C level change in patients with UA, three combination regimens (Biomedicine + GLXBBJ, Biomedicine + GZFL, Biomedicine + LGZG) were each limited to a single trial and were therefore excluded. The subgroup analysis included 10 UA-related studies and covered four prescriptions: GLXBBX, RS, ZGC, and ZSXBGZ. Based on Supplementary Table S7B, both Biomedicine + ZGC [SMD = −6.42, 95% CI (−11.29 to −1.55)] and Biomedicine + GLXBBX [SMD = −8.47, 95% CI (−14.51 to −2.44)] achieved significantly greater LDL-C reductions compared with Biomedicine alone, whereas the effects of Biomedicine + ZSXBGZ [SMD = −5.11, 95% CI (−11.05 to 0.84)] and Biomedicine + RS [SMD = −1.08, 95% CI (−5.91 to 3.74)] did not reach statistical significance. Cumulative probability curve results suggested that Biomedicine + GLXBBX was associated with a SUCRA value of 86.3% among the interventions, in an exploratory context.

#### 3.3.5 HDL-C

HDL-C levels were reported in 46 studies involving seven prescriptions (RS, DGSN, ZSXBGZ, GZFL, GLXBBX, GLXBBJ, LGZG). As shown in Table 5, the efficacy of Biomedicine + GLXBBJ was significantly better than that of Biomedicine alone [SMD = −8.76, 95% CI = (−13.73, −3.80)], Biomedicine + RS [SMD = −6.92, 95% CI = (−13.77, −0.07)], Biomedicine + LGZG [SMD = −6.94, 95% CI = (−13.51, −0.37)], Biomedicine + GZFL [SMD = −7.47, 95% CI = (−14.31, −0.63)], and Biomedicine + GLXBBX [SMD = −7.26, 95% CI = (−12.85, −1.68)]. No significant differences were detected among the other pairwise comparisons. Cumulative probability curve results (Figure 4E): Biomedicine + GLXBBJ (97.6%), Biomedicine + ZSXBGZ (53.4%), Biomedicine + DGSN (50.3%), Biomedicine + LGZG (47.8%), Biomedicine + RS (47.2%), Biomedicine + GLXBBX (44.7%), Biomedicine + GZFL (40.8%), Biomedicine (18.3%).

**TABLE 5:** The league table of HDL-C level.

Intervention	SMD (95%CI)*				
	Biomedicine	Biomedicine+ZSXBGZ	Biomedicine+RS	Biomedicine+LGZG	Biomedicine+GZFL
Biomedicine	0				
Biomedicine+ZSXBGZ	−2.23 (−6.54, 2.08)	0			
Biomedicine+RS	−1.84 (−6.56, 2.87)	0.39 (−6.00, 6.77)	0		
Biomedicine+LGZG	−1.82 (−6.12, 2.48)	0.41 (−5.68, 6.50)	0.02 (−6.36, 6.40)	0	
Biomedicine+GZFL	−1.29 (−6.00, 3.42)	0.94 (−5.44, 7.32)	0.55 (−6.11, 7.21)	0.53 (−5.85, 6.90)	0
Biomedicine+GLXBBX	−1.50 (−4.06, 1.06)	0.73 (−4.28, 5.74)	0.34 (−5.02, 5.71)	0.32 (−4.68, 5.32)	−0.21 (−5.56, 5.15)
Biomedicine+GLXBBJ	−8.76 (−13.73, −3.80)	−6.53 (−13.10, 0.04)	−6.92 (−13.77, −0.07)	−6.94 (−13.51, −0.37)	−7.47 (−14.31, −0.63)
Biomedicine+DGSN	−2.27 (−9.73, 5.19)	−0.04 (−8.66,8.57)	−0.43 (−9.25, 8.39)	−0.45 (−9.06, 8.16)	−0.98 (−9.80, 7.84)

Intervention	SMD (95%CI)*		
	Biomedicine+GLXBBX	Biomedicine+GLXBBJ	Biomedicine+DGSN
Biomedicine+GLXBBX	0		
Biomedicine+GLXBBJ	−7.26 (−12.85, −1.68)	0	
Biomedicine+DGSN	−0.77 (−8.66, 7.11)	6.49 (−2.47, 15.45)	0

* SMD < 0 indicates a favorable effect of the row intervention. A 95% CI crossing 0 suggests a non-significant difference (P > 0.05).

Data on HDL-C level change were reported in 39 studies of patients with SAP, all of which were included in the subgroup analysis. These studies encompassed seven combination prescriptions (DGSN, GLXBBJ, GLXBBX, GZFL, LGZG, RS, ZSXBGZ). As shown in Supplementary Table S8, only Biomedicine + GLXBBJ significantly elevated HDL-C compared with Biomedicine alone [SMD = −10.92, 95% CI (−17.14 to −4.71)]; none of the other combination therapies demonstrated a statistically significant difference. With a cumulative probability of 97.1%, Biomedicine + GLXBBJ showed a relatively higher benefit for improving HDL-C levels compared with other interventions in this exploratory analysis.

Of the 7 studies reporting HDL-C level change in patients with UA, no intervention was investigated in two or more trials; therefore, a subgroup network meta-analysis could not be conducted.

#### 3.3.6 TG

TG levels were reported in 61 studies involving nine prescriptions (RS, XCH, DGSN, ZSXBGZ, GZFL, ZGC, GLXBBX, GLXBBJ, LGZG). Biomedicine + GLXBBJ [SMD = 12.99, 95% CI = (6.68, 19.31)], Biomedicine + GZFL [SMD = 11.23, 95% CI = (5.00, 17.45)], Biomedicine + ZGC [SMD = 8.25, 95% CI = 0.22, 16.28)] were more effective in improving TG than Biomedicine, as shown in Table 6. Cumulative probability curve results (Figure 4F): Biomedicine + GLXBBJ (92.8%), Biomedicine + GZFL (86.5%), Biomedicine + ZGC (71.7%), Biomedicine + RS (44.3%), Biomedicine + DGSN (43.8%), Biomedicine + LGZG (40.7%), Biomedicine + XCH (40.5%), Biomedicine + ZSXBGZ (37.5%), Biomedicine + GLXBBX (32.4%), Biomedicine (9.8%).

**TABLE 6:** The league table of TG level.

Intervention	SMD (95%CI)*				
	Biomedicine	Biomedicine + DGSN	Biomedicine + GLXBBJ	Biomedicine + GLXBBX	Biomedicine + GZFL
Biomedicine	0	​	​	​	​
Biomedicine + DGSN	3.78 (−4.22, 11.77)	0	​	​	​
Biomedicine + GLXBBJ	12.99 (6.68, 19.31)	9.22 (−0.97, 19.41)	0	​	​
Biomedicine + GLXBBX	2.16 (−0.85, 5.18)	−1.61 (−10.16, 6.93)	−10.83 (−17.83, −3.83)	0	​
Biomedicine + GZFL	11.23 (5.00, 17.45)	7.45 (−2.69, 17.58)	−1.77 (−10.63, 7.10)	9.06 (2.14, 15.98)	0
Biomedicine + LGZG	3.28 (−2.36, 8.92)	−0.50 (−10.29, 9.28)	−9.72 (−18.19, −1.25)	1.11 (−5.28, 7.51)	−7.95 (−16.35, 0.45)
Biomedicine + RS	3.84 (−2.35, 10.03)	0.06 (−10.05, 10.17)	−9.16 (−18.00, −0.31)	1.67 (−5.21, 8.56)	−7.39 (−16.17, 1.39)
Biomedicine + XCH	3.23 (−6.54, 13.00)	−0.55 (−13.18, 12.08)	−9.77 (−21.40, 1.87)	1.06 (−9.16, 11.29)	−8.00 (−19.59, 3.59)
Biomedicine + ZGC	8.25 (0.22, 16.28)	4.47 (−6.86, 15.80)	−4.74 (−14.96, 5.47)	6.09 (−2.49, 14.67)	−2.97 (−13.13, 7.19)
Biomedicine + ZSXBGZ	2.80 (−1.82, 7.41)	−0.98 (−10.21, 8.25)	−10.20 (−18.02, −2.37)	0.63 (−4.88, 6.15)	−8.43 (−16.18, −0.68)

Intervention	SMD (95%CI)*				
	Biomedicine + LGZG	Biomedicine + RS	Biomedicine + XCH	Biomedicine + ZGC	Biomedicine + ZSXBGZ
Biomedicine + LGZG	0	​	​	​	​
Biomedicine + RS	0.56 (−7.81, 8.93)	0	​	​	​
Biomedicine + XCH	−0.05 (−11.33, 11.23)	−0.61 (−12.18, 10.96)	0	​	​
Biomedicine + ZGC	4.98 (−4.84, 14.79)	4.42 (−5.72, 14.55)	5.02 (−7.62, 17.67)	0	​
Biomedicine + ZSXBGZ	−0.48 (−7.76, 6.81)	−1.04 (−8.76, 6.68)	−0.43 (−11.24, 10.38)	−5.45 (−14.71, 3.81)	0

* SMD < 0 indicates a favorable effect of the row intervention. A 95% CI, crossing 0 suggests a non-significant difference (P > 0.05).

Of the 47 studies reporting TG level change in patients with SAP, Biomedicine + ZGC was the only combination represented by a single trial and was therefore excluded. The remaining 46 SAP-related studies, covering eight combination prescriptions (DGSN, GLXBBJ, GLXBBX, GZFL, LGZG, RS, XCH, ZSXBGZ), were included in the subgroup analysis. According to Supplementary Table S9A, two interventions significantly reduced TG levels relative to Biomedicine alone: Biomedicine + GZFL [SMD = −13.81, 95% CI (−19.78 to −7.83)] and Biomedicine + GLXBBJ [SMD = −6.65, 95% CI (−12.56 to −0.75)]. The other six combination regimens showed no statistically significant differences from Biomedicine alone. Cumulative probability curve results identified Biomedicine + GZFL was associated with a relatively higher probability of 97.2%.

Of the 14 studies reporting TG level change in patients with UA, Biomedicine + ZSXBGZ was the only regimen limited to a single trial and was therefore excluded. The subgroup analysis of the remaining 13 UA-related studies covered six combination prescriptions: GLXBBJ, GLXBBX, GZFL, LGZG, RS, and ZGC. As shown in Supplementary Table S9B, three combination therapies achieved significantly greater TG reductions compared with Biomedicine alone: Biomedicine + ZGC [SMD = −4.66, 95% CI (−6.52 to −2.80)], Biomedicine + GLXBBX [SMD = −1.48, 95% CI (−2.72 to −0.24)], and Biomedicine + GLXBBJ [SMD = −43.89, 95% CI (−50.89 to −36.90)]. In contrast, the effects of Biomedicine + RS [SMD = −1.18, 95% CI (−2.61 to 0.26)], Biomedicine + LGZG [SMD = −2.15, 95% CI (−4.66 to 0.35)], and Biomedicine + GZFL [SMD = −0.57, 95% CI (−3.04 to 1.89)] did not reach statistical significance. Cumulative probability curve results suggested that Biomedicine + GLXBBJ was associated with a relatively higher probability of 100%.

#### 3.3.7 TC

TC levels were reported in 61 studies involving nine prescriptions (RS, XCH, DGSN, ZSXBGZ, GZFL, ZGC, GLXBBX, GLXBBJ, LGZG). Biomedicine + GLXBBJ [SMD = 6.92, 95% CI = (1.71, 12.12)], Biomedicine + GZFL [SMD = 11.59, 95% CI = (6.34, 16.83)], Biomedicine + LGZG [SMD = 5.32, 95% CI = 0.93, 9.71)], Biomedicine + ZGC [SMD = 20.15, 95% CI= (13.02, 27.27)], Biomedicine + ZSXBGZ [SMD = 5.08, 95% CI= (1.17, 8.98)] were more effective in improving TC than Biomedicine, as shown in Table 7. Cumulative probability curve results (Figure 4G): Biomedicine + ZGC (99.7%), Biomedicine + GZFL (86.1%), Biomedicine + GLXBBJ (64.6%), Biomedicine + LGZG (53.5%), Biomedicine + ZSXBGZ (51.6%), Biomedicine + RS (48.7%), Biomedicine + XCH (38.5%), Biomedicine + GLXBBX (25.8%), Biomedicine + DGSN (24.5%), Biomedicine (7.0%).

**TABLE 7:** The league table of TC level.

Intervention	SMD (95%CI)*				
	Biomedicine	Biomedicine+DGSN	Biomedicine+GLXBBJ	Biomedicine+GLXBBX	Biomedicine+GZFL
Biomedicine	0				
Biomedicine+DGSN	1.58 (−5.12, 8.27)	0			
Biomedicine+GLXBBJ	6.92 (1.71, 12.12)	5.34 (−3.14, 13.82)	0		
Biomedicine+GLXBBX	2.13 (−0.34, 4.61)	0.56 (−6.58, 7.70)	−4.78 (−10.55, 0.98)	0	
Biomedicine+GZFL	11.59 (6.34, 16.83)	10.01 (1.50, 18.52)	4.67 (−2.72, 12.06)	9.45 (3.65, 15.25)	0
Biomedicine+LGZG	5.32 (0.93, 9.71)	3.75 (−4.26, 11.75)	−1.60 (−8.41, 5.21)	3.19 (−1.85, 8.23)	−6.27 (−13.11, 0.58)
Biomedicine+RS	4.78 (−0.43, 9.99)	3.21 (−5.28, 11.69)	−2.14 (−9.50, 5.23)	2.65 (−3.12, 8.42)	−6.80 (−14.20, 0.59)
Biomedicine+XCH	3.45 (−4.76, 11.65)	1.87 (−8.72, 12.46)	−3.47 (−13.19, 6.24)	1.31 (−7.26, 9.88)	−8.14 (−17.88, 1.60)
Biomedicine+ZGC	20.15 (13.02, 27.27)	18.57 (8.79, 28.35)	13.23 (4.40, 22.05)	18.01 (10.47, 25.56)	8.56 (−0.29, 17.40)
Biomedicine+ZSXBGZ	5.08 (1.17, 8.98)	3.50 (−4.25, 11.25)	−1.84 (−8.35, 4.67)	2.94 (−1.68, 7.57)	−6.51 (−13.05, 0.03)

Intervention	SMD (95%CI)*				
	Biomedicine+RS	Biomedicine+XCH	Biomedicine+ZGC	Biomedicine+ZSXBGZ	Biomedicine+RS
Biomedicine+LGZG	0				
Biomedicine+RS	−0.54 (−7.36, 6.28)	0			
Biomedicine+XCH	−1.87 (−11.18, 7.43)	−1.34 (−11.05, 8.38)	0		
Biomedicine+ZGC	14.82 (6.45, 23.20)	15.36 (6.53, 24.19)	16.70 (5.83, 27.57)	0	
Biomedicine+ZSXBGZ	−0.24 (−6.12, 5.63)	0.30 (−6.22, 6.81)	1.63 (−7.45, 10.72)	−15.07 (−23.19, −6.94)	0

* SMD < 0 indicates a favorable effect of the row intervention. A 95% CI crossing 0 suggests a non-significant difference (P > 0.05).

All 47 studies reporting TC level change in patients with SAP were included in the subgroup analysis, which covered eight combination prescriptions (DGSN, GLXBBJ, GLXBBX, GZFL, LGZG, RS, XCH, ZSXBGZ). As shown in Supplementary Table S10A, six of the eight combination therapies led to significantly greater reductions in TC compared with Biomedicine alone: Biomedicine + GZFL [SMD = −19.09, 95% CI (−24.64 to −13.54)], Biomedicine + RS [SMD = −9.86, 95% CI (−16.57 to −3.14)], Biomedicine + LGZG [SMD = −6.11, 95% CI (−10.30 to −1.92)], Biomedicine + GLXBBJ [SMD = −5.92, 95% CI (−10.60 to −1.24)], Biomedicine + ZSXBGZ [SMD = −5.13, 95% CI (−8.48 to −1.77)], and Biomedicine + GLXBBX [SMD = −2.14, 95% CI (−4.22 to −0.05)]. The remaining two combinations, Biomedicine + XCH [SMD = −3.44, 95% CI (−10.04 to 3.15)] and Biomedicine + DGSN [SMD = −1.58, 95% CI (−6.96 to 3.81)], did not differ significantly from Biomedicine alone. In the cumulative probability curve results, Biomedicine + GZFL received a higher SUCRA value compared with other interventions (99.7%).

Among trials enrolling patients with UA, TC level change was reported in 14 studies. Two interventions (Biomedicine + GLXBBJ and Biomedicine + ZSXBGZ) were each studied in only 1 trial, thus precluding subgroup analysis. Subgroup analysis was performed on 12 UA-related studies, involving five prescriptions (GLXBBX, GZFL, LGZG, RS, ZGC). As shown in Supplementary Table S10B, compared with Biomedicine alone, Biomedicine + ZGC [SMD = −20.69, 95% CI = (−30.13, −11.25)] was associated with significantly lower TC levels. No significant differences were found for Biomedicine + RS [SMD = −1.46, 95% CI = (−10.54, 7.62)], Biomedicine + LGZG [SMD = −3.31, 95% CI = (−14.43, 7.82)], Biomedicine + GZFL [SMD = −0.65, 95% CI = (−11.77, 10.46)], or Biomedicine + GLXBBX [SMD = −2.05, 95% CI = (−13.18, 9.07)]. Cumulative probability curve results indicated that Biomedicine + ZGC was related to a relatively higher probability of 99.6%.

#### 3.3.8 Hs-CRP

Hs-CRP levels were reported in 18 studies involving two prescriptions (GLXBBX, ZSXBGZ). As shown in Table 8, Biomedicine + GLXBBX was associated with significantly lower Hs-CRP levels than Biomedicine alone [SMD = 2.23, 95% CI = (0.25, 4.21)]. No significant difference was found between Biomedicine + ZSXBGZ and Biomedicine alone [SMD = −0.77, 95% CI = (−3.24, 1.70)]. Cumulative probability curve results (Figure 4H): Biomedicine + ZSXBGZ (86.5%), Biomedicine + GLXBBX (62.6%), Biomedicine (0.9%).

**TABLE 8:** The league table of Hs-CRP level.

Intervention	SMD (95%CI)*		
	Biomedicine	Biomedicine+GLXBBX	Biomedicine+ZSXBGZ
Biomedicine	0		
Biomedicine+GLXBBX	2.23 (0.25, 4.21)	0	
Biomedicine+ZSXBGZ	−0.77 (−3.24, 1.70)	−3.00 (−4.47, −1.53)	0

* SMD < 0 indicates a favorable effect of the row intervention. A 95% CI crossing 0 suggests a non-significant difference (P > 0.05).

Of the 12 studies that reported Hs-CRP level change in patients with SAP, Biomedicine + LGZG was represented by only one trial and was therefore excluded. The subgroup analysis comprised 11 SAP-related studies covering two combination prescriptions (GLXBBX and ZSXBGZ). As shown in Supplementary Table S11, Biomedicine + ZSXBGZ significantly reduced Hs-CRP compared with Biomedicine alone [SMD = −3.54, 95% CI (−5.37 to −1.71)], whereas the effect of Biomedicine + GLXBBX did not reach statistical significance [SMD = −1.60, 95% CI (−3.59 to 0.39)]. Cumulative probability curve results showed that Biomedicine + ZSXBGZ was associated with a probability of 96.0%.

Among the six studies reporting Hs-CRP level change in patients with UA, only two interventions were represented: Biomedicine + ZSXBGZ and Biomedicine + GLXBBX. However, Biomedicine + GLXBBX was evaluated in only one trial, thus precluding a valid subgroup network meta-analysis.

#### 3.3.9 Adverse events

A total of 52 studies, involving nine distinct prescriptions (GLXBBJ, GLXBBX, GZFL, LGZG, RS, XCH, SN, XXX, and ZSXBGZ), reported data on adverse events. Three combination regimens (Biomedicine plus GLXBBJ, XCH, or SN) were evaluated in only a single trial each and were subsequently excluded as they did not meet the robust statistical requirements for network meta-analysis. Consequently, 49 studies investigating six prescriptions (GLXBBX, GZFL, LGZG, RS, XXX, and ZSXBGZ) were systematically analyzed. As presented in Table 9, Biomedicine alone was associated with a significantly higher risk of adverse events than Biomedicine + GZFL [OR = 2.41, 95% CI = (1.34, 4.39)]; no other statistically significant differences were observed in the remaining pairwise comparisons. According to the cumulative probability curve results (Figure 4I), Biomedicine + GZFL had a higher probability of being the safest regimen compared to the others (81.1%), followed by Biomedicine + LGZG (74.3%), Biomedicine + ZSXBGZ (47.9%), Biomedicine + GLXBBX (47.5%), Biomedicine + RS (46.7%), and Biomedicine + XXX (43.4%).

**TABLE 9:** The league table of adverse events.

Intervention	OR (95%CI)*				
	Biomedicine	Biomedicine+ZSXBGZ	Biomedicine+XXX	Biomedicine+RS	Biomedicine+LGZG
Biomedicine	1				
Biomedicine+ZSXBGZ	1.40 (0.96, 2.05)	1			
Biomedicine+XXX	1.28 (0.32, 5.10)	0.91 (0.22, 3.86)	1		
Biomedicine+RS	1.35 (0.29, 6.30)	0.97 (0.20, 4.71)	1.05 (0.13, 8.41)	1	
Biomedicine+LGZG	2.25 (0.90, 5.58)	1.60 (0.60, 4.31)	1.75 (0.33, 9.21)	1.67 (0.28, 9.97)	1
Biomedicine+GZFL	2.41 (1.34, 4.39)	1.73 (0.85, 3.49)	1.88 (0.42, 8.50)	1.79 (0.34, 9.30)	1.07 (0.36, 3.19)
Biomedicine+GLXBBX	1.39 (0.82, 2.39)	1.00 (0.52, 1.93)	1.09 (0.25, 4.85)	1.03 (0.20, 5.31)	0.62 (0.21, 1.79)

Intervention	OR (95%CI)*			
	Biomedicine+GZFL	Biomedicine+GLXBBX	Biomedicine+DGSN	
Biomedicine	Biomedicine+GZFL	1		
Biomedicine+ZSXBGZ	Biomedicine+GLXBBX	0.58 (0.26, 1.28)	1	
Biomedicine+XXX	Biomedicine+DGSN	0.42 (0.02, 7.32)	0.73 (0.04, 12.4)	1

* OR < 1 indicates that the intervention in the row is associated with a higher rate of adverse reactions compared with the intervention in the column. A 95% CI crossing 1 suggests a non-significant difference (P > 0.05).

Data on adverse events were available from 37 studies in patients with SAP. Three combination regimens (Biomedicine + DGSN, Biomedicine + SN, and Biomedicine + XCH) were each investigated in only one trial and were therefore excluded. The subgroup analysis of the remaining 34 SAP-related studies encompassed four combination prescriptions: GLXBBX, GZFL, LGZG, and ZSXBGZ. Based on Supplementary Table S12A, the only statistically significant finding was a higher risk of adverse events with Biomedicine + GZFL compared with Biomedicine alone [OR = 2.41, 95% CI (1.22–4.81)]; the other three combination therapies did not differ significantly from Biomedicine alone. In the cumulative probability curve results, Biomedicine + GZFL received a relatively higher probability (82.6%).

Among the 15 trials enrolling patients with UA) that reported adverse event rates, two interventions (Biomedicine + DGSN, Biomedicine + GLXBBJ) were investigated in only a single trial each and were therefore excluded from the subgroup analysis. Consequently, the subgroup analysis was restricted to 13 UA-related studies evaluating four specific prescriptions (GLXBBX, RS, XXX, and ZSXBGZ). As detailed in Supplementary Table S12B, none of the combination therapies demonstrated a statistically significant difference compared with Biomedicine alone. Based on the cumulative probability curve results, Biomedicine + ZSXBGZ was associated with a lower probability of adverse events (73%).

### 3.4 Publication bias

Figure 5 shows the comparison adjusted funnel plots of nine outcomes. The comparison adjusted funnel plots for angina pectoris efficiency rate, ECG efficiency rate, TCM syndrome efficiency rate, and safety all indicate that the included studies were symmetrically distributed in the upper part and clustered along the center line, suggesting a relatively low risk of publication bias. The comparison adjusted funnel plots for LDL-C, HDL-C, TG, TC and Hs-CRP showed poor symmetry and had the risk of publication bias, which may be related to the insufficient number of included studies or the small total sample size.

**FIGURE 5:**
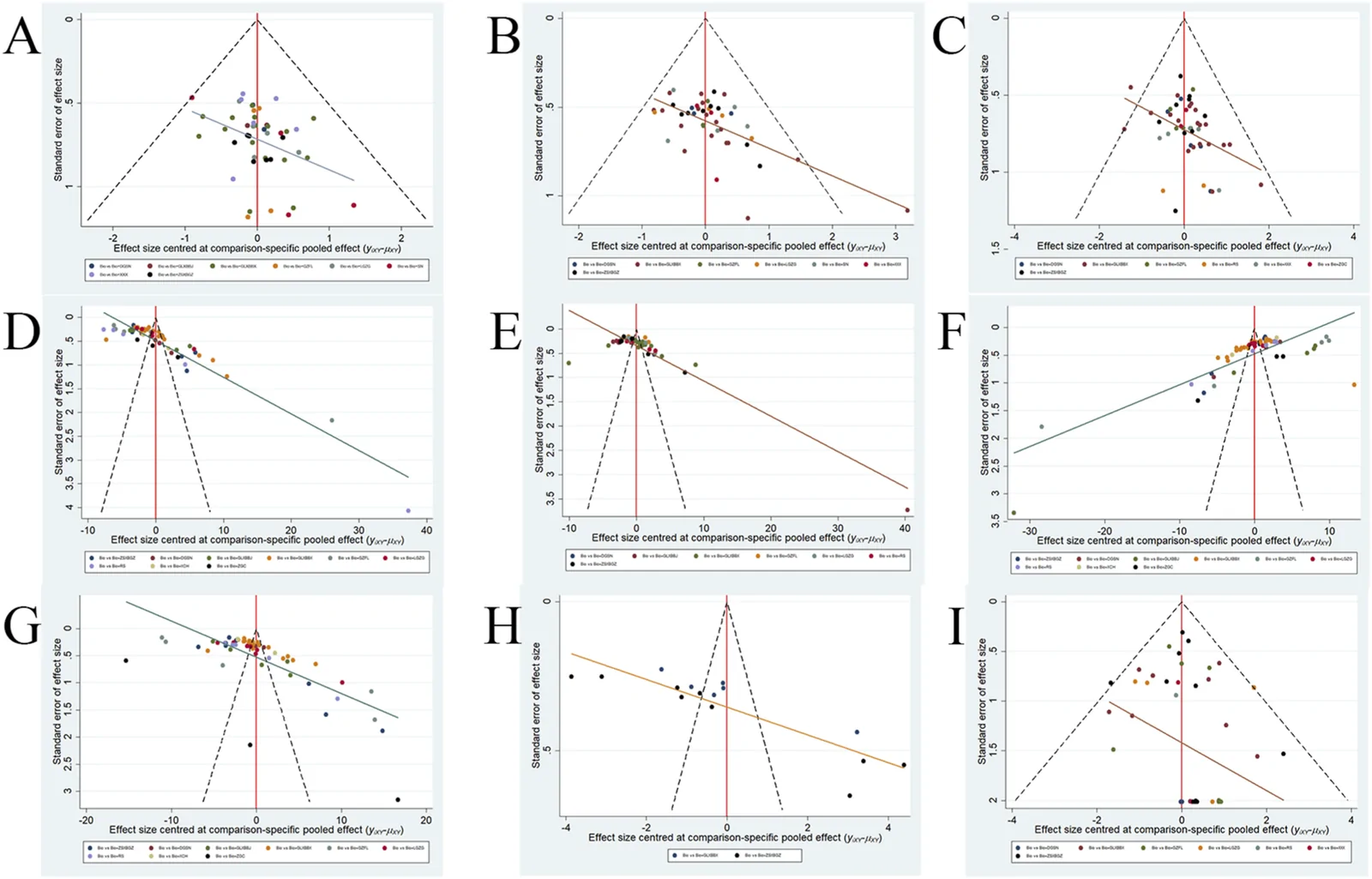
Funnel plots. **(A)** Angina pectoris efficiency rate. **(B)** ECG efficiency rate. **(C)** TCM syndrome efficiency rate. **(D)** LDL-C. **(E)** HDL-C. **(F)** TG. **(G)** TC. **(H)** Hs-CRP. **(I)** Safety.

### 3.5 Network inconsistency and heterogeneity

No closed loop was formed in this network meta-analysis, so the inconsistency test was not applicable. The global I^2^ of angina pectoris efficiency rate, ECG efficiency rate, TCM syndrome efficiency rate, and safety were 0%, 0%, 0%, and 25%, respectively. The global I^2^ for LDL-C, HDL-C, TG, TC and Hs-CRP was 98%, 97%, 98%, 98% and 97%, respectively. As shown in Supplementary Figures S24–S28, the sensitivity analysis showed that excluding any of the studies did not change the expected confidence intervals.

## 4 Discussion

In recent years, accumulating evidence from meta-analyses has suggested that TCM interventions may provide adjunctive benefits in the management of coronary heart disease. Previous studies have reported that the use of commercial Chinese polyherbal preparations (CCPP) in combination with conventional biomedical therapy, may improve angina attack frequency and duration in patients with CHD complicated by diabetes mellitus (Han et al., 2026). In addition, TCM interventions (including botanic decoctions, injectable preparations, and proprietary formulations) have been associated with improved cardiac function following percutaneous coronary intervention (PCI), with some studies indicating reductions in in-stent restenosis and non-fatal myocardial infarction rates (Wang et al., 2026). Currently no network meta-analysis has specifically evaluated the comparative efficacy of classical TCM prescriptions as adjunctive therapy for angina pectoris in CHD. The findings in this study suggest that most classical prescriptions combined with conventional biomedical treatment were associated with improvements in angina pectoris efficiency rate, ECG efficiency rate, TCM syndrome effectiveness rate, and selected metabolic indicators compared with biomedical treatment alone. However, the evidence structure was sparse and star-shaped without closed loops, and substantial heterogeneity was observed across outcomes related to metabolic indicators. Therefore, SUCRA-based rankings should be interpreted cautiously as exploratory rather than confirmatory evidence.

### 4.1 Angina pectoris efficiency and ECG efficiency

Regarding angina pectoris efficiency rate and ECG efficiency, combination with LGZG demonstrated statistically significant superiority over conventional biomedical therapy alone in both outcomes and was consistently associated with higher results in SUCRA values, suggesting a relatively higher probability of being effective adjunctive interventions for overall efficient of angina pectoris symptom and ECG improvement. These findings are consistent with a previous meta-analysis reported by Wei (2024), supporting the robustness of the present results. In addition, a clinical case report have reported that after 2 years of treatment with LGZG combined with conventional biomedical treatment, the degree of coronary artery stenosis decreased significantly, and the degree of right coronary artery stenosis decreased from 90% to <50% (Shi et al., 2022). From a TCM perspective, the Synopsis of the Golden Chamber states that the pathogenesis of “chest obstruction (xiong bi)”includes the involvement of “phlegm retention (tan yin)” as an important etiological factor. LGZG is commonly used to address pathophysiological patterns such as phlegm retention (tan yin) and qi deficiency (yang xu), which are frequently associated with chest discomfort and impaired circulation. Consistently, data mining and prescription pattern analyses have shown that LGZG is most strongly associated with symptoms such as dizziness, chest tightness, and palpitations, which overlap with clinical manifestations of angina pectoris (Zhao, 2025) Preclinical pharmacological studies suggest that metabolites from the major botanical drugs in LGZG (including *Smilax glabra* Roxb [Smilacaceae], *Neolitsea cassia* (L.). Kosterm. [Lauraceae], *Atractylodes macrocephala* Koidz. [Asteraceae], *Glycyrrhiza glabra* L. [Fabaceae]) may exert cardioprotective effects by modulating oxidative stress and inflammatory pathways, including downregulation of LOX-1 and MMP-2, and inhibition of p38/MAPK and NF-κB signaling pathways, thereby potentially attenuating oxidized -LDL-induced vascular smooth muscle cell proliferation and inflammatory responses (Li et al., 2018).

In the subgroup analyses of angina pectoris efficiency, findings in patients with SAP were generally consistent with the overall analysis, in which combination with LGZG remained significantly associated with improved outcomes compared with conventional biomedical therapy and showed a relatively higher Cumulative probability curve result. By contrast, in the subgroup of patients with UA, GLXBBX appeared to show a favorable effect when used as an adjunct to conventional biomedical therapy and received a relatively higher SUCRA value. This observation is partially consistent with a previous meta-analysis reported by Qiu and Zhai., which also suggested potential benefits of GLXBBX-based interventions in improving UA-related symptoms and ECG outcomes (Qiu and Zhai, 2018). In addition, a randomized controlled trial involving 117 UA patients reported that GLXBBX combined with ticagrelor may improve cardiac and endothelial function, as indicated by improvements in flow-mediated dilation and left ventricular ejection fraction compared with conventional biomedical therapy (p < 0.05) (Yu et al., 2026). From a TCM perspective, UA is frequently associated with patterns such as phlegm and blood stasis mutual obstruction (34%, supported by a cross-sectional study) (Sun, 2025). Expert consensus on TCM diagnosis and treatment of unstable angina pectoris 2022 have suggested GLXBBX-containing regimens as a potential option for this pattern, although the recommendation remains weak (evidence level C) (Zhang et al., 2022). Pharmacological studies have demonstrated that GLXBBX can upregulate the protein expression of PI3K, mTOR, HIF-1α, and VEGF in myocardial tissue and improve endothelial cell function, which may promote myocardial microvascular angiogenesis and represent one of the mechanisms underlying its anti-myocardial ischemic effects (Wang et al., 2018).

In regard to ECG efficiency, SAP subgroup results remained consistent with the overall analysis, again supporting the exploratory benefits of the combination of LGZG and conventional biomedical treatment. However, in the UA subgroup, ZSXBGZ appeared to demonstrate more favorable effects when used as an adjunct to conventional biomedical therapy, suggesting a potentially context-dependent pattern of efficacy in more acute clinical presentations. Previous meta-analyses have consistently supported these findings. A 2018 meta-analysis by Tang and Cai et al. reported that ZSXBGZ demonstrated benefits in improving clinical efficacy, ECG outcomes, Seattle Angina Questionnaire scores, angina duration, left ventricular ejection fraction, lipid profiles, and safety outcomes in patients with UA (Tang et al., 2018). A 2023 network meta-analysis by Chao and Qiong et al. further indicated that, in patients with CHD with intermingled phlegm and blood stasis syndrome, ZSXBGZ combined with conventional biomedical therapy showed superiority in reducing the duration of angina attacks (Chao et al., 2023). In addition, a 2024 network meta-analysis by Li and Wu showed that in patients with UA, ZSXBGZ combined with conventional biomedical therapy was overall more effective than conventional biomedical therapy alone, and demonstrated relative advantages in overall efficacy and reduction of Hs-CRP levels compared with other botanical formulas (Li and Wu, 2024). Metabolomics-based cellular studies have further suggested that ZSXBGZ exerts cardioprotective effects against myocardial hypoxia by modulating oxidative stress and energy metabolism pathways (Lin et al., 2023).

### 4.2 TCM syndrome efficiency

Regarding TCM syndrome efficiency rate, all classical prescriptions combined with conventional biomedical therapy demonstrated statistically significant improvements compared with biomedical therapy alone in the overall analysis, suggesting a potential adjunctive benefit in syndrome-related symptom improvement. In the overall analysis, RS demonstrated a more favorable result in cumulative probability curve when combined with conventional biomedical therapy, suggesting that RS may be among the more promising adjunctive interventions. The major botanical drugs of RS include *Panax ginseng* C.A.Mey. [Araliaceae], *A. macrocephala* Koidz. [Asteraceae], *Zingiber officinale* Roscoe [Zingiberaceae] and *G. glabra* L. [Fabaceae]. Notably, partial overlap in botanical composition exists between RS and LGZG (including *S. glabra* Roxb [Smilacaceae], *N. cassia* (L.). Kosterm. [Lauraceae], *A. macrocephala* Koidz. [Asteraceae], *G. glabra* L. [Fabaceae]), which may suggest shared pharmacological orientations among prescriptions with similar botanical components. Evidence from previous studies provides partial support for the biological plausibility of RS for angina pectoris. A RCT reported that *P. ginseng* C.A.Mey. [Araliaceae] may improve vascular function by inhibiting ROCK signaling and reducing arterial stiffness in patients with coronary heart disease (Chung et al., 2010). A large cross-sectional study involving 4,628 participants suggested that dietary intake of *Z. officinale* Roscoe [Zingiberaceae] was associated with a lower risk of CHD [OR: 0.87, CI (0.78, 0.96)] (Wang et al., 2017). From a TCM perspective, classical indications of RS include“epigastric fullness and obstruction (xin xia pi)” as described in Synopsis of the Golden Chamber. In addition to classic substernal chest pain, patients may present with referred pain to the epigastric region, which can mimic gastrointestinal disorders such as gastritis or dyspepsia, as documented in clinical studies and cardiology guidelines (Haasenritter et al., 2012; Bellicini and Shams, 2026). Experimental evidence further suggests that RS may attenuate atherosclerosis progression in ApoE knockout mice, potentially through modulation of TRPV1 expression and activation of AMPK-related autophagy pathways (Qi et al., 2023).

The SAP subgroup results were generally aligned with the findings of the overall analysis in terms of effect direction, although some variability in probability distribution was observed, with GZFL showing comparatively higher SUCRA values. Previous meta-analyses have also suggested that GZFL-based interventions may improve angina pectoris efficiency and ECG-related outcomes when used as adjunctive therapy (Li et al., 2025). From a compositional perspective, GZFL contains botanical drugs including *S. glabra* Roxb [Smilacaceae], *N. cassia* (L.). Kosterm. [Lauraceae], *Paeonia × suffruticosa* Andrews [Paeoniaceae], *Paeonia veitchii* Lynch [Paeoniaceae], and *Juglans regia* L. [Juglandaceae]. Notably, *S. glabra* Roxb [Smilacaceae] and *N. cassia* (L.). Kosterm. [Lauraceae] are shared with LGZG, which may suggest partially overlapping therapeutic orientations among these prescriptions. Mechanistically, network pharmacology and *in vivo* pharmacodynamic assessment suggest that the combination of *S. glabra* Roxb [Smilacaceae] and *N. cassia* (L.). Kosterm. [Lauraceae] may reduce inflammatory responses by downregulating TNF-α and IL-6 expression, thereby potentially contributing to cardioprotective effects (Duan et al., 2020). In addition, cinnamaldehyde, a metabolite derived from *N. cassia* (L.) Kosterm. [Lauraceae], has been reported to exert vasodilatory effects on coronary arteries through inhibition of L-type calcium channel–mediated calcium influx and modulation of smooth muscle contractility in isolated porcine coronary artery models (Raffai et al., 2014).

Although all included classical prescriptions combined with conventional biomedical therapy demonstrated a consistent adjunctive benefit in this population, the ranking pattern in patients with UA showed a different leading intervention, with ZGC receiving a comparatively higher SUCRA value. Previous meta-analyses have suggested that ZGC-based interventions may improve angina symptoms, cardiac function, and vascular-related outcomes when used as adjunctive therapy. In patients with CHD, ZGC has been reported to improve lipid profiles, hemorheological parameters, and ECG findings (Zhou et al., 2023). In patients with diabetic cardiomyopathy, ZGC-based treatment has shown potential benefits in reducing systolic and diastolic blood pressure and improving left ventricular filling function (Ji et al., 2025). Additionally, holter monitoring in patients with UA showed that approximately 57% of patients developed severe arrhythmias during pain episodes, and some patients also exhibited a risk of arrhythmias even during asymptomatic periods (Lopes et al., 1975). Meta-analytic evidence has also supported the benefits of ZGC in patients with CHD complicated by arrhythmias, showing that the combination of ZGC and metoprolol can significantly reduce the frequency of premature beats (Huang et al., 2025). Evidence from pharmacological studies indicates that CCPP derived from ZGC may exert anti-atherosclerotic effects through modulation of ESR1 and NF-κB signaling pathways, thereby inhibiting foam cell formation (Fan et al., 2021).

### 4.3 Metabolic indicators

In lipid-related outcomes, only a limited number of classical prescriptions demonstrated a trend toward benefit when combined with conventional biomedical therapy compared with biomedical therapy alone. However, the direction and magnitude of these effects were inconsistent across different lipid parameters. For instance, GLXBBJ showed a statistically significant improvement in total TC, whereas no corresponding significant effect was observed for LDL-C, indicating the reliability and robustness of these lipid-related findings remain uncertain. In terms of inflammatory outcomes (Hs-CRP), inconsistency was also observed across analyses. The overall pooled analysis suggested a significant effect for GLXBBX (p < 0.05), whereas in the SAP subgroup, the observed effect was mainly driven by ZSXBGZ (p < 0.05), with no unified signal across subgroups. Due to insufficient data, subgroup analysis in UA could not be performed, further limiting cross-population validation of the findings. Preclinical studies have suggested that the anti-atherosclerotic effects of GLXBBJ may be associated with modulation of gut microbiota, particularly through increasing *Akkermansia* abundance and regulating microbiota-derived metabolites such as short-chain fatty acids and trimethylamine N-oxide. These changes may contribute to attenuation of vascular inflammation by reducing macrophage infiltration and inflammatory signaling, as well as by improving intestinal barrier integrity through upregulation of tight junction proteins, thereby decreasing systemic endotoxin exposure (Li et al., 2016; Chen et al., 2024). However, corresponding clinical evidence remains limited. Given the high degree of heterogeneity across included studies, the lack of consistency between overall and subgroup results, and the incomplete evidence structure across clinical sub-types, these findings should be interpreted cautiously.

### 4.4 Safety

In terms of safety outcomes, most classical prescriptions combined with conventional biomedical therapy, including GLXBBX, LGZG, RS, XXX, and ZSXBGZ, did not show statistically significant differences in adverse event incidence compared with conventional biomedical therapy alone. These findings suggest that, within the limitations of the available evidence, the overall safety profiles of these combinations appear to be comparable to standard treatment. Combination with GZFL was the only intervention associated with a significantly lower incidence of adverse events compared with conventional biomedical therapy alone, and it also ranked highly in SUCRA-based safety results. The relatively higher incidence of adverse events observed in some prescriptions may be partially associated with differences in their botanical composition. For example, *Pinellia ternata* (Thunb.) Makino [Araceae] is a shared botanical drug of GLXBBX, XXX, and ZSXBGZ. Although it is traditionally used for resolving phlegm and dampness, *P. ternata* (Thunb.) Makino [Araceae] contains irritant constituents such as calcium oxalate crystals and lectin proteins, which have been reported to cause adverse reactions if improperly processed or administered (Peng et al., 2022). Proper processing is essential to reduce potential toxicity and ensure clinical safety before use. Therefore, in real-world clinical practice, it is necessary to strictly adhere to the recommended use instructions and indications of classical prescriptions, and to closely monitor patients’ responses to treatment.

## 5 Limitations

This study has several limitations. (1) The structural characteristics of the evidence may influence the interpretation of indirect comparisons. The network was mainly star-shaped without closed loops, and no direct head-to-head comparisons among classical prescriptions via conventional biomedical therapy were available. Therefore, the comparative findings should be interpreted with appropriate caution. (2) The methodological reporting across included studies was not fully consistent. Randomization procedures were reported in 74 of 131 studies, while other aspects such as allocation concealment and blinding were not consistently described, which may introduce some uncertainty in internal validity. (3) Clinical heterogeneity was present across studies. Not all trials strictly followed syndrome differentiation-based treatment principles, and some interventions were applied across different TCM syndrome patterns of angina pectoris. In addition, variation in treatment duration across studies may have contributed to differences in observed outcomes. (4) The distribution of available evidence was uneven across interventions. Several prescriptions (RS, XCH, SN, and ZGC) were evaluated in a relatively small number of studies, which may limit the stability of pooled estimates for these interventions. Moreover, not all studies reported the same outcome indicators, leading to incomplete outcome coverage. All included RCTs were conducted in China, which may limit the broader applicability of the findings. (5). For lipid-related and inflammatory outcomes, variability was observed across studies, and the interpretability of pooled results may be influenced by differences in study design, population characteristics, and outcome measurement.

Although classical prescriptions are widely used in clinical practice based on historical experience and clinical observations, the overall level of clinical evidence remains limited. Future well-designed, multicenter randomized controlled trials with standardized methodologies and outcome definitions are needed to further clarify these findings.

## 6 Conclusion

The findings of this study suggest that most TCM classical prescriptions, when used as adjunctive therapy, may be associated with more favorable outcomes in improving angina pectoris efficiency, ECG efficiency, and TCM syndrome efficiency compared with conventional biomedical treatment alone. No clear differences in safety outcomes were observed between most classical prescriptions and conventional biomedical therapy. Notably, the current evidence remains insufficient to draw firm conclusions regarding the effects of classical prescriptions on lipid- and inflammation-related outcomes when used as adjunctive therapy.

In the evaluated interventions, exploratory patterns in comparative probabilities were observed; however, due to the open network structure, these results should be interpreted cautiously. Further high-quality, well-designed clinical trials are required to clarify these findings and strengthen the evidence base.

## Data Availability

The original contributions presented in the study are included in the article/Supplementary Material, further inquiries can be directed to the corresponding authors.
