# Supplementary material for: Comparative efficacy of Traditional Chinese Medicine classical prescriptions for angina pectoris: a network meta-analysis
Source: Front Pharmacol. 2026 Sep 28;17:1794371. doi: 10.3389/fphar.2026.1794371 (PMC13654956; doi:10.3389/fphar.2026.1794371)
Supplement: Supplementary file 1 [file DataSheet1.ZIP › Supplementary material/data1_TableS1-S2_botanical drug information.docx]

**supplementary material**

**Table S1** The botanical drug(s) involved.

| Chinese name | Accepted name |
| --- | --- |
| baibu | Stemona tuberosa Lour. [Stemonaceae] |
| baishao | Cynanchum otophyllum C.K.Schneid. [Apocynaceae] |
| baizhu | Atractylodes macrocephala Koidz. [Asteraceae] |
| bajitian | Gynochthodes officinalis (F.C.How) Razafim. & B.Bremer [Rubiaceae] |
| bajitian | Morindae Officinalis Radix |
| banxia | Pinellia ternata (Thunb.) Makino [Araceae] |
| chaihu | Elephantopus scaber L. [Asteraceae] |
| chenpi | Citrus reticulata Blanco [Rutaceae] |
| cheqianzi | Plantago asiatica L. [Plantaginaceae] |
| chishao | Paeonia veitchii Lynch [Paeoniaceae] |
| chuanxiong | Conioselinum anthriscoides 'Chuanxiong' [Apiaceae] |
| danggui | Angelica sinensis (Oliv.) Diels [Apiaceae] |
| dangshen | Codonopsis pilosula (Franch.) Nannf. [Campanulaceae] |
| dannanxing | Arisaema amurense Maxim. [Araceae] |
| danshen | Salvia miltiorrhiza Bunge [Lamiaceae; Salviae miltiorrhizae radix et rhizoma] |
| daxueteng | Sargentodoxa cuneata (Oliv.) Rehder & E.H.Wilson [Lardizabalaceae] |
| dazao | Ziziphus jujuba Mill. [Rhamnaceae] |
| fuling | Smilax glabra Roxb. [Smilacaceae] |
| ganjiang | Zingiber officinale Roscoe [Zingiberaceae] |
| gancao | Glycyrrhiza glabra L. [Fabaceae] |
| gegen | Pueraria montana var. lobata (Willd.) Maesen & S.M.Almeida ex Sanjappa & Predeep [Fabaceae] |
| gualou | Trichosanthes kirilowii Maxim. [Cucurbitaceae] |
| guizhi | Neolitsea cassia (L.) Kosterm. [Lauraceae] |
| honghua | Carthamus tinctorius L. [Asteraceae] |
| hongshen | Panax ginseng C.A.Mey. [Araliaceae] |
| houpo | Magnolia officinalis Rehder & E.H.Wilson [Magnoliaceae] |
| huangjiu | Vinum |
| huanglian | Picrorhiza kurrooa Royle [Plantaginaceae] |
| huangqi | Astragalus mongholicus Bunge [Fabaceae] |
| huluba | Trigonella foenum-graecum L. [Fabaceae] |
| jiangxiang | Dalbergia odorifera T.C.Chen [Fabaceae] |
| jiegeng | Platycodon grandiflorus (Jacq.) A.DC. [Campanulaceae] |
| jixueteng | Spatholobus suberectus Dunn [Fabaceae] |
| juhong | Citrus reticulata Blanco [Rutaceae] |
| lianqiao | Hypericum perforatum L. [Hypericaceae] |
| maidong | Ophiopogon japonicus (Thunb.) Ker Gawl. [Asparagaceae] |
| mudanpi | Paeonia × suffruticosa Andrews [Paeoniaceae] |
| muxiang | Dolomiaea costus (Falc.) Kasana & A.K.Pandey [Asteraceae] |
| renshen | Panax ginseng C.A.Mey. [Araliaceae] |
| sangzhi | Morus alba L. [Moraceae] |
| sangzhi | Mori Ramulus |
| sanqi | Kalanchoe pinnata (Lam.) Pers. [Crassulaceae] |
| shanzha | Crataegus pinnatifida Bunge [Rosaceae] |
| sharen | Wurfbainia villosa (Lour.) Škorničk. & A.D.Poulsen [Zingiberaceae] |
| sharen | Amomi Fructus |
| shengdihuang | Rehmannia glutinosa (Gaertn.) Libosch. ex DC. [Orobanchaceae] |
| shengpuhuang | Typha angustifolia L. [Typhaceae] |
| shichangpu | Acorus gramineus Aiton [Acoraceae] |
| suanzaoren | Ziziphus jujuba subsp. spinosa (Bunge) J.Y.Peng, X.Y.Li & L.Li [Rhamnaceae] |
| tanxiang | Santalum album L. [Santalaceae] |
| taoren | Juglans regia L. [Juglandaceae] |
| tongcao | Tetrapanax papyrifer (Hook.) K.Koch [Araliaceae] |
| xianhecao | Agrimonia pilosa Ledeb. [Rosaceae] |
| xiebai | Allium chinense G.Don [Amaryllidaceae] |
| xixin | Asarum heterotropoides F.Schmidt [Aristolochiaceae] |
| xiyangshen | Panax quinquefolius L. [Araliaceae] |
| yanhusuo | Corydalis yanhusuo (Y.H.Chou & Chun C.Hsu) W.T.Wang ex Z.Y.Su & C.Y.Wu [Papaveraceae] |
| yiyiren | Coix lacryma-jobi L. [Poaceae] |
| yujin | Curcuma longa L. [Zingiberaceae] |
| zelan | Eupatorium japonicum Thunb. [Asteraceae] |
| zhechong | Eupolyphaga sinensis Walker |
| zhechong | Eupolyphaga |
| zhiqiao | Citrus × aurantium f. aurantium [Rutaceae] |
| zhuru | Bambusa tuldoides Munro [Poaceae] |
| zisugeng | Perilla frutescens (L.) Britton [Lamiaceae] |
| baibu | Stemona tuberosa Lour. [Stemonaceae] |

**Table S2** The composition of each decoction.

| Study | Decoction name | Authoritative source / origin stated in article | Composition reported in the included study | Relevant potential species | Preparation / administration reported |
| --- | --- | --- | --- | --- | --- |
| CHEN XB 2020 | Modified DangguiSini Tang | *Treatise on Typhoid Fever* | Angelicae Sinensis Radix 20g, Spatholobi Caulis 20g, Cinnamomi Ramulus 10g, Asari Radix et Rhizoma 10g, Tetrapanacis Medulla 10g, Paeoniae Radix Alba 10g, Pheretima 10g, Glycyrrhizae Radix et Rhizoma 10g, Astragali Radix 30g, Jujubae Fructus 3 pieces | Neolitsea cassia (L.) Kosterm. [Lauraceae]; Spatholobus suberectus Dunn [Fabaceae]; Angelica sinensis (Oliv.) Diels [Apiaceae]; Asarum heterotropoides F.Schmidt [Aristolochiaceae]; Tetrapanax papyrifer (Hook.) K.Koch [Araliaceae]; Cynanchum otophyllum C.K.Schneid. [Apocynaceae]; Pheretima; Glycyrrhiza uralensis Fisch. ex DC. [Fabaceae]; Astragalus mongholicus Bunge [Fabaceae]; Ziziphus jujuba Mill. [Rhamnaceae] | One dose per day, taken twice daily. The treatment observation period is 4 weeks. |
| CUI JK 2020 | Modified Dangguisini Tang | *Treatise on Typhoid Fever* | Angelicae Sinensis Radix 20 g, Cinnamomi Ramulus 11 g, Asari Radix et Rhizoma 5 g, Tetrapanacis Medulla 5 g, Paeoniae Radix Alba 20 g, Jujubae Fructus 3 pieces, Glycyrrhizae Radix et Rhizoma Praeparata 10 g, Astragali Radix 15 g, Corydalis Rhizoma 15 g | Neolitsea cassia (L.) Kosterm. [Lauraceae]; Angelica sinensis (Oliv.) Diels [Apiaceae]; Asarum heterotropoides F.Schmidt [Aristolochiaceae]; Tetrapanax papyrifer (Hook.) K.Koch [Araliaceae]; Cynanchum otophyllum C.K.Schneid. [Apocynaceae]; Ziziphus jujuba Mill. [Rhamnaceae]; Glycyrrhiza uralensis Fisch. ex DC. [Fabaceae]; Astragalus mongholicus Bunge [Fabaceae]; Corydalis yanhusuo (Y.H.Chou & Chun C.Hsu) W.T.Wang ex Z.Y.Su & C.Y.Wu [Papaveraceae] | One dose per day, taken twice daily. The treatment observation period is 4 weeks. |
| DU H 2022 | Modified Dangguisini Tang | *Treatise on Typhoid Fever* | Salviae Miltiorrhizae Radix et Rhizoma 30 g, Astragali Radix 30 g, Angelicae Sinensis Radix 20 g, Allii Macrostemonis Bulbus 15 g, Aurantii Fructus Immaturus 15 g, Paeoniae Radix Alba 12 g, Jujubae Fructus 12 pieces, Glycyrrhizae Radix et Rhizoma Praeparata 10 g, Zingiberis Rhizoma Recens 10 g, Cinnamomi Ramulus 10 g, Tetrapanacis Medulla 6 g, Asari Radix et Rhizoma 3 g | Salvia miltiorrhiza Bunge [Lamiaceae; Salviae miltiorrhizae radix et rhizoma]; Astragalus mongholicus Bunge [Fabaceae]; Neolitsea cassia (L.) Kosterm. [Lauraceae]; Allium chinense G.Don [Amaryllidaceae]; Citrus × aurantium f. aurantium [Rutaceae]; Cynanchum otophyllum C.K.Schneid. [Apocynaceae]; Ziziphus jujuba Mill. [Rhamnaceae]; Glycyrrhiza uralensis Fisch. ex DC. [Fabaceae]; Zingiber officinale Roscoe [Zingiberaceae]; Angelica sinensis (Oliv.) Diels [Apiaceae]; Tetrapanax papyrifer (Hook.) K.Koch [Araliaceae]; Asarum heterotropoides F.Schmidt [Aristolochiaceae] | One dose per day, taken twice daily (300ml per time). The treatment observation period is 2 weeks. |
| JIA XY 2020 | Modified Dangguisini Tang | *Treatise on Typhoid Fever* | Angelicae Sinensis Radix 20 g, Cinnamomi Ramulus 10 g, Asari Radix et Rhizoma 3 g, Aurantii Fructus Immaturus 15 g, Allii Macrostemonis Bulbus 15 g, Paeoniae Radix Alba 12 g, Glycyrrhizae Radix et Rhizoma Praeparata 10 g, Tetrapanacis Medulla 6 g, Jujubae Fructus 12 g, Salviae Miltiorrhizae Radix et Rhizoma 30 g, Astragali Radix 30 g, Zingiberis Rhizoma Recens 10 g | Neolitsea cassia (L.) Kosterm. [Lauraceae]; Angelica sinensis (Oliv.) Diels [Apiaceae]; Asarum heterotropoides F.Schmidt [Aristolochiaceae]; Citrus × aurantium f. aurantium [Rutaceae]; Allium chinense G.Don [Amaryllidaceae]; Cynanchum otophyllum C.K.Schneid. [Apocynaceae]; Glycyrrhiza uralensis Fisch. ex DC. [Fabaceae]; Tetrapanax papyrifer (Hook.) K.Koch [Araliaceae]; Ziziphus jujuba Mill. [Rhamnaceae]; Salvia miltiorrhiza Bunge [Lamiaceae; Salviae miltiorrhizae radix et rhizoma]; Astragalus mongholicus Bunge [Fabaceae]; Zingiber officinale Roscoe [Zingiberaceae] | One dose per day, taken twice daily (200ml per time). The treatment observation period is 2 weeks. |
| SUN XH 2022 | Modified Dangguisini Tang | *Treatise on Typhoid Fever* | Paeoniae Radix Rubra 12 g, Paeoniae Radix Alba 12 g, Sargentodoxae Caulis 12 g, Salviae Miltiorrhizae Radix et Rhizoma 12 g, Cinnamomi Ramulus 9 g, Tetrapanacis Medulla 6 g, Glycyrrhizae Radix et Rhizoma Praeparata 6 g, Asari Radix et Rhizoma 3 g, Jujubae Fructus 8 pieces | Paeonia veitchii Lynch [Paeoniaceae]; Cynanchum otophyllum C.K.Schneid. [Apocynaceae]; Sargentodoxa cuneata (Oliv.) Rehder & E.H.Wilson [Lardizabalaceae]; Salvia miltiorrhiza Bunge [Lamiaceae; Salviae miltiorrhizae radix et rhizoma]; Angelica sinensis (Oliv.) Diels [Apiaceae]; Tetrapanax papyrifer (Hook.) K.Koch [Araliaceae]; Glycyrrhiza uralensis Fisch. ex DC. [Fabaceae]; Asarum heterotropoides F.Schmidt [Aristolochiaceae]; Ziziphus jujuba Mill. [Rhamnaceae] | One dose per day, taken twice daily (150ml per time). The treatment observation period is 3 weeks. |
| YUAN QM 2020 | Modified Dangguisini Tang | *Treatise on Typhoid Fever* | Angelicae Sinensis Radix 12 g, Cinnamomi Ramulus 9 g, Paeoniae Radix Alba 9 g, Asari Radix et Rhizoma 3 g, Tetrapanacis Medulla 6 g, Jujubae Fructus 8 pieces, Glycyrrhizae Radix et Rhizoma Praeparata 6 g | Neolitsea cassia (L.) Kosterm. [Lauraceae]; Angelica sinensis (Oliv.) Diels [Apiaceae]; Cynanchum otophyllum C.K.Schneid. [Apocynaceae]; Asarum heterotropoides F.Schmidt [Aristolochiaceae]; Tetrapanax papyrifer (Hook.) K.Koch [Araliaceae]; Ziziphus jujuba Mill. [Rhamnaceae]; Glycyrrhiza uralensis Fisch. ex DC. [Fabaceae] | One dose per day, taken twice daily. The treatment observation period is 4 weeks. |
| CHEN LP 2022 | Modified Gualou Xiebai Baijiu Tang | *Synopsis of Golden Chamber* | Trichosanthis Fructus 12 g, Allii Macrostemonis Bulbus 12 g, Vinum (Bai jiu) 50 mL | Trichosanthes kirilowii Maxim. [Cucurbitaceae]; Allium chinense G.Don [Amaryllidaceae]; Vinum | The treatment observation period is 20 days. |
| HE MX 2017 | Modified Gualou Xiebai Baijiu Tang | *Synopsis of Golden Chamber* | Trichosanthis Fructus 20 g, Angelicae Sinensis Radix 10 g, Schisandrae Chinensis Fructus 10 g, Bupleuri Radix 10 g, Salviae Miltiorrhizae Radix et Rhizoma 10 g, Ophiopogonis Radix 15 g, Allii Macrostemonis Bulbus 15 g, Paeoniae Radix Rubra 15 g, Curcumae Radix 15 g, Ziziphi Spinosae Semen Preparata 8 g, Ginseng Radix et Rhizoma Rubra 8 g, Panacis Quinquefolii Radix 8 g, Aucklandiae Radix 8 g, Glycyrrhizae Radix et Rhizoma 6 g, Notoginseng Radix et Rhizoma 4 g, Astragali Radix 30 g, Vinum (Bai jiu) appropriate amount | Trichosanthes kirilowii Maxim. [Cucurbitaceae]; Neolitsea cassia (L.) Kosterm. [Lauraceae]; Schisandra chinensis (Turcz.) Baill. [Schisandraceae]; Elephantopus scaber L. [Asteraceae]; Salvia miltiorrhiza Bunge [Lamiaceae; Salviae miltiorrhizae radix et rhizoma]; Ophiopogon japonicus (Thunb.) Ker Gawl. [Asparagaceae]; Allium chinense G.Don [Amaryllidaceae]; Paeonia veitchii Lynch [Paeoniaceae]; Curcuma longa L. [Zingiberaceae]; Ziziphus jujuba subsp. spinosa (Bunge) J.Y.Peng, X.Y.Li & L.Li [Rhamnaceae]; Panax ginseng C.A.Mey. [Araliaceae]; Panax quinquefolius L. [Araliaceae]; Dolomiaea costus (Falc.) Kasana & A.K.Pandey [Asteraceae]; Glycyrrhiza uralensis Fisch. ex DC. [Fabaceae]; Kalanchoe pinnata (Lam.) Pers. [Crassulaceae]; Astragalus mongholicus Bunge [Fabaceae]; Vinum | One dose per day, taken twice daily. The treatment observation period is 75 days. |
| HU J 2018 | Modified Gualou Xiebai Baijiu Tang | *Synopsis of Golden Chamber* | Hirudo 5 g, Vinum (Bai jiu) 5 g, Chuanxiong Rhizoma 15 g, Platycodonis Radix 15 g, Achyranthis Bidentatae Radix 15 g, Trichosanthis Fructus 15 g, Allii Macrostemonis Bulbus 15 g, Paeoniae Radix Rubra 15 g, Carthami Flos 15 g, Rehmanniae Radix 20 g | Whitmania pigra Whitman; Vinum; Conioselinum anthriscoides 'Chuanxiong' [Apiaceae]; Platycodon grandiflorus (Jacq.) A.DC. [Campanulaceae]; Achyranthes bidentata Blume [Amaranthaceae]; Trichosanthes kirilowii Maxim. [Cucurbitaceae]; Allium chinense G.Don [Amaryllidaceae]; Paeonia veitchii Lynch [Paeoniaceae]; Carthamus tinctorius L. [Asteraceae]; Rehmannia glutinosa (Gaertn.) Libosch. ex DC. [Orobanchaceae] | One dose per day, taken twice daily. The treatment observation period is 2 weeks. |
| LI SB 2015 | Modified Gualou Xiebai Baijiu Tang | *Synopsis of Golden Chamber* | Allii Macrostemonis Bulbus 10 g, Trichosanthis Fructus 30 g, Vinum (Huangjiu) 60 mL, Magnoliae Officinalis Cortex 10 g, Salviae Miltiorrhizae Radix et Rhizoma 30 g, Pinelliae Rhizoma 10 g, Angelicae Sinensis Radix 15 g, Chuanxiong Rhizoma 10 g, Notoginseng Radix et Rhizoma 10 g, Aurantii Fructus 10 g, Typhae Pollen (raw, wrapped in cloth) 10 g, Pheretima 10 g, Santali Albi Lignum 6 g, Cinnamomi Ramulus 10 g, Curcumae Radix 10 g | Allium chinense G.Don [Amaryllidaceae]; Trichosanthes kirilowii Maxim. [Cucurbitaceae]; Vinum; Magnolia officinalis Rehder & E.H.Wilson [Magnoliaceae]; Salvia miltiorrhiza Bunge [Lamiaceae; Salviae miltiorrhizae radix et rhizoma]; Pinellia ternata (Thunb.) Makino [Araceae]; Neolitsea cassia (L.) Kosterm. [Lauraceae]; Conioselinum anthriscoides 'Chuanxiong' [Apiaceae]; Kalanchoe pinnata (Lam.) Pers. [Crassulaceae]; Citrus × aurantium f. aurantium [Rutaceae]; Typha angustifolia L. [Typhaceae]; Pheretima; Santalum album L. [Santalaceae]; Angelica sinensis (Oliv.) Diels [Apiaceae]; Curcuma longa L. [Zingiberaceae] | One dose per day, taken twice daily (200ml per time). The treatment observation period is 2 weeks. |
| YANG WQ 2021 | Modified Gualou Xiebai Baijiu Tang | *Synopsis of Golden Chamber* | Angelicae Sinensis Radix 15 g, Pinelliae Rhizoma 10 g, Magnoliae Officinalis Cortex 10 g, Allii Macrostemonis Bulbus 10 g, Salviae Miltiorrhizae Radix et Rhizoma 30 g, Vinum (Huangjiu) 60 mL, Notoginseng Radix et Rhizoma 10 g, Trichosanthis Fructus 30 g, Typhae Pollen Crudum 10 g, Chuanxiong Rhizoma 10 g, Santali Albi Lignum 6 g, Aurantii Fructus 10 g, Curcumae Radix 10 g, Pheretima 10 g, Cinnamomi Ramulus 10 g | Neolitsea cassia (L.) Kosterm. [Lauraceae]; Pinellia ternata (Thunb.) Makino [Araceae]; Magnolia officinalis Rehder & E.H.Wilson [Magnoliaceae]; Allium chinense G.Don [Amaryllidaceae]; Salvia miltiorrhiza Bunge [Lamiaceae; Salviae miltiorrhizae radix et rhizoma]; Vinum; Kalanchoe pinnata (Lam.) Pers. [Crassulaceae]; Trichosanthes kirilowii Maxim. [Cucurbitaceae]; Typha angustifolia L. [Typhaceae]; Conioselinum anthriscoides 'Chuanxiong' [Apiaceae]; Santalum album L. [Santalaceae]; Citrus × aurantium f. aurantium [Rutaceae]; Curcuma longa L. [Zingiberaceae]; Pheretima; Angelica sinensis (Oliv.) Diels [Apiaceae] | One dose per day, taken twice daily (200ml per time). The treatment observation period is 2 months. |
| YUE SM 2021 | Modified Gualou Xiebai Baijiu Tang | *Synopsis of Golden Chamber* | Trichosanthis Fructus 30 g, Vinum (Bai jiu) 60 mL, Allii Macrostemonis Bulbus 10 g, Angelicae Sinensis Radix 15 g, Magnoliae Officinalis Cortex 10 g, Pinelliae Rhizoma 10 g, Pheretima 10 g, Chuanxiong Rhizoma 10 g | Trichosanthes kirilowii Maxim. [Cucurbitaceae]; Vinum; Allium chinense G.Don [Amaryllidaceae]; Neolitsea cassia (L.) Kosterm. [Lauraceae]; Magnolia officinalis Rehder & E.H.Wilson [Magnoliaceae]; Pinellia ternata (Thunb.) Makino [Araceae]; Pheretima; Conioselinum anthriscoides 'Chuanxiong' [Apiaceae] | One dose per day, taken twice daily (100ml per time). The treatment observation period is 2 weeks. |
| CAO PZ 2021 | Modified Gualou Xiebai Banxia Tang | *Synopsis of Golden Chamber* | Astragali Radix Praeparata 30 g, Trichosanthis Fructus 20 g, Allii Macrostemonis Bulbus 15 g, Pinelliae Rhizoma Praeparatum cum Zingibere 15 g, Poria 15 g, Atractylodis Macrocephalae Rhizoma 15 g, Salviae Miltiorrhizae Radix et Rhizoma 15 g | Astragalus mongholicus Bunge [Fabaceae]; Trichosanthes kirilowii Maxim. [Cucurbitaceae]; Allium chinense G.Don [Amaryllidaceae]; Pinellia ternata (Thunb.) Makino [Araceae]; Smilax glabra Roxb. [Smilacaceae]; Atractylodes macrocephala Koidz. [Asteraceae]; Salvia miltiorrhiza Bunge [Lamiaceae; Salviae miltiorrhizae radix et rhizoma] | One dose per day, taken twice daily . The treatment observation period is 4 weeks. |
| QIU WF 2021 | Modified Gualou Xiebai Banxia Tang | *Synopsis of Golden Chamber* | Trichosanthis Fructus 15 g, Allii Macrostemonis Bulbus 10 g, Pinelliae Rhizoma Praeparatum 6 g, Cinnamomi Ramulus 6 g, Salviae Miltiorrhizae Radix et Rhizoma 30 g, Persicae Semen 12 g, Chuanxiong Rhizoma 9 | Trichosanthes kirilowii Maxim. [Cucurbitaceae]; Allium chinense G.Don [Amaryllidaceae]; Pinellia ternata (Thunb.) Makino [Araceae]; Angelica sinensis (Oliv.) Diels [Apiaceae]; Salvia miltiorrhiza Bunge [Lamiaceae; Salviae miltiorrhizae radix et rhizoma]; Juglans regia L. [Juglandaceae]; Conioselinum anthriscoides 'Chuanxiong' [Apiaceae] | One dose per day, taken twice daily . The treatment observation period is 1 months. |
| DU FC 2022 | Modified Gualou Xiebai Banxia Tang | *Synopsis of Golden Chamber* | Trichosanthis Fructus 30 g, Paeoniae Radix Rubra 20 g, Pinelliae Rhizoma Praeparatum 20 g, Salviae Miltiorrhizae Radix et Rhizoma 20 g, Pheretima 15 g, Corydalis Rhizoma 15 g, Angelicae Sinensis Radix 15 g, Poria 15 g, Citri Reticulatae Pericarpium 15 g, Platycodonis Radix 15 g, Glycyrrhizae Radix et Rhizoma Praeparata 10 g, Allii Macrostemonis Bulbus 10 g | Trichosanthes kirilowii Maxim. [Cucurbitaceae]; Paeonia veitchii Lynch [Paeoniaceae]; Pinellia ternata (Thunb.) Makino [Araceae]; Salvia miltiorrhiza Bunge [Lamiaceae; Salviae miltiorrhizae radix et rhizoma]; Pheretima; Corydalis yanhusuo (Y.H.Chou & Chun C.Hsu) W.T.Wang ex Z.Y.Su & C.Y.Wu [Papaveraceae]; Neolitsea cassia (L.) Kosterm. [Lauraceae]; Smilax glabra Roxb. [Smilacaceae]; Citrus reticulata Blanco [Rutaceae]; Platycodon grandiflorus (Jacq.) A.DC. [Campanulaceae]; Glycyrrhiza uralensis Fisch. ex DC. [Fabaceae]; Allium chinense G.Don [Amaryllidaceae] | One dose per day, taken twice daily (150ml per time) . The treatment observation period is 8 weeks. |
| DU Y 2022 | Modified Gualou Xiebai Banxia Tang | *Synopsis of Golden Chamber* | Cinnamomi Ramulus 10 g, Allii Macrostemonis Bulbus 10 g, Pinelliae Rhizoma Praeparatum 10 g, Trichosanthis Fructus 15 g, Citri Reticulatae Pericarpium 15 g, Acori Tatarinowii Rhizoma 15 g, Salviae Miltiorrhizae Radix et Rhizoma 30 g | Angelica sinensis (Oliv.) Diels [Apiaceae]; Allium chinense G.Don [Amaryllidaceae]; Pinellia ternata (Thunb.) Makino [Araceae]; Trichosanthes kirilowii Maxim. [Cucurbitaceae]; Citrus reticulata Blanco [Rutaceae]; Acorus gramineus Aiton [Acoraceae]; Salvia miltiorrhiza Bunge [Lamiaceae; Salviae miltiorrhizae radix et rhizoma] | One dose per day, taken twice daily . The treatment observation period is 1 month. |
| GAN TM 2015 | Modified Gualou Xiebai Banxia Tang | *Synopsis of Golden Chamber* | Trichosanthis Fructus 15 g, Allii Macrostemonis Bulbus 10 g, Pinelliae Rhizoma 10 g, Codonopsis Radix 15 g, Cinnamomi Ramulus 10 g, Curcumae Radix 10 g, Salviae Miltiorrhizae Radix et Rhizoma 15 g | Trichosanthes kirilowii Maxim. [Cucurbitaceae]; Allium chinense G.Don [Amaryllidaceae]; Pinellia ternata (Thunb.) Makino [Araceae]; Codonopsis pilosula (Franch.) Nannf. [Campanulaceae]; Angelica sinensis (Oliv.) Diels [Apiaceae]; Curcuma longa L. [Zingiberaceae]; Salvia miltiorrhiza Bunge [Lamiaceae; Salviae miltiorrhizae radix et rhizoma] | One dose per day, taken twice daily . The treatment observation period is 30 days. |
| GU Y 2022 | Modified Gualou Xiebai Banxia Tang | *Synopsis of Golden Chamber* | Coptidis Rhizoma 7 g, Trichosanthis Fructus 15 g, Allii Macrostemonis Bulbus 15 g, Pinelliae Rhizoma Praeparatum cum Zingibere 12 g, Aurantii Fructus Immaturus 12 g, Magnoliae Officinalis Cortex 12 g, Poria 12 g, Chuanxiong Rhizoma 12 g, Glycyrrhizae Radix et Rhizoma 10 g, Zingiberis Rhizoma Recens 3 slices, Cinnamomi Ramulus 12 g, Paeoniae Radix Rubra 15 g, Draconis Os Crudum 30 g, Ostreae Concha Cruda 30 g | Picrorhiza kurrooa Royle [Plantaginaceae]; Trichosanthes kirilowii Maxim. [Cucurbitaceae]; Allium chinense G.Don [Amaryllidaceae]; Pinellia ternata (Thunb.) Makino [Araceae]; Citrus × aurantium f. aurantium [Rutaceae]; Magnolia officinalis Rehder & E.H.Wilson [Magnoliaceae]; Smilax glabra Roxb. [Smilacaceae]; Conioselinum anthriscoides 'Chuanxiong' [Apiaceae]; Glycyrrhiza uralensis Fisch. ex DC. [Fabaceae]; Zingiber officinale Roscoe [Zingiberaceae]; Angelica sinensis (Oliv.) Diels [Apiaceae]; Paeonia veitchii Lynch [Paeoniaceae]; Os Dracons; Ostreae Concha | One dose per day, taken three times daily (100ml per time). The treatment observation period is 4 weeks. |
| GUO CJ 2024 | Modified Gualou Xiebai Banxia Tang | *Synopsis of Golden Chamber* | Carthami Flos 10 g, Chuanxiong Rhizoma 15 g, Persicae Semen 15 g, Allii Macrostemonis Bulbus 15 g, Pheretima 15 g, Paeoniae Radix Rubra 15 g, Pinelliae Rhizoma 15 g, Trichosanthis Fructus 15 g, Astragali Radix 20 g | Carthamus tinctorius L. [Asteraceae]; Conioselinum anthriscoides 'Chuanxiong' [Apiaceae]; Juglans regia L. [Juglandaceae]; Allium chinense G.Don [Amaryllidaceae]; Pheretima; Paeonia veitchii Lynch [Paeoniaceae]; Pinellia ternata (Thunb.) Makino [Araceae]; Trichosanthes kirilowii Maxim. [Cucurbitaceae]; Astragalus mongholicus Bunge [Fabaceae] | One dose per day. The treatment observation period is 4 weeks. |
| HE J 2017 | Modified Gualou Xiebai Banxia Tang | *Synopsis of Golden Chamber* | Aurantii Fructus Immaturus 9 g, Bambusae Caulis in Taeniam 9 g, Pinelliae Rhizoma Praeparatum 9 g, Allii Macrostemonis Bulbus 15 g, Poria 15 g, Citri Reticulatae Pericarpium 15 g, Angelicae Sinensis Radix 15 g | Citrus × aurantium f. aurantium [Rutaceae]; Bambusa tuldoides Munro [Poaceae]; Pinellia ternata (Thunb.) Makino [Araceae]; Allium chinense G.Don [Amaryllidaceae]; Smilax glabra Roxb. [Smilacaceae]; Citrus reticulata Blanco [Rutaceae]; Neolitsea cassia (L.) Kosterm. [Lauraceae] | One dose per day, taken tiwce daily (150ml per time). The treatment observation period is 2 weeks. |
| HUANG PP 2020 | Modified Gualou Xiebai Banxia Tang | *Synopsis of Golden Chamber* | Trichosanthis Fructus 25 g, Allii Macrostemonis Bulbus 15 g, Salviae Miltiorrhizae Radix et Rhizoma 15 g, Pinelliae Rhizoma Praeparatum 10 g, Cinnamomi Ramulus 10 g, Bupleuri Radix 10 g, Curcumae Radix 10 g, Corydalis Rhizoma 10 g | Trichosanthes kirilowii Maxim. [Cucurbitaceae]; Allium chinense G.Don [Amaryllidaceae]; Salvia miltiorrhiza Bunge [Lamiaceae; Salviae miltiorrhizae radix et rhizoma]; Pinellia ternata (Thunb.) Makino [Araceae]; Angelica sinensis (Oliv.) Diels [Apiaceae]; Elephantopus scaber L. [Asteraceae]; Curcuma longa L. [Zingiberaceae]; Corydalis yanhusuo (Y.H.Chou & Chun C.Hsu) W.T.Wang ex Z.Y.Su & C.Y.Wu [Papaveraceae] | One dose per day, taken tiwce daily (200 ml per time). The treatment observation period is 4 weeks. |
| JIANG A 2013 | Modified Gualou Xiebai Banxia Tang | *Synopsis of Golden Chamber* | Trichosanthis Fructus 20 g, Pinelliae Rhizoma Praeparatum 10 g, Allii Macrostemonis Bulbus 10 g, Citri Reticulatae Pericarpium 10 g, Magnoliae Officinalis Cortex 10 g, Curcumae Radix 10 g, Salviae Miltiorrhizae Radix et Rhizoma 15 g, Acori Tatarinowii Rhizoma 10 g, Glycyrrhizae Radix et Rhizoma 6 g, Fritillariae Cirrhosae Bulbus 12 g, Houttuyniae Herba 30 g, Stemonae Radix Praeparata 10 g | Trichosanthes kirilowii Maxim. [Cucurbitaceae]; Pinellia ternata (Thunb.) Makino [Araceae]; Allium chinense G.Don [Amaryllidaceae]; Citrus reticulata Blanco [Rutaceae]; Magnolia officinalis Rehder & E.H.Wilson [Magnoliaceae]; Curcuma longa L. [Zingiberaceae]; Salvia miltiorrhiza Bunge [Lamiaceae; Salviae miltiorrhizae radix et rhizoma]; Acorus gramineus Aiton [Acoraceae]; Glycyrrhiza uralensis Fisch. ex DC. [Fabaceae]; Fritillaria cirrhosa D.Don [Liliaceae]; Houttuynia cordata Thunb. [Saururaceae]; Stemona tuberosa Lour. [Stemonaceae] | One dose per day, taken tiwce daily. The treatment observation period is 2 weeks. |
| LI JL 2009 | Modified Gualou Xiebai Banxia Tang | *Synopsis of Golden Chamber* | Trichosanthis Fructus 15 g, Curcumae Radix 15 g, Allii Macrostemonis Bulbus 12 g, Pinelliae Rhizoma 12 g, Aurantii Fructus 12 g, Notoginseng Radix et Rhizoma 3 g, Crataegi Fructus 15 g, Salviae Miltiorrhizae Radix et Rhizoma 30 g, Chuanxiong Rhizoma 12 g | Trichosanthes kirilowii Maxim. [Cucurbitaceae]; Curcuma longa L. [Zingiberaceae]; Allium chinense G.Don [Amaryllidaceae]; Pinellia ternata (Thunb.) Makino [Araceae]; Citrus × aurantium f. aurantium [Rutaceae]; Kalanchoe pinnata (Lam.) Pers. [Crassulaceae]; Crataegus pinnatifida Bunge [Rosaceae]; Salvia miltiorrhiza Bunge [Lamiaceae; Salviae miltiorrhizae radix et rhizoma]; Conioselinum anthriscoides 'Chuanxiong' [Apiaceae] | One dose per day, taken tiwce daily. The treatment observation period is 4 weeks. |
| LI RM 2020 | Modified Gualou Xiebai Banxia Tang | *Synopsis of Golden Chamber* | Astragali Radix 30 g, Trichosanthis Fructus 20 g, Pinelliae Rhizoma Praeparatum cum Zingibere 15 g, Poria 15 g, Atractylodis Macrocephalae Rhizoma 15 g, Salviae Miltiorrhizae Radix et Rhizoma 15 g | Astragalus mongholicus Bunge [Fabaceae]; Trichosanthes kirilowii Maxim. [Cucurbitaceae]; Pinellia ternata (Thunb.) Makino [Araceae]; Smilax glabra Roxb. [Smilacaceae]; Atractylodes macrocephala Koidz. [Asteraceae]; Salvia miltiorrhiza Bunge [Lamiaceae; Salviae miltiorrhizae radix et rhizoma] | One dose per day, taken tiwce daily. The treatment observation period is 4 weeks. |
| LI SQ 2021 | Modified Gualou Xiebai Banxia Tang | *Synopsis of Golden Chamber* | Trichosanthis Fructus 25 g, Allii Macrostemonis Bulbus 12 g, Pinelliae Rhizoma 10 g, Carthami Flos 15 g, Puerariae Lobatae Radix 30 g, Curcumae Radix 10 g, Angelicae Sinensis Radix 15 g, Citri Reticulatae Pericarpium 15 g, Poria 15 g, Salviae Miltiorrhizae Radix et Rhizoma 15 g, Glycyrrhizae Radix et Rhizoma Praeparata 6 g | Trichosanthes kirilowii Maxim. [Cucurbitaceae]; Allium chinense G.Don [Amaryllidaceae]; Pinellia ternata (Thunb.) Makino [Araceae]; Carthamus tinctorius L. [Asteraceae]; Pueraria montana var. lobata (Willd.) Maesen & S.M.Almeida ex Sanjappa & Predeep [Fabaceae]; Curcuma longa L. [Zingiberaceae]; Neolitsea cassia (L.) Kosterm. [Lauraceae]; Citrus reticulata Blanco [Rutaceae]; Smilax glabra Roxb. [Smilacaceae]; Salvia miltiorrhiza Bunge [Lamiaceae; Salviae miltiorrhizae radix et rhizoma]; Glycyrrhiza uralensis Fisch. ex DC. [Fabaceae] | One dose per day, taken tiwce daily (100 ml per time). The treatment observation period is 4 weeks. |
| LI W 2018 | Modified Gualou Xiebai Banxia Tang | *Synopsis of Golden Chamber* | Trichosanthis Fructus 20 g, Allii Macrostemonis Bulbus 10 g, Typhae Pollen 10 g, Trogopterori Faeces 10 g, Pinelliae Rhizoma Praeparatum 10 g, Aurantii Fructus 10 g, Salviae Miltiorrhizae Radix et Rhizoma 20 g, Dalbergiae Odoriferae Lignum 10 g | Trichosanthes kirilowii Maxim. [Cucurbitaceae]; Allium chinense G.Don [Amaryllidaceae]; Typha angustifolia L. [Typhaceae]; Dried feces of Trogopterus xanthipes Milne-Edwards; Pinellia ternata (Thunb.) Makino [Araceae]; Citrus × aurantium f. aurantium [Rutaceae]; Salvia miltiorrhiza Bunge [Lamiaceae; Salviae miltiorrhizae radix et rhizoma]; Dalbergia odorifera T.C.Chen [Fabaceae] | One dose per day, taken tiwce daily (200 ml per time). The treatment observation period is 4 weeks. |
| LIU LH 2023 | Modified Gualou Xiebai Banxia Tang | *Synopsis of Golden Chamber* | Trichosanthis Fructus 20 g, Pinelliae Rhizoma 12 g, Agrimoniae Herba 12 g, Salviae Miltiorrhizae Radix et Rhizoma 16 g, Puerariae Lobatae Radix 15 g, Perillae Caulis 10 g, Aurantii Fructus 10 g, Allii Macrostemonis Bulbus 9 g, Notoginseng Radix et Rhizoma 6 g | Trichosanthes kirilowii Maxim. [Cucurbitaceae]; Pinellia ternata (Thunb.) Makino [Araceae]; Agrimonia pilosa Ledeb. [Rosaceae]; Salvia miltiorrhiza Bunge [Lamiaceae; Salviae miltiorrhizae radix et rhizoma]; Pueraria montana var. lobata (Willd.) Maesen & S.M.Almeida ex Sanjappa & Predeep [Fabaceae]; Perilla frutescens (L.) Britton [Lamiaceae]; Citrus × aurantium f. aurantium [Rutaceae]; Allium chinense G.Don [Amaryllidaceae]; Kalanchoe pinnata (Lam.) Pers. [Crassulaceae] | One dose per day, taken tiwce daily (150 ml per time). The treatment observation period is 4 weeks. |
| LIU YL 2020 | Modified Gualou Xiebai Banxia Tang | *Synopsis of Golden Chamber* | Trichosanthis Fructus 10 g, Arisaematis Cum Bile 10 g, Pinelliae Rhizoma 10 g, Allii Macrostemonis Bulbus 10 g, Poria 20 g, Codonopsis Radix 20 g, Bambusae Caulis in Taeniam 20 g, Citri Reticulatae Pericarpium 15 g, Acori Tatarinowii Rhizoma 15 g, Aurantii Fructus Immaturus 15 g, Glycyrrhizae Radix et Rhizoma 6 g | Trichosanthes kirilowii Maxim. [Cucurbitaceae]; Arisaema amurense Maxim. [Araceae]; Pinellia ternata (Thunb.) Makino [Araceae]; Allium chinense G.Don [Amaryllidaceae]; Smilax glabra Roxb. [Smilacaceae]; Codonopsis pilosula (Franch.) Nannf. [Campanulaceae]; Bambusa tuldoides Munro [Poaceae]; Citrus reticulata Blanco [Rutaceae]; Acorus gramineus Aiton [Acoraceae]; Citrus × aurantium f. aurantium [Rutaceae]; Glycyrrhiza uralensis Fisch. ex DC. [Fabaceae] | One dose per day, taken tiwce daily. The treatment observation period is 3 months. |
| LONG C 2019 | Modified Gualou Xiebai Banxia Tang | *Synopsis of Golden Chamber* | Trichosanthis Fructus 20 g, Allii Macrostemonis Bulbus 15 g, Pinelliae Rhizoma 12 g, Astragali Radix 30 g, Corydalis Rhizoma 12 g, Atractylodis Macrocephalae Rhizoma 9 g, Chuanxiong Rhizoma 9 g, Santali Albi Lignum 6 g, Vinum (Bai jiu) appropriate amount | Trichosanthes kirilowii Maxim. [Cucurbitaceae]; Allium chinense G.Don [Amaryllidaceae]; Pinellia ternata (Thunb.) Makino [Araceae]; Astragalus mongholicus Bunge [Fabaceae]; Corydalis yanhusuo (Y.H.Chou & Chun C.Hsu) W.T.Wang ex Z.Y.Su & C.Y.Wu [Papaveraceae]; Atractylodes macrocephala Koidz. [Asteraceae]; Conioselinum anthriscoides 'Chuanxiong' [Apiaceae]; Santalum album L. [Santalaceae]; Vinum | One dose per day, taken three times daily (100 ml per time). The treatment observation period is 4 weeks. |
| LU TX 2017 | Modified Gualou Xiebai Banxia Tang | *Synopsis of Golden Chamber* | Trichosanthis Fructus 15 g, Allii Macrostemonis Bulbus 20 g, Salviae Miltiorrhizae Radix et Rhizoma 15 g, Pinelliae Rhizoma Praeparatum 12 g, Persicae Semen 10 g, Carthami Flos 10 g, Citri Reticulatae Pericarpium 10 g, Poria 10 g, Notoginseng Radix et Rhizoma 6 g, Corydalis Rhizoma 10 g, Chuanxiong Rhizoma 10 g, Glycyrrhizae Radix et Rhizoma 6 g | Trichosanthes kirilowii Maxim. [Cucurbitaceae]; Allium chinense G.Don [Amaryllidaceae]; Salvia miltiorrhiza Bunge [Lamiaceae; Salviae miltiorrhizae radix et rhizoma]; Pinellia ternata (Thunb.) Makino [Araceae]; Juglans regia L. [Juglandaceae]; Carthamus tinctorius L. [Asteraceae]; Citrus reticulata Blanco [Rutaceae]; Smilax glabra Roxb. [Smilacaceae]; Kalanchoe pinnata (Lam.) Pers. [Crassulaceae]; Corydalis yanhusuo (Y.H.Chou & Chun C.Hsu) W.T.Wang ex Z.Y.Su & C.Y.Wu [Papaveraceae]; Conioselinum anthriscoides 'Chuanxiong' [Apiaceae]; Glycyrrhiza uralensis Fisch. ex DC. [Fabaceae] | One dose per day, taken tiwce (100 ml per time). The treatment observation period is 2 weeks. |
| MENG M 2012 | Modified Gualou Xiebai Banxia Tang | *Synopsis of Golden Chamber* | Trichosanthis Fructus 15 g, Allii Macrostemonis Bulbus 15 g, Pinelliae Rhizoma 15 g, Astragali Radix 20 g, Cinnamomi Ramulus 10 g, Notoginseng Radix et Rhizoma 10 g | Trichosanthes kirilowii Maxim. [Cucurbitaceae]; Allium chinense G.Don [Amaryllidaceae]; Pinellia ternata (Thunb.) Makino [Araceae]; Astragalus mongholicus Bunge [Fabaceae]; Angelica sinensis (Oliv.) Diels [Apiaceae]; Kalanchoe pinnata (Lam.) Pers. [Crassulaceae] | One dose per day, taken tiwce (200 ml per time). The treatment observation period is 4 weeks. |
| MI XS 2011 | Modified Gualou Xiebai Banxia Tang | *Synopsis of Golden Chamber* | Trichosanthis Fructus 10 g, Allii Macrostemonis Bulbus 10 g, Pinelliae Rhizoma Praeparatum 10 g, Salviae Miltiorrhizae Radix et Rhizoma 10 g, Carthami Flos 5 g, Chuanxiong Rhizoma 5 g, Crataegi Fructus 10 g | Trichosanthes kirilowii Maxim. [Cucurbitaceae]; Allium chinense G.Don [Amaryllidaceae]; Pinellia ternata (Thunb.) Makino [Araceae]; Salvia miltiorrhiza Bunge [Lamiaceae; Salviae miltiorrhizae radix et rhizoma]; Carthamus tinctorius L. [Asteraceae]; Conioselinum anthriscoides 'Chuanxiong' [Apiaceae]; Crataegus pinnatifida Bunge [Rosaceae] | One dose per day, taken tiwce. The treatment observation period is 4 weeks. |
| NIU YW 2022 | Modified Gualou Xiebai Banxia Tang | *Synopsis of Golden Chamber* | Astragali Radix 20 g, Paeoniae Radix Rubra 15 g, Pheretima 15 g, Chuanxiong Rhizoma 15 g, Persicae Semen 15 g, Trichosanthis Fructus 15 g, Allii Macrostemonis Bulbus 15 g, Pinelliae Rhizoma 15 g, Carthami Flos 10 g | Astragalus mongholicus Bunge [Fabaceae]; Paeonia veitchii Lynch [Paeoniaceae]; Pheretima; Conioselinum anthriscoides 'Chuanxiong' [Apiaceae]; Juglans regia L. [Juglandaceae]; Trichosanthes kirilowii Maxim. [Cucurbitaceae]; Allium chinense G.Don [Amaryllidaceae]; Pinellia ternata (Thunb.) Makino [Araceae]; Carthamus tinctorius L. [Asteraceae] | One dose per day, taken tiwce (150ml per time). The treatment observation period is 4 weeks. |
| SHU JB 2024 | Modified Gualou Xiebai Banxia Tang | *Synopsis of Golden Chamber* | Trichosanthis Fructus 24 g, Salviae Miltiorrhizae Radix et Rhizoma 15 g, Corydalis Rhizoma 15 g, Allii Macrostemonis Bulbus 10 g, Pinelliae Rhizoma 10 g, Aurantii Fructus 10 g, Angelicae Sinensis Radix 10 g, Citri Exocarpium Rubrum 10 g, Glycyrrhizae Radix et Rhizoma Praeparata 6 g, Notoginseng Radix et Rhizoma (powder) 3 g | Trichosanthes kirilowii Maxim. [Cucurbitaceae]; Salvia miltiorrhiza Bunge [Lamiaceae; Salviae miltiorrhizae radix et rhizoma]; Corydalis yanhusuo (Y.H.Chou & Chun C.Hsu) W.T.Wang ex Z.Y.Su & C.Y.Wu [Papaveraceae]; Allium chinense G.Don [Amaryllidaceae]; Pinellia ternata (Thunb.) Makino [Araceae]; Citrus × aurantium f. aurantium [Rutaceae]; Neolitsea cassia (L.) Kosterm. [Lauraceae]; Citrus reticulata Blanco [Rutaceae]; Glycyrrhiza uralensis Fisch. ex DC. [Fabaceae]; Kalanchoe pinnata (Lam.) Pers. [Crassulaceae] | One dose per day, taken tiwce (150ml per time). The treatment observation period is 4 weeks. |
| SU W 2016 | Modified Gualou Xiebai Banxia Tang | *Synopsis of Golden Chamber* | Trichosanthis Fructus 30 g, Allii Macrostemonis Bulbus 6 g, Pinelliae Rhizoma Praeparatum 12 g, Vinum (Huangjiu) 40 ml | Trichosanthes kirilowii Maxim. [Cucurbitaceae]; Allium chinense G.Don [Amaryllidaceae]; Pinellia ternata (Thunb.) Makino [Araceae]; Vinum | One dose per day, taken tiwce. The treatment observation period is 2 weeks. |
| TIAN DF 2016 | Modified Gualou Xiebai Banxia Tang | *Synopsis of Golden Chamber* | Trichosanthis Fructus 20 g, Pinelliae Rhizoma 12 g, Allii Macrostemonis Bulbus 9 g, Salviae Miltiorrhizae Radix et Rhizoma 16 g, Puerariae Lobatae Radix 15 g, Perillae Caulis 10 g, Aurantii Fructus 10 g, Notoginseng Radix et Rhizoma 6 g, Agrimoniae Herba 12 g, Forsythiae Fructus 12 g | Trichosanthes kirilowii Maxim. [Cucurbitaceae]; Pinellia ternata (Thunb.) Makino [Araceae]; Allium chinense G.Don [Amaryllidaceae]; Salvia miltiorrhiza Bunge [Lamiaceae; Salviae miltiorrhizae radix et rhizoma]; Pueraria montana var. lobata (Willd.) Maesen & S.M.Almeida ex Sanjappa & Predeep [Fabaceae]; Perilla frutescens (L.) Britton [Lamiaceae]; Citrus × aurantium f. aurantium [Rutaceae]; Kalanchoe pinnata (Lam.) Pers. [Crassulaceae]; Agrimonia pilosa Ledeb. [Rosaceae]; Hypericum perforatum L. [Hypericaceae] | One dose per day, taken tiwce (150ml per time). The treatment observation period is 4 weeks. |
| WANG AM 2024 | Modified Gualou Xiebai Banxia Tang | *Synopsis of Golden Chamber* | Astragali Radix 30 g, Trichosanthis Fructus 15 g, Allii Macrostemonis Bulbus 15 g, Poria 15 g, Atractylodis Macrocephalae Rhizoma 15 g, Salviae Miltiorrhizae Radix et Rhizoma 15 g, Pinelliae Rhizoma 15 g | Astragalus mongholicus Bunge [Fabaceae]; Trichosanthes kirilowii Maxim. [Cucurbitaceae]; Allium chinense G.Don [Amaryllidaceae]; Smilax glabra Roxb. [Smilacaceae]; Atractylodes macrocephala Koidz. [Asteraceae]; Salvia miltiorrhiza Bunge [Lamiaceae; Salviae miltiorrhizae radix et rhizoma]; Pinellia ternata (Thunb.) Makino [Araceae] | One dose per day, taken tiwce (150ml per time). The treatment observation period is 4 weeks. |
| WANG CG 2020 | Modified Gualou Xiebai Banxia Tang | *Synopsis of Golden Chamber* | Trichosanthis Fructus 15 g, Cinnamomi Ramulus 15 g, Poria 15 g, Allii Macrostemonis Bulbus 15 g, Pinelliae Rhizoma Praeparatum 10 g, Arisaematis Cum Bile 10 g, Glycyrrhizae Radix et Rhizoma 10 g, Aurantii Fructus Immaturus 10 g, Codonopsis Radix 10 g, Salviae Miltiorrhizae Radix et Rhizoma 10 g, Magnoliae Officinalis Cortex 10 g, Acori Tatarinowii Rhizoma 10 g | Trichosanthes kirilowii Maxim. [Cucurbitaceae]; Angelica sinensis (Oliv.) Diels [Apiaceae]; Smilax glabra Roxb. [Smilacaceae]; Allium chinense G.Don [Amaryllidaceae]; Pinellia ternata (Thunb.) Makino [Araceae]; Arisaema amurense Maxim. [Araceae]; Glycyrrhiza uralensis Fisch. ex DC. [Fabaceae]; Citrus × aurantium f. aurantium [Rutaceae]; Codonopsis pilosula (Franch.) Nannf. [Campanulaceae]; Salvia miltiorrhiza Bunge [Lamiaceae; Salviae miltiorrhizae radix et rhizoma]; Magnolia officinalis Rehder & E.H.Wilson [Magnoliaceae]; Acorus gramineus Aiton [Acoraceae] | One dose per day, taken tiwce. The treatment observation period is 4 weeks. |
| WANG EG 2017 | Modified Gualou Xiebai Banxia Tang | *Synopsis of Golden Chamber* | Acori Tatarinowii Rhizoma 6 g, Glycyrrhizae Radix et Rhizoma Praeparata 6 g, Citri Reticulatae Pericarpium 6 g, Rhodiolae Crenulatae Radix et Rhizoma 10 g, Aurantii Fructus Immaturus 10 g, Pinelliae Rhizoma Praeparatum 10 g, Poria 15 g, Chuanxiong Rhizoma 15 g, Allii Macrostemonis Bulbus 15 g, Trichosanthis Fructus 30 g | Acorus gramineus Aiton [Acoraceae]; Glycyrrhiza uralensis Fisch. ex DC. [Fabaceae]; Citrus reticulata Blanco [Rutaceae]; Rhodiola crenulata (Hook.f. & Thomson) H.Ohba [Crassulaceae]; Citrus × aurantium f. aurantium [Rutaceae]; Pinellia ternata (Thunb.) Makino [Araceae]; Smilax glabra Roxb. [Smilacaceae]; Conioselinum anthriscoides 'Chuanxiong' [Apiaceae]; Allium chinense G.Don [Amaryllidaceae]; Trichosanthes kirilowii Maxim. [Cucurbitaceae] | One dose per day, taken tiwce (150ml per time). The treatment observation period is 3 months. |
| WANG P 2021 | Modified Gualou Xiebai Banxia Tang | *Synopsis of Golden Chamber* | Trichosanthis Fructus 15 g, Allii Macrostemonis Bulbus 12 g, Pinelliae Rhizoma Praeparatum 12 g, Angelicae Sinensis Radix 12 g, Paeoniae Radix Rubra 12 g, Carthami Flos 10 g, Persicae Semen 10 g, Poria 10 g, Chuanxiong Rhizoma 10 g, Aurantii Fructus 6 g, Glycyrrhizae Radix et Rhizoma 3 g | Trichosanthes kirilowii Maxim. [Cucurbitaceae]; Allium chinense G.Don [Amaryllidaceae]; Pinellia ternata (Thunb.) Makino [Araceae]; Neolitsea cassia (L.) Kosterm. [Lauraceae]; Paeonia veitchii Lynch [Paeoniaceae]; Carthamus tinctorius L. [Asteraceae]; Juglans regia L. [Juglandaceae]; Smilax glabra Roxb. [Smilacaceae]; Conioselinum anthriscoides 'Chuanxiong' [Apiaceae]; Citrus × aurantium f. aurantium [Rutaceae]; Glycyrrhiza uralensis Fisch. ex DC. [Fabaceae] | One dose per day, taken tiwce. The treatment observation period is 30 days. |
| WANG SM 2021 | Modified Gualou Xiebai Banxia Tang | *Synopsis of Golden Chamber* | Trichosanthis Fructus 15 g, Allii Macrostemonis Bulbus 15 g, Pinelliae Rhizoma 15 g, Cinnamomi Ramulus 10 g, Poria 10 g, Codonopsis Radix 10 g, Glycyrrhizae Radix et Rhizoma 6 g | Trichosanthes kirilowii Maxim. [Cucurbitaceae]; Allium chinense G.Don [Amaryllidaceae]; Pinellia ternata (Thunb.) Makino [Araceae]; Angelica sinensis (Oliv.) Diels [Apiaceae]; Smilax glabra Roxb. [Smilacaceae]; Codonopsis pilosula (Franch.) Nannf. [Campanulaceae]; Glycyrrhiza uralensis Fisch. ex DC. [Fabaceae] | One dose per day, taken three times daily. The treatment observation period is 4 weeks. |
| WANG X 2019 | Modified Gualou Xiebai Banxia Tang | *Synopsis of Golden Chamber* | Trichosanthis Fructus 15 g, Allii Macrostemonis Bulbus 10 g, Pinelliae Rhizoma Praeparatum 10 g, Cinnamomi Ramulus 10 g, Salviae Miltiorrhizae Radix et Rhizoma 30 g | Trichosanthes kirilowii Maxim. [Cucurbitaceae]; Allium chinense G.Don [Amaryllidaceae]; Pinellia ternata (Thunb.) Makino [Araceae]; Angelica sinensis (Oliv.) Diels [Apiaceae]; Salvia miltiorrhiza Bunge [Lamiaceae; Salviae miltiorrhizae radix et rhizoma] | One dose per day, taken tiwce (100ml per time). The treatment observation period is 1 month. |
| WEI ZH 2024 | Modified Gualou Xiebai Banxia Tang | *Synopsis of Golden Chamber* | Citri Reticulatae Pericarpium 6 g, Glycyrrhizae Radix et Rhizoma Praeparata 6 g, Acori Tatarinowii Rhizoma 6 g, Rhodiolae Crenulatae Radix et Rhizoma 10 g, Pinelliae Rhizoma Praeparatum 10 g, Aurantii Fructus Immaturus 10 g, Allii Macrostemonis Bulbus 15 g, Chuanxiong Rhizoma 15 g, Poria 15 g, Trichosanthis Fructus 30 g | Citrus reticulata Blanco [Rutaceae]; Glycyrrhiza uralensis Fisch. ex DC. [Fabaceae]; Acorus gramineus Aiton [Acoraceae]; Rhodiola crenulata (Hook.f. & Thomson) H.Ohba [Crassulaceae]; Pinellia ternata (Thunb.) Makino [Araceae]; Citrus × aurantium f. aurantium [Rutaceae]; Allium chinense G.Don [Amaryllidaceae]; Conioselinum anthriscoides 'Chuanxiong' [Apiaceae]; Smilax glabra Roxb. [Smilacaceae]; Trichosanthes kirilowii Maxim. [Cucurbitaceae] | One dose per day, taken tiwce (150ml per time). The treatment observation period is 3 months. |
| WEI M 2009 | Modified Gualou Xiebai Banxia Tang | *Synopsis of Golden Chamber* | Not stated |  | One dose per day, taken three times daily. The treatment observation period is 2 weeks. |
| WU T 2023 | Modified Gualou Xiebai Banxia Tang | *Synopsis of Golden Chamber* | Trichosanthis Fructus 15 g, Allii Macrostemonis Bulbus 15 g, Pinelliae Rhizoma Praeparatum 10 g, Acori Tatarinowii Rhizoma 20 g, Codonopsis Radix 20 g, Poria 15 g, Atractylodis Macrocephalae Rhizoma 15 g, Citri Reticulatae Pericarpium 15 g, Magnoliae Officinalis Cortex 10 g, Aurantii Fructus Immaturus 10 g, Curcumae Radix 10 g, Salviae Miltiorrhizae Radix et Rhizoma 20 g, Glycyrrhizae Radix et Rhizoma 5 g | Trichosanthes kirilowii Maxim. [Cucurbitaceae]; Allium chinense G.Don [Amaryllidaceae]; Pinellia ternata (Thunb.) Makino [Araceae]; Acorus gramineus Aiton [Acoraceae]; Codonopsis pilosula (Franch.) Nannf. [Campanulaceae]; Smilax glabra Roxb. [Smilacaceae]; Atractylodes macrocephala Koidz. [Asteraceae]; Citrus reticulata Blanco [Rutaceae]; Magnolia officinalis Rehder & E.H.Wilson [Magnoliaceae]; Citrus × aurantium f. aurantium [Rutaceae]; Curcuma longa L. [Zingiberaceae]; Salvia miltiorrhiza Bunge [Lamiaceae; Salviae miltiorrhizae radix et rhizoma]; Glycyrrhiza uralensis Fisch. ex DC. [Fabaceae] | One dose per day, taken tiwce. The treatment observation period is 4 weeks. |
| XV J 2016 | Modified Gualou Xiebai Banxia Tang | *Synopsis of Golden Chamber* | Trichosanthis Fructus 30 g, Allii Macrostemonis Bulbus 15 g, Chuanxiong Rhizoma 15 g, Poria 15 g, Pinelliae Rhizoma Praeparatum 10 g, Aurantii Fructus Immaturus 10 g, Rhodiolae Crenulatae Radix et Rhizoma 10 g, Citri Reticulatae Pericarpium 6 g, Glycyrrhizae Radix et Rhizoma Praeparata 6 g, Acori Tatarinowii Rhizoma 6 g | Trichosanthes kirilowii Maxim. [Cucurbitaceae]; Allium chinense G.Don [Amaryllidaceae]; Conioselinum anthriscoides 'Chuanxiong' [Apiaceae]; Smilax glabra Roxb. [Smilacaceae]; Pinellia ternata (Thunb.) Makino [Araceae]; Citrus × aurantium f. aurantium [Rutaceae]; Rhodiola crenulata (Hook.f. & Thomson) H.Ohba [Crassulaceae]; Citrus reticulata Blanco [Rutaceae]; Glycyrrhiza uralensis Fisch. ex DC. [Fabaceae]; Acorus gramineus Aiton [Acoraceae] | One dose per day, taken tiwce (150ml per time). The treatment observation period is 90 days. |
| YAN XS 2018 | Modified Gualou Xiebai Banxia Tang | *Synopsis of Golden Chamber* | Trichosanthis Fructus 12 g, Allii Macrostemonis Bulbus 9 g, Pinelliae Rhizoma 9 g, Vinum (Huangjiu) 50 mL | Trichosanthes kirilowii Maxim. [Cucurbitaceae]; Allium chinense G.Don [Amaryllidaceae]; Pinellia ternata (Thunb.) Makino [Araceae]; Vinum | One dose per day, taken tiwce (200ml per time). The treatment observation period is 4 weeks. |
| YAN JG 2023 | Modified Gualou Xiebai Banxia Tang | *Synopsis of Golden Chamber* | Trichosanthis Fructus 12 g, Citri Reticulatae Pericarpium 12 g, Pinelliae Rhizoma 12 g, Poria 12 g, Allii Macrostemonis Bulbus 9 g | Trichosanthes kirilowii Maxim. [Cucurbitaceae]; Citrus reticulata Blanco [Rutaceae]; Pinellia ternata (Thunb.) Makino [Araceae]; Smilax glabra Roxb. [Smilacaceae]; Allium chinense G.Don [Amaryllidaceae] | One dose per day, taken three times daily (150ml per time). The treatment observation period is 2 months. |
| YANG FQ 2015 | Modified Gualou Xiebai Banxia Tang | *Synopsis of Golden Chamber* | Trichosanthis Fructus 30 g, Allii Macrostemonis Bulbus 15 g, Pinelliae Rhizoma Praeparatum 10 g, Puerariae Lobatae Radix 25 g, Paeoniae Radix Alba 20 g, Salviae Miltiorrhizae Radix et Rhizoma 15 g, Poria 15 g, Bambusae Caulis in Taeniam 12 g, Codonopsis Radix 10 g, Cinnamomi Ramulus 10 g, Curcumae Radix 10 g, Bupleuri Radix 10 g, Corydalis Rhizoma 10 g, Citri Reticulatae Pericarpium 9 g | Trichosanthes kirilowii Maxim. [Cucurbitaceae]; Allium chinense G.Don [Amaryllidaceae]; Pinellia ternata (Thunb.) Makino [Araceae]; Pueraria montana var. lobata (Willd.) Maesen & S.M.Almeida ex Sanjappa & Predeep [Fabaceae]; Cynanchum otophyllum C.K.Schneid. [Apocynaceae]; Salvia miltiorrhiza Bunge [Lamiaceae; Salviae miltiorrhizae radix et rhizoma]; Smilax glabra Roxb. [Smilacaceae]; Bambusa tuldoides Munro [Poaceae]; Codonopsis pilosula (Franch.) Nannf. [Campanulaceae]; Angelica sinensis (Oliv.) Diels [Apiaceae]; Curcuma longa L. [Zingiberaceae]; Elephantopus scaber L. [Asteraceae]; Corydalis yanhusuo (Y.H.Chou & Chun C.Hsu) W.T.Wang ex Z.Y.Su & C.Y.Wu [Papaveraceae]; Citrus reticulata Blanco [Rutaceae] | One dose per day, taken tiwce daily (150ml per time). The treatment observation period is 2 months. |
| YANG JK 2015 | Modified Gualou Xiebai Banxia Tang | *Synopsis of Golden Chamber* | Trichosanthis Fructus 15 g, Aurantii Fructus 15 g, Allii Macrostemonis Bulbus 15 g, Pinelliae Rhizoma Praeparatum 10 g, Dalbergiae Odoriferae Lignum 10 g, Chuanxiong Rhizoma 10 g, Salviae Miltiorrhizae Radix et Rhizoma 10 g, Paeoniae Radix Rubra 10 g, Carthami Flos 10 g, Poria 10 g | Trichosanthes kirilowii Maxim. [Cucurbitaceae]; Citrus × aurantium f. aurantium [Rutaceae]; Allium chinense G.Don [Amaryllidaceae]; Pinellia ternata (Thunb.) Makino [Araceae]; Dalbergia odorifera T.C.Chen [Fabaceae]; Conioselinum anthriscoides 'Chuanxiong' [Apiaceae]; Salvia miltiorrhiza Bunge [Lamiaceae; Salviae miltiorrhizae radix et rhizoma]; Paeonia veitchii Lynch [Paeoniaceae]; Carthamus tinctorius L. [Asteraceae]; Smilax glabra Roxb. [Smilacaceae] | One dose per day, taken tiwce daily (150ml per time). The treatment observation period is 4 weeks. |
| YANG QF 2015 | Modified Gualou Xiebai Banxia Tang | *Synopsis of Golden Chamber* | Trichosanthis Fructus 25 g, Allii Macrostemonis Bulbus 15 g, Pinelliae Rhizoma 10 g, Codonopsis Radix 10 g, Cinnamomi Ramulus 10 g, Curcumae Radix 10 g, Persicae Semen 10 g, Carthami Flos 20 g, Corydalis Rhizoma 10 g, Astragali Radix 20 g | Trichosanthes kirilowii Maxim. [Cucurbitaceae]; Allium chinense G.Don [Amaryllidaceae]; Pinellia ternata (Thunb.) Makino [Araceae]; Codonopsis pilosula (Franch.) Nannf. [Campanulaceae]; Angelica sinensis (Oliv.) Diels [Apiaceae]; Curcuma longa L. [Zingiberaceae]; Juglans regia L. [Juglandaceae]; Carthamus tinctorius L. [Asteraceae]; Corydalis yanhusuo (Y.H.Chou & Chun C.Hsu) W.T.Wang ex Z.Y.Su & C.Y.Wu [Papaveraceae]; Astragalus mongholicus Bunge [Fabaceae] | One dose per day, taken tiwce daily. The treatment observation period is 2 weeks. |
| YANG XY 2007 | Modified Gualou Xiebai Banxia Tang | *Synopsis of Golden Chamber* | Allii Macrostemonis Bulbus 10 g, Trichosanthis Fructus 10 g, Pinelliae Rhizoma Praeparatum 10 g, Rhodiolae Crenulatae Radix et Rhizoma 10 g, Aurantii Fructus Immaturus 10 g, Curcumae Radix 10 g, Poria 15 g, Chuanxiong Rhizoma 15 g, Citri Reticulatae Pericarpium 6 g, Acori Tatarinowii Rhizoma 6 g, Glycyrrhizae Radix et Rhizoma Praeparata 6 g | Allium chinense G.Don [Amaryllidaceae]; Trichosanthes kirilowii Maxim. [Cucurbitaceae]; Pinellia ternata (Thunb.) Makino [Araceae]; Rhodiola crenulata (Hook.f. & Thomson) H.Ohba [Crassulaceae]; Citrus × aurantium f. aurantium [Rutaceae]; Curcuma longa L. [Zingiberaceae]; Smilax glabra Roxb. [Smilacaceae]; Conioselinum anthriscoides 'Chuanxiong' [Apiaceae]; Citrus reticulata Blanco [Rutaceae]; Acorus gramineus Aiton [Acoraceae]; Glycyrrhiza uralensis Fisch. ex DC. [Fabaceae] | One dose per day, taken tiwce daily (150ml per time). The treatment observation period is 4 weeks. |
| YANG X 2023 | Modified Gualou Xiebai Banxia Tang | *Synopsis of Golden Chamber* | Trichosanthis Fructus 30 g, Allii Macrostemonis Bulbus 15 g, Salviae Miltiorrhizae Radix et Rhizoma 15 g, Pinelliae Rhizoma Praeparatum 10 g, Hirudo (powder) 3 g, Monasci Purpurei 6 g, Crataegi Fructus Crudum 10 g, Dalbergiae Odoriferae Lignum 10 g, Astragali Radix 30 g, Chuanxiong Rhizoma 10 g, Poria 10 g, Glycyrrhizae Radix et Rhizoma 6 g | Trichosanthes kirilowii Maxim. [Cucurbitaceae]; Allium chinense G.Don [Amaryllidaceae]; Salvia miltiorrhiza Bunge [Lamiaceae; Salviae miltiorrhizae radix et rhizoma]; Pinellia ternata (Thunb.) Makino [Araceae]; Whitmania pigra Whitman; Monascus purpureus; Crataegus pinnatifida Bunge [Rosaceae]; Dalbergia odorifera T.C.Chen [Fabaceae]; Astragalus mongholicus Bunge [Fabaceae]; Conioselinum anthriscoides 'Chuanxiong' [Apiaceae]; Smilax glabra Roxb. [Smilacaceae]; Glycyrrhiza uralensis Fisch. ex DC. [Fabaceae] | One dose per day, taken tiwce daily (200ml per time). The treatment observation period is 4 weeks. |
| ZHANG XF 2024 | Modified Gualou Xiebai Banxia Tang | *Synopsis of Golden Chamber* | Salviae Miltiorrhizae Radix et Rhizoma 20 g, Trichosanthis Fructus 20 g, Pinelliae Rhizoma Praeparatum 15 g, Carthami Flos 12 g, Achyranthis Bidentatae Radix 20 g, Ziziphi Spinosae Semen 15 g, Allii Macrostemonis Bulbus 12 g, Paeoniae Radix Rubra 12 g, Dalbergiae Odoriferae Lignum 5 g, Taxilli Herba 20 g, Eucommiae Cortex Praeparata 20 g, Astragali Radix 30 g, Chuanxiong Rhizoma 12 g | Salvia miltiorrhiza Bunge [Lamiaceae; Salviae miltiorrhizae radix et rhizoma]; Trichosanthes kirilowii Maxim. [Cucurbitaceae]; Pinellia ternata (Thunb.) Makino [Araceae]; Carthamus tinctorius L. [Asteraceae]; Achyranthes bidentata Blume [Amaranthaceae]; Ziziphus jujuba subsp. spinosa (Bunge) J.Y.Peng, X.Y.Li & L.Li [Rhamnaceae]; Allium chinense G.Don [Amaryllidaceae]; Paeonia veitchii Lynch [Paeoniaceae]; Dalbergia odorifera T.C.Chen [Fabaceae]; Taxillus chinensis (DC.) Danser; Eucommia ulmoides Oliv. [Eucommiaceae]; Astragalus mongholicus Bunge [Fabaceae]; Conioselinum anthriscoides 'Chuanxiong' [Apiaceae] | One dose per day, taken tiwce daily (200ml per time). The treatment observation period is 10 days. |
| ZHANG ZF 2016 | Modified Gualou Xiebai Banxia Tang | *Synopsis of Golden Chamber* | Trichosanthis Fructus 25 g, Cinnamomi Ramulus 8 g, Persicae Semen 10 g, Salviae Miltiorrhizae Radix et Rhizoma 25 g, Pinelliae Rhizoma Praeparatum 8 g, Allii Macrostemonis Bulbus 20 g, Aurantii Fructus Immaturus 10 g, Acori Tatarinowii Rhizoma 10 g, Paeoniae Radix Rubra 10 g, Curcumae Radix 10 g, Dalbergiae Odoriferae Lignum 10 g | Trichosanthes kirilowii Maxim. [Cucurbitaceae]; Angelica sinensis (Oliv.) Diels [Apiaceae]; Juglans regia L. [Juglandaceae]; Salvia miltiorrhiza Bunge [Lamiaceae; Salviae miltiorrhizae radix et rhizoma]; Pinellia ternata (Thunb.) Makino [Araceae]; Allium chinense G.Don [Amaryllidaceae]; Citrus × aurantium f. aurantium [Rutaceae]; Acorus gramineus Aiton [Acoraceae]; Paeonia veitchii Lynch [Paeoniaceae]; Curcuma longa L. [Zingiberaceae]; Dalbergia odorifera T.C.Chen [Fabaceae] | One dose per day, taken tiwce daily. The treatment observation period is 30 days. |
| ZHAO HY 2024 | Modified Gualou Xiebai Banxia Tang | *Synopsis of Golden Chamber* | Trichosanthis Fructus 15 g, Pinelliae Rhizoma 15 g, Allii Macrostemonis Bulbus 15 g, Astragali Radix 15 g, Salviae Miltiorrhizae Radix et Rhizoma 15 g, Notoginseng Radix et Rhizoma 12 g, Citri Reticulatae Pericarpium 10 g, Aurantii Fructus Immaturus 10 g, Atractylodis Macrocephalae Rhizoma 10 g, Santali Albi Lignum 10 g, Angelicae Sinensis Radix 10 g, Glycyrrhizae Radix et Rhizoma 6 g | Trichosanthes kirilowii Maxim. [Cucurbitaceae]; Pinellia ternata (Thunb.) Makino [Araceae]; Allium chinense G.Don [Amaryllidaceae]; Astragalus mongholicus Bunge [Fabaceae]; Salvia miltiorrhiza Bunge [Lamiaceae; Salviae miltiorrhizae radix et rhizoma]; Kalanchoe pinnata (Lam.) Pers. [Crassulaceae]; Citrus reticulata Blanco [Rutaceae]; Citrus × aurantium f. aurantium [Rutaceae]; Atractylodes macrocephala Koidz. [Asteraceae]; Santalum album L. [Santalaceae]; Neolitsea cassia (L.) Kosterm. [Lauraceae]; Glycyrrhiza uralensis Fisch. ex DC. [Fabaceae] | One dose per day, taken tiwce daily (250ml per time). The treatment observation period is 2 months. |
| ZHAO YL 2013 | Modified Gualou Xiebai Banxia Tang | *Synopsis of Golden Chamber* | Trichosanthis Fructus 15 g, Allii Macrostemonis Bulbus 10 g, Pinelliae Rhizoma 15 g, Salviae Miltiorrhizae Radix et Rhizoma 20 g, Puerariae Lobatae Radix 20 g | Trichosanthes kirilowii Maxim. [Cucurbitaceae]; Allium chinense G.Don [Amaryllidaceae]; Pinellia ternata (Thunb.) Makino [Araceae]; Salvia miltiorrhiza Bunge [Lamiaceae; Salviae miltiorrhizae radix et rhizoma]; Pueraria montana var. lobata (Willd.) Maesen & S.M.Almeida ex Sanjappa & Predeep [Fabaceae] | One dose per day, taken tiwce daily (150ml per time). The treatment observation period is 4 weeks. |
| ZHAO YG 2024 | Modified Gualou Xiebai Banxia Tang | *Synopsis of Golden Chamber* | Trichosanthis Fructus 30 g, Rhodiolae Crenulatae Radix et Rhizoma 10 g, Pinelliae Rhizoma Praeparatum 10 g, Curcumae Radix 10 g, Aurantii Fructus Immaturus 10 g, Chuanxiong Rhizoma 15 g, Poria 15 g, Allii Macrostemonis Bulbus 15 g, Citri Reticulatae Pericarpium 6 g, Acori Tatarinowii Rhizoma 6 g, Glycyrrhizae Radix et Rhizoma Praeparata 6 g | Trichosanthes kirilowii Maxim. [Cucurbitaceae]; Rhodiola crenulata (Hook.f. & Thomson) H.Ohba [Crassulaceae]; Pinellia ternata (Thunb.) Makino [Araceae]; Curcuma longa L. [Zingiberaceae]; Citrus × aurantium f. aurantium [Rutaceae]; Conioselinum anthriscoides 'Chuanxiong' [Apiaceae]; Smilax glabra Roxb. [Smilacaceae]; Allium chinense G.Don [Amaryllidaceae]; Citrus reticulata Blanco [Rutaceae]; Acorus gramineus Aiton [Acoraceae]; Glycyrrhiza uralensis Fisch. ex DC. [Fabaceae] | One dose per day, taken tiwce daily (150ml per time). The treatment observation period is 1 months. |
| ZHENG XB 2020 | Modified Gualou Xiebai Banxia Tang | *Synopsis of Golden Chamber* | Trichosanthis Fructus 30 g, Allii Macrostemonis Bulbus 12 g, Pinelliae Rhizoma Praeparatum 12 g | Trichosanthes kirilowii Maxim. [Cucurbitaceae]; Allium chinense G.Don [Amaryllidaceae]; Pinellia ternata (Thunb.) Makino [Araceae] | One dose per day, taken tiwce daily (150-200ml per time). The treatment observation period is 8 weeks. |
| ZHOU HT 2006 | Modified Gualou Xiebai Banxia Tang | *Synopsis of Golden Chamber* | Trichosanthis Fructus 15 g, Allii Macrostemonis Bulbus 10 g, Pinelliae Rhizoma Praeparatum 10 g, Cinnamomi Ramulus 6 g, Salviae Miltiorrhizae Radix et Rhizoma 15 g, Glycyrrhizae Radix et Rhizoma 6 g, Ginseng Radix et Rhizoma Alba 10 g, Bombyx Batryticatus 10 g | Trichosanthes kirilowii Maxim. [Cucurbitaceae]; Allium chinense G.Don [Amaryllidaceae]; Pinellia ternata (Thunb.) Makino [Araceae]; Angelica sinensis (Oliv.) Diels [Apiaceae]; Salvia miltiorrhiza Bunge [Lamiaceae; Salviae miltiorrhizae radix et rhizoma]; Glycyrrhiza uralensis Fisch. ex DC. [Fabaceae]; Panax ginseng C.A.Mey. [Araliaceae]; Bombyx batryticatus | One dose per day, taken tiwce daily (200ml per time). The treatment observation period is 2 months. |
| ZHU DJ 2021 | Modified Gualou Xiebai Banxia Tang | *Synopsis of Golden Chamber* | Trichosanthis Fructus 24 g, Allii Macrostemonis Bulbus 9 g, Pinelliae Rhizoma Praeparatum 12 g, Vinum (Mijiu) 100 mL | Trichosanthes kirilowii Maxim. [Cucurbitaceae]; Allium chinense G.Don [Amaryllidaceae]; Pinellia ternata (Thunb.) Makino [Araceae]; Vinum | One dose per day, taken tiwce daily (150ml per time). The treatment observation period is 2 weeks. |
| ZHU ZY 2019 | Modified Gualou Xiebai Banxia Tang | *Synopsis of Golden Chamber* | Trichosanthis Fructus 20 g, Allii Macrostemonis Bulbus 10 g, Pinelliae Rhizoma 10 g, Salviae Miltiorrhizae Radix et Rhizoma 15 g, Corydalis Rhizoma 10 g, Angelicae Sinensis Radix 10 g, Citri Exocarpium Rubrum 10 g, Aurantii Fructus 10 g, Glycyrrhizae Radix et Rhizoma Praeparata 6 g, Notoginseng Radix et Rhizoma (powder, taken with water) 3 g | Trichosanthes kirilowii Maxim. [Cucurbitaceae]; Allium chinense G.Don [Amaryllidaceae]; Pinellia ternata (Thunb.) Makino [Araceae]; Salvia miltiorrhiza Bunge [Lamiaceae; Salviae miltiorrhizae radix et rhizoma]; Corydalis yanhusuo (Y.H.Chou & Chun C.Hsu) W.T.Wang ex Z.Y.Su & C.Y.Wu [Papaveraceae]; Neolitsea cassia (L.) Kosterm. [Lauraceae]; Citrus reticulata Blanco [Rutaceae]; Citrus × aurantium f. aurantium [Rutaceae]; Glycyrrhiza uralensis Fisch. ex DC. [Fabaceae]; Kalanchoe pinnata (Lam.) Pers. [Crassulaceae] | One dose per day, taken tiwce daily. The treatment observation period is 4 weeks. |
| ZHUANG HB 2020 | Modified Gualou Xiebai Banxia Tang | *Synopsis of Golden Chamber* | Trichosanthis Fructus 30 g, Allii Macrostemonis Bulbus 15 g, Pinelliae Rhizoma Praeparatum 12 g, Poria 30 g, Coicis Semen 30 g, Aconiti Lateralis Radix Praeparata 15 g, Cinnamomi Ramulus 9 g, Astragali Radix 30 g, Dioscoreae Rhizoma 15 g, Glycyrrhizae Radix et Rhizoma Praeparata 5 g | Trichosanthes kirilowii Maxim. [Cucurbitaceae]; Allium chinense G.Don [Amaryllidaceae]; Pinellia ternata (Thunb.) Makino [Araceae]; Smilax glabra Roxb. [Smilacaceae]; Coix lacryma-jobi L. [Poaceae]; Aconitum carmichaelii Debeaux (prepared); Angelica sinensis (Oliv.) Diels [Apiaceae]; Astragalus mongholicus Bunge [Fabaceae]; Dioscorea oppositifolia L. [Dioscoreaceae]; Glycyrrhiza uralensis Fisch. ex DC. [Fabaceae] | One dose per day, taken tiwce daily (200ml per time). The treatment observation period is 1 months. |
| HUANG PY 2008 | Modified Guizhi Fuling Wan/Tang | *Synopsis of Golden Chamber* | Cinnamomi Ramulus 15 g, Poria 20 g, Persicae Semen 15 g, Moutan Cortex 15 g, Paeoniae Radix Alba 15 g, Allii Macrostemonis Bulbus 15 g, Pinelliae Rhizoma Praeparatum 12 g | Angelica sinensis (Oliv.) Diels [Apiaceae]; Smilax glabra Roxb. [Smilacaceae]; Juglans regia L. [Juglandaceae]; Paeonia × suffruticosa Andrews [Paeoniaceae]; Cynanchum otophyllum C.K.Schneid. [Apocynaceae]; Allium chinense G.Don [Amaryllidaceae]; Pinellia ternata (Thunb.) Makino [Araceae] | One dose per day, taken tiwce daily (125ml per time). The treatment observation period is 4 weeks. |
| LU XY 2012 | Modified Guizhi Fuling Wan/Tang | *Synopsis of Golden Chamber* | Cinnamomi Ramulus 15 g, Poria 20 g, Moutan Cortex 12 g, Paeoniae Radix Alba 12 g, Persicae Semen 10 g, Carthami Flos 6 g, Chuanxiong Rhizoma 15 g, Angelicae Sinensis Radix 15 g, Glycyrrhizae Radix et Rhizoma Praeparata 10 g | Angelica sinensis (Oliv.) Diels [Apiaceae]; Smilax glabra Roxb. [Smilacaceae]; Paeonia × suffruticosa Andrews [Paeoniaceae]; Cynanchum otophyllum C.K.Schneid. [Apocynaceae]; Juglans regia L. [Juglandaceae]; Carthamus tinctorius L. [Asteraceae]; Conioselinum anthriscoides 'Chuanxiong' [Apiaceae]; Neolitsea cassia (L.) Kosterm. [Lauraceae]; Glycyrrhiza uralensis Fisch. ex DC. [Fabaceae] | One dose per day, taken tiwce daily. The treatment observation period is 4 weeks. |
| PEI Q 2013 | Modified Guizhi Fuling Wan/Tang | *Synopsis of Golden Chamber* | Cinnamomi Ramulus 12 g, Poria 20 g, Moutan Cortex 10 g, Persicae Semen 10 g, Paeoniae Radix Alba 10 g | Angelica sinensis (Oliv.) Diels [Apiaceae]; Smilax glabra Roxb. [Smilacaceae]; Paeonia × suffruticosa Andrews [Paeoniaceae]; Juglans regia L. [Juglandaceae]; Cynanchum otophyllum C.K.Schneid. [Apocynaceae] | One dose per day, taken tiwce daily (100ml per time). The treatment observation period is 4 weeks. |
| XV GY 2017 | Modified Guizhi Fuling Wan/Tang | *Synopsis of Golden Chamber* | Moutan Cortex 20 g, Poria 20 g, Paeoniae Radix Rubra 20 g, Persicae Semen 15 g, Cinnamomi Ramulus 15 g | Paeonia × suffruticosa Andrews [Paeoniaceae]; Smilax glabra Roxb. [Smilacaceae]; Paeonia veitchii Lynch [Paeoniaceae]; Juglans regia L. [Juglandaceae]; Angelica sinensis (Oliv.) Diels [Apiaceae] | One dose per day, taken tiwce daily. The treatment observation period is 1 months. |
| XV Y 2023 | Modified Guizhi Fuling Wan/Tang | *Synopsis of Golden Chamber* | Cinnamomi Ramulus 15 g, Poria 15 g, Paeoniae Radix Alba 15 g, Persicae Semen 10 g, Moutan Cortex 10 g, Aucklandiae Radix 10 g, Bupleuri Radix 15 g, Toosendan Fructus 10 g | Angelica sinensis (Oliv.) Diels [Apiaceae]; Smilax glabra Roxb. [Smilacaceae]; Cynanchum otophyllum C.K.Schneid. [Apocynaceae]; Juglans regia L. [Juglandaceae]; Paeonia × suffruticosa Andrews [Paeoniaceae]; Dolomiaea costus (Falc.) Kasana & A.K.Pandey [Asteraceae]; Elephantopus scaber L. [Asteraceae]; Toosendan Fructus (Melia toosendan) | One dose per day, taken tiwce daily (150ml per time). The treatment observation period is 4 weeks. |
| YU P 2014 | Modified Guizhi Fuling Wan/Tang | *Synopsis of Golden Chamber* | Moutan Cortex 20 g, Paeoniae Radix Rubra 20 g, Persicae Semen 15 g, Cinnamomi Ramulus 15 g, Poria 20 g | Paeonia × suffruticosa Andrews [Paeoniaceae]; Paeonia veitchii Lynch [Paeoniaceae]; Juglans regia L. [Juglandaceae]; Angelica sinensis (Oliv.) Diels [Apiaceae]; Smilax glabra Roxb. [Smilacaceae] | One dose per day, taken tiwce daily. The treatment observation period is 4 weeks. |
| ZHENG WD 2015 | Modified Guizhi Fuling Wan/Tang | *Synopsis of Golden Chamber* | Guizhi Fuling Capsules (Jiangsu Kanion Pharmaceutical Co., Ltd., National Drug Approval Number: Z10950005, specification: 0.31 g × 100 capsules) | Guizhi Fuling Capsules (Jiangsu Kanion Pharmaceutical Co., Ltd., National Drug Approval Number: Z10950005, specification: 0.31 g × 100 capsules) | Administered orally at a dose of 0.93 g per time, three times a day. The treatment observation period is 4 weeks. |
| ZHONG F 2022 | Modified Guizhi Fuling Wan/Tang | *Synopsis of Golden Chamber* | Guizhi Fuling Capsules (Jiangsu Kanion Pharmaceutical Co., Ltd., National Drug Approval Number: Z10950005, specification: 0.31 g × 100 capsules) | Guizhi Fuling Capsules (Jiangsu Kanion Pharmaceutical Co., Ltd., National Drug Approval Number: Z10950005, specification: 0.31 g × 100 capsules) | Administered orally at a dose of 0.93 g per time, three times a day. The treatment observation period is 3 months. |
| ZHOU WR 2017 | Modified Guizhi Fuling Wan/Tang | *Synopsis of Golden Chamber* | Cinnamomi Ramulus 9 g, Poria 15 g, Paeoniae Radix Alba 15 g, Persicae Semen 9 g | Angelica sinensis (Oliv.) Diels [Apiaceae]; Smilax glabra Roxb. [Smilacaceae]; Cynanchum otophyllum C.K.Schneid. [Apocynaceae]; Juglans regia L. [Juglandaceae] | One dose per day, taken tiwce daily. The treatment observation period is 4 weeks. |
| AN LJ 2017 | Modified Ling Gui Zhu Gan Tang | *Synopsis of Golden Chamber* | Poria 20 g, Cinnamomi Ramulus 15 g, Atractylodis Macrocephalae Rhizoma 15 g, Glycyrrhizae Radix et Rhizoma Praeparata 10 g, Salviae Miltiorrhizae Radix et Rhizoma 15 g, Acori Tatarinowii Rhizoma 10 g | Smilax glabra Roxb. [Smilacaceae]; Angelica sinensis (Oliv.) Diels [Apiaceae]; Atractylodes macrocephala Koidz. [Asteraceae]; Glycyrrhiza uralensis Fisch. ex DC. [Fabaceae]; Salvia miltiorrhiza Bunge [Lamiaceae; Salviae miltiorrhizae radix et rhizoma]; Acorus gramineus Aiton [Acoraceae] | One dose per day, taken tiwce daily. The treatment observation period is 4 weeks. |
| DU LY 2019 | Modified Ling Gui Zhu Gan Tang | *Synopsis of Golden Chamber* | Poria 12 g, Cinnamomi Ramulus 9 g, Atractylodis Macrocephalae Rhizoma 6 g, Glycyrrhizae Radix et Rhizoma 6 g | Smilax glabra Roxb. [Smilacaceae]; Angelica sinensis (Oliv.) Diels [Apiaceae]; Atractylodes macrocephala Koidz. [Asteraceae]; Glycyrrhiza uralensis Fisch. ex DC. [Fabaceae] | One dose per day, taken tiwce daily. The treatment observation period is 1 month. |
| HUA MZ 2023 | Modified Ling Gui Zhu Gan Tang | *Synopsis of Golden Chamber* | Poria 20 g, Alismatis Rhizoma 20 g, Polygonati Odorati Rhizoma 20 g, Lepidii Semen 15 g, Atractylodis Macrocephalae Rhizoma 15 g, Glycyrrhizae Radix et Rhizoma 15 g, Plantaginis Semen 12 g, Lycopi Herba 12 g, Trigonellae Semen 12 g, Cinnamomi Ramulus 10 g | Smilax glabra Roxb. [Smilacaceae]; Alisma plantago-aquatica subsp. orientale (Sam.) Sam.; Polygonatum odoratum (Mill.) Druce; Lepidii Semen (Lepidium apetalum); Atractylodes macrocephala Koidz. [Asteraceae]; Glycyrrhiza uralensis Fisch. ex DC. [Fabaceae]; Plantago asiatica L. [Plantaginaceae]; Eupatorium japonicum Thunb. [Asteraceae]; Trigonella foenum-graecum L. [Fabaceae]; Angelica sinensis (Oliv.) Diels [Apiaceae] | One dose per day, taken three times daily. The treatment observation period is 4 weeks. |
| JIN JJ 2024 | Modified Ling Gui Zhu Gan Tang | *Synopsis of Golden Chamber* |  |  |  |
| JIANG XQ 2019 | Modified Ling Gui Zhu Gan Tang | *Synopsis of Golden Chamber* | Poria 12 g, Cinnamomi Ramulus 10 g, Atractylodis Macrocephalae Rhizoma 15 g, Glycyrrhizae Radix et Rhizoma Praeparata 10 g, Salviae Miltiorrhizae Radix et Rhizoma 15 g, Pinelliae Rhizoma 10 g | Smilax glabra Roxb. [Smilacaceae]; Angelica sinensis (Oliv.) Diels [Apiaceae]; Atractylodes macrocephala Koidz. [Asteraceae]; Glycyrrhiza uralensis Fisch. ex DC. [Fabaceae]; Salvia miltiorrhiza Bunge [Lamiaceae; Salviae miltiorrhizae radix et rhizoma]; Pinellia ternata (Thunb.) Makino [Araceae] | One dose per day, taken tiwce daily (200ml per time). The treatment observation period is 1 month. |
| SU JH 2018 | Modified Ling Gui Zhu Gan Tang | *Synopsis of Golden Chamber* | Glycyrrhizae Radix et Rhizoma 10 g, Atractylodis Macrocephalae Rhizoma 10 g, Cinnamomi Ramulus 6 g, Poria 12 g | Glycyrrhiza uralensis Fisch. ex DC. [Fabaceae]; Atractylodes macrocephala Koidz. [Asteraceae]; Angelica sinensis (Oliv.) Diels [Apiaceae]; Smilax glabra Roxb. [Smilacaceae] | One dose per day, taken tiwce daily. The treatment observation period is 1 month. |
| WU XZ 2020 | Modified Ling Gui Zhu Gan Tang | *Synopsis of Golden Chamber* | Trigonellae Semen 12 g, Alismatis Rhizoma 20 g, Polygonati Odorati Rhizoma 20 g, Glycyrrhizae Radix et Rhizoma 15 g, Cinnamomi Ramulus 10 g, Plantaginis Semen 12 g, Lycopi Herba 12 g, Lepidii Semen 15 g, Atractylodis Macrocephalae Rhizoma 15 g, Poria 20 g | Trigonella foenum-graecum L. [Fabaceae]; Alisma plantago-aquatica subsp. orientale (Sam.) Sam.; Polygonatum odoratum (Mill.) Druce; Glycyrrhiza uralensis Fisch. ex DC. [Fabaceae]; Angelica sinensis (Oliv.) Diels [Apiaceae]; Plantago asiatica L. [Plantaginaceae]; Eupatorium japonicum Thunb. [Asteraceae]; Lepidii Semen (Lepidium apetalum); Atractylodes macrocephala Koidz. [Asteraceae]; Smilax glabra Roxb. [Smilacaceae] | One dose per day, taken tiwce daily. The treatment observation period is 4 weeks. |
| WU YW 2015 | Modified Ling Gui Zhu Gan Tang | *Synopsis of Golden Chamber* | Poria 12 g, Cinnamomi Ramulus 6 g, Atractylodis Macrocephalae Rhizoma (stir-fried) 10 g, Glycyrrhizae Radix et Rhizoma (honey-fried) 10 g | Smilax glabra Roxb. [Smilacaceae]; Angelica sinensis (Oliv.) Diels [Apiaceae]; Atractylodes macrocephala Koidz. [Asteraceae]; Glycyrrhiza uralensis Fisch. ex DC. [Fabaceae] | One dose per day, taken tiwce daily. The treatment observation period is 4 weeks. |
| WU ZJ 2019 | Modified Ling Gui Zhu Gan Tang | *Synopsis of Golden Chamber* | Cinnamomi Ramulus 9 g, Poria 20 g, Atractylodis Macrocephalae Rhizoma (stir-fried) 30 g, Trichosanthis Fructus 15 g, Pheretima 15 g, Salviae Miltiorrhizae Radix et Rhizoma 15 g, Glycyrrhizae Radix et Rhizoma Praeparata 10 g, Carthami Flos 12 g | Angelica sinensis (Oliv.) Diels [Apiaceae]; Smilax glabra Roxb. [Smilacaceae]; Atractylodes macrocephala Koidz. [Asteraceae]; Trichosanthes kirilowii Maxim. [Cucurbitaceae]; Pheretima; Salvia miltiorrhiza Bunge [Lamiaceae; Salviae miltiorrhizae radix et rhizoma]; Glycyrrhiza uralensis Fisch. ex DC. [Fabaceae]; Carthamus tinctorius L. [Asteraceae] | The treatment observation period is 4 weeks. |
| XIE WH 2009 | Modified Ling Gui Zhu Gan Tang | *Synopsis of Golden Chamber* | Cinnamomi Ramulus 9 g, Poria 20 g, Atractylodis Macrocephalae Rhizoma (stir-fried) 30 g, Salviae Miltiorrhizae Radix et Rhizoma 15 g, Pheretima 15 g, Carthami Flos 12 g, Trichosanthis Fructus 15 g, Glycyrrhizae Radix et Rhizoma Praeparata 10 g | Angelica sinensis (Oliv.) Diels [Apiaceae]; Smilax glabra Roxb. [Smilacaceae]; Atractylodes macrocephala Koidz. [Asteraceae]; Salvia miltiorrhiza Bunge [Lamiaceae; Salviae miltiorrhizae radix et rhizoma]; Pheretima; Carthamus tinctorius L. [Asteraceae]; Trichosanthes kirilowii Maxim. [Cucurbitaceae]; Glycyrrhiza uralensis Fisch. ex DC. [Fabaceae] | The treatment observation period is 15 days. |
| YU CL 2020 | Modified Ling Gui Zhu Gan Tang | *Synopsis of Golden Chamber* | Poria 20 g, Cinnamomi Ramulus 15 g, Atractylodis Macrocephalae Rhizoma (stir-fried) 15 g, Glycyrrhizae Radix et Rhizoma Praeparata 10 g, Salviae Miltiorrhizae Radix et Rhizoma 20 g, Acori Tatarinowii Rhizoma 15 g | Smilax glabra Roxb. [Smilacaceae]; Angelica sinensis (Oliv.) Diels [Apiaceae]; Atractylodes macrocephala Koidz. [Asteraceae]; Glycyrrhiza uralensis Fisch. ex DC. [Fabaceae]; Salvia miltiorrhiza Bunge [Lamiaceae; Salviae miltiorrhizae radix et rhizoma]; Acorus gramineus Aiton [Acoraceae] | One dose per day, taken tiwce daily. The treatment observation period is 1 month. |
| ZHOU NN 2019 | Modified Ling Gui Zhu Gan Tang | *Synopsis of Golden Chamber* | Poria 20 g, Cinnamomi Ramulus 20 g, Atractylodis Macrocephalae Rhizoma 15 g, Salviae Miltiorrhizae Radix et Rhizoma 15 g, Glycyrrhizae Radix et Rhizoma Praeparata 10 g, Acori Tatarinowii Rhizoma 10 g | Smilax glabra Roxb. [Smilacaceae]; Angelica sinensis (Oliv.) Diels [Apiaceae]; Atractylodes macrocephala Koidz. [Asteraceae]; Salvia miltiorrhiza Bunge [Lamiaceae; Salviae miltiorrhizae radix et rhizoma]; Glycyrrhiza uralensis Fisch. ex DC. [Fabaceae]; Acorus gramineus Aiton [Acoraceae] | One dose per day, taken tiwce daily. The treatment observation period is 4 weeks. |
| CHEN JL 2017 | Modified Renshen Tang | *Synopsis of Golden Chamber* | Not stated |  | Not stated |
| FEI Y 2015 | Modified Renshen Tang | *Synopsis of Golden Chamber* | Codonopsis Radix 12 g, Atractylodis Macrocephalae Rhizoma 9 g, Zingiberis Rhizoma 5 g, Glycyrrhizae Radix et Rhizoma 6 g | Codonopsis pilosula (Franch.) Nannf. [Campanulaceae]; Atractylodes macrocephala Koidz. [Asteraceae]; Zingiber officinale Roscoe [Zingiberaceae]; Glycyrrhiza uralensis Fisch. ex DC. [Fabaceae] | One dose per day, taken tiwce daily. The treatment observation period is 2 weeks. |
| WANG YW 2021 | Modified Renshen Tang | *Synopsis of Golden Chamber* | Ginseng Radix et Rhizoma 20 g, Atractylodis Macrocephalae Rhizoma 20 g, Zingiberis Rhizoma 15 g, Glycyrrhizae Radix et Rhizoma Praeparata 10 g, Allii Macrostemonis Bulbus 15 g, Trichosanthis Fructus 10 g, Salviae Miltiorrhizae Radix et Rhizoma 10 g, Corydalis Rhizoma 10 g | Panax ginseng C.A.Mey. [Araliaceae]; Atractylodes macrocephala Koidz. [Asteraceae]; Zingiber officinale Roscoe [Zingiberaceae]; Glycyrrhiza uralensis Fisch. ex DC. [Fabaceae]; Allium chinense G.Don [Amaryllidaceae]; Trichosanthes kirilowii Maxim. [Cucurbitaceae]; Salvia miltiorrhiza Bunge [Lamiaceae; Salviae miltiorrhizae radix et rhizoma]; Corydalis yanhusuo (Y.H.Chou & Chun C.Hsu) W.T.Wang ex Z.Y.Su & C.Y.Wu [Papaveraceae] | One dose per day, taken tiwce daily (150ml per time). The treatment observation period is 4 weeks. |
| WEN YY 2023 | Modified Renshen Tang | *Synopsis of Golden Chamber* | Ginseng Radix et Rhizoma 20 g, Corydalis Rhizoma 10 g, Glycyrrhizae Radix et Rhizoma Praeparata 10 g, Trichosanthis Fructus 10 g, Salviae Miltiorrhizae Radix et Rhizoma 10 g, Atractylodis Macrocephalae Rhizoma 20 g, Zingiberis Rhizoma 15 g | Panax ginseng C.A.Mey. [Araliaceae]; Corydalis yanhusuo (Y.H.Chou & Chun C.Hsu) W.T.Wang ex Z.Y.Su & C.Y.Wu [Papaveraceae]; Glycyrrhiza uralensis Fisch. ex DC. [Fabaceae]; Trichosanthes kirilowii Maxim. [Cucurbitaceae]; Salvia miltiorrhiza Bunge [Lamiaceae; Salviae miltiorrhizae radix et rhizoma]; Atractylodes macrocephala Koidz. [Asteraceae]; Zingiber officinale Roscoe [Zingiberaceae] | One dose per day, taken tiwce daily (150ml per time). The treatment observation period is 4 weeks. |
| ZHU DJ 2019 | Modified Renshen Tang | *Synopsis of Golden Chamber* | Codonopsis Radix 9 g, Zingiberis Rhizoma 9 g, Atractylodis Macrocephalae Rhizoma 9 g, Glycyrrhizae Radix et Rhizoma 9 g | Codonopsis pilosula (Franch.) Nannf. [Campanulaceae]; Zingiber officinale Roscoe [Zingiberaceae]; Atractylodes macrocephala Koidz. [Asteraceae]; Glycyrrhiza uralensis Fisch. ex DC. [Fabaceae] | One dose per day, taken tiwce daily (200ml per time). The treatment observation period is 2 weeks. |
| HU R 2004 | Modified Sini Tang | *Treatise on Typhoid Fever* | Aconiti Lateralis Radix Praeparata 10 g, Zingiberis Rhizoma 20 g, Glycyrrhizae Radix et Rhizoma Praeparata 20 g, Ginseng Radix et Rhizoma 15 g, Allii Macrostemonis Bulbus 10 g, Typhae Pollen 8 g, Notoginseng Radix et Rhizoma (powder) 1.5 g. Hospital-prepared uniform formulation, 2.8 g crude drug per mL. | Aconitum carmichaelii Debeaux (prepared); Zingiber officinale Roscoe [Zingiberaceae]; Glycyrrhiza uralensis Fisch. ex DC. [Fabaceae]; Panax ginseng C.A.Mey. [Araliaceae]; Allium chinense G.Don [Amaryllidaceae]; Typha angustifolia L. [Typhaceae]; Kalanchoe pinnata (Lam.) Pers. [Crassulaceae] | One dose per day, taken tiwce daily (100ml per time). The treatment observation period is 2 weeks. |
| JIA ZZ 2017 | Modified Sini Tang | *Treatise on Typhoid Fever* | Aconiti Lateralis Radix Praeparata 10 g (decoct first), Glycyrrhizae Radix et Rhizoma Praeparata 30 g, Zingiberis Rhizoma 20 g, Cinnamomi Ramulus 30 g, Draconis Os Crudum 30 g (decoct first), Ostreae Concha Cruda 30 g (decoct first) | Aconitum carmichaelii Debeaux (prepared); Glycyrrhiza uralensis Fisch. ex DC. [Fabaceae]; Zingiber officinale Roscoe [Zingiberaceae]; Angelica sinensis (Oliv.) Diels [Apiaceae]; Os Dracons; Ostreae Concha | One dose per day, taken tiwce daily. The treatment observation period is 3 weeks. |
| QIN J 2001 | Modified Sini Tang | *Treatise on Typhoid Fever* | Aconiti Lateralis Radix Praeparata 10 g, Zingiberis Rhizoma 6 g, Glycyrrhizae Radix et Rhizoma 4 g | Aconitum carmichaelii Debeaux (prepared); Zingiber officinale Roscoe [Zingiberaceae]; Glycyrrhiza uralensis Fisch. ex DC. [Fabaceae] | One dose per day, taken tiwce daily (100ml per time). The treatment observation period is 2 weeks. |
| SONG X 2015 | Modified Sini Tang | *Treatise on Typhoid Fever* | Zingiberis Rhizoma 15 g, Aconiti Lateralis Radix Praeparata 9 g, Glycyrrhizae Radix et Rhizoma Praeparata 10 g, Dalbergiae Odoriferae Lignum 6 g, Salviae Miltiorrhizae Radix et Rhizoma 15 g, Perillae Caulis 10 g, Amomi Fructus 10 g | Zingiber officinale Roscoe [Zingiberaceae]; Aconitum carmichaelii Debeaux (prepared); Glycyrrhiza uralensis Fisch. ex DC. [Fabaceae]; Dalbergia odorifera T.C.Chen [Fabaceae]; Salvia miltiorrhiza Bunge [Lamiaceae; Salviae miltiorrhizae radix et rhizoma]; Perilla frutescens (L.) Britton [Lamiaceae]; Wurfbainia villosa (Lour.) Škorničk. & A.D.Poulsen [Zingiberaceae] | One dose per day, taken tiwce daily. The treatment observation period is 2 weeks. |
| WANG YF 2017 | Modified Sini Tang | *Treatise on Typhoid Fever* | Glycyrrhizae Radix et Rhizoma 4 g, Zingiberis Rhizoma 6 g, Aconiti Lateralis Radix Praeparata 10 g | Glycyrrhiza uralensis Fisch. ex DC. [Fabaceae]; Zingiber officinale Roscoe [Zingiberaceae]; Aconitum carmichaelii Debeaux (prepared) | The treatment observation period is 2 weeks. |
| XV C 2021 | Modified Xiaochaihu Tang | *Treatise on Typhoid Fever* | Bupleuri Radix 20 g, Scutellariae Radix 12 g, Pinelliae Rhizoma 10 g, Ginseng Radix et Rhizoma 9 g, Glycyrrhizae Radix et Rhizoma 10 g, Zingiberis Rhizoma Recens 5 slices, Jujubae Fructus 10 pieces | Elephantopus scaber L. [Asteraceae]; Scutellaria baicalensis Georgi [Lamiaceae]; Pinellia ternata (Thunb.) Makino [Araceae]; Panax ginseng C.A.Mey. [Araliaceae]; Glycyrrhiza uralensis Fisch. ex DC. [Fabaceae]; Zingiber officinale Roscoe [Zingiberaceae]; Ziziphus jujuba Mill. [Rhamnaceae] | The treatment observation period is 4 weeks. |
| ZHANG XX 2020 | Modified Xiaochaihu Tang | *Treatise on Typhoid Fever* | Bupleuri Radix 24 g, Scutellariae Radix 9 g, Pinelliae Rhizoma 12 g, Codonopsis Radix 20 g, Glycyrrhizae Radix et Rhizoma 9 g, Zingiberis Rhizoma Recens 9 g, Cyperi Rhizoma 10 g, Aucklandiae Radix 10 g, Chuanxiong Rhizoma 20 g, Jujubae Fructus 12 pieces | Elephantopus scaber L. [Asteraceae]; Scutellaria baicalensis Georgi [Lamiaceae]; Pinellia ternata (Thunb.) Makino [Araceae]; Codonopsis pilosula (Franch.) Nannf. [Campanulaceae]; Glycyrrhiza uralensis Fisch. ex DC. [Fabaceae]; Zingiber officinale Roscoe [Zingiberaceae]; Cyperus rotundus L.; Dolomiaea costus (Falc.) Kasana & A.K.Pandey [Asteraceae]; Conioselinum anthriscoides 'Chuanxiong' [Apiaceae]; Ziziphus jujuba Mill. [Rhamnaceae] | One dose per day, taken tiwce daily. The treatment observation period is 3 weeks. |
| CAO J 2018 | Modified Xiaoxianxiong Tang | *Treatise on Typhoid Fever* | Pinelliae Rhizoma Praeparatum 10 g, Trichosanthis Fructus 10 g, Coptidis Rhizoma 10 g, Aurantii Fructus Immaturus 20 g, Acori Tatarinowii Rhizoma 10 g, Polygalae Radix 10 g, Curcumae Radix 10 g, Angelicae Sinensis Radix 10 g, Chuanxiong Rhizoma 10 g, Eupolyphaga 10 g, Hirudo 6 g, Typhae Pollen Crudum 10 g, Trogopterori Faeces 10 g, Glycyrrhizae Radix et Rhizoma Praeparata 6 g | Pinellia ternata (Thunb.) Makino [Araceae]; Trichosanthes kirilowii Maxim. [Cucurbitaceae]; Picrorhiza kurrooa Royle [Plantaginaceae]; Citrus × aurantium f. aurantium [Rutaceae]; Acorus gramineus Aiton [Acoraceae]; Polygala tenuifolia Willd.; Curcuma longa L. [Zingiberaceae]; Neolitsea cassia (L.) Kosterm. [Lauraceae]; Conioselinum anthriscoides 'Chuanxiong' [Apiaceae]; Eupolyphaga sinensis Walker; Whitmania pigra Whitman; Typha angustifolia L. [Typhaceae]; Dried feces of Trogopterus xanthipes Milne-Edwards; Glycyrrhiza uralensis Fisch. ex DC. [Fabaceae] | One dose per day, taken tiwce daily (200ml per time). The treatment observation period is 2 weeks. |
| LI AL 2019 | Modified Xiaoxianxiong Tang | *Treatise on Typhoid Fever* | Coptidis Rhizoma 6 g, Notoginseng Radix et Rhizoma 6 g, Astragali Radix 20 g, Trichosanthis Fructus 20 g, Pinelliae Rhizoma Praeparatum 12 g | Picrorhiza kurrooa Royle [Plantaginaceae]; Kalanchoe pinnata (Lam.) Pers. [Crassulaceae]; Astragalus mongholicus Bunge [Fabaceae]; Trichosanthes kirilowii Maxim. [Cucurbitaceae]; Pinellia ternata (Thunb.) Makino [Araceae] | One dose per day, taken tiwce daily (150ml per time). The treatment observation period is 4 weeks. |
| LI JJ 2010 | Modified Xiaoxianxiong Tang | *Treatise on Typhoid Fever* | Notoginseng Radix et Rhizoma (powder) 7.5 g (taken with water), Coptidis Rhizoma 5 g, Pinelliae Rhizoma 15 g, Trichosanthis Fructus 25 g, Astragali Radix 20 g | Kalanchoe pinnata (Lam.) Pers. [Crassulaceae]; Picrorhiza kurrooa Royle [Plantaginaceae]; Pinellia ternata (Thunb.) Makino [Araceae]; Trichosanthes kirilowii Maxim. [Cucurbitaceae]; Astragalus mongholicus Bunge [Fabaceae] | One dose per day, taken tiwce daily. The treatment observation period is 3 weeks. |
| LIU F 2014 | Modified Xiaoxianxiong Tang | *Treatise on Typhoid Fever* | Trichosanthis Fructus 20 g, Coptidis Rhizoma 6 g, Pinelliae Rhizoma 12 g, Salviae Miltiorrhizae Radix et Rhizoma 6 g, Carthami Flos 3 g, Dalbergiae Odoriferae Lignum 5 g | Trichosanthes kirilowii Maxim. [Cucurbitaceae]; Picrorhiza kurrooa Royle [Plantaginaceae]; Pinellia ternata (Thunb.) Makino [Araceae]; Salvia miltiorrhiza Bunge [Lamiaceae; Salviae miltiorrhizae radix et rhizoma]; Carthamus tinctorius L. [Asteraceae]; Dalbergia odorifera T.C.Chen [Fabaceae] | One dose per day, taken tiwce daily (100ml per time). The treatment observation period is 2 weeks. |
| LIU GY 2016 | Modified Xiaoxianxiong Tang | *Treatise on Typhoid Fever* | Coptidis Rhizoma 6 g, Trichosanthis Fructus 20 g, Pinelliae Rhizoma 12 g, Carthami Flos 3 g, Salviae Miltiorrhizae Radix et Rhizoma 6 g, Dalbergiae Odoriferae Lignum 5 g | Picrorhiza kurrooa Royle [Plantaginaceae]; Trichosanthes kirilowii Maxim. [Cucurbitaceae]; Pinellia ternata (Thunb.) Makino [Araceae]; Carthamus tinctorius L. [Asteraceae]; Salvia miltiorrhiza Bunge [Lamiaceae; Salviae miltiorrhizae radix et rhizoma]; Dalbergia odorifera T.C.Chen [Fabaceae] | One dose per day, taken tiwce daily (100ml per time). The treatment observation period is 2 weeks. |
| XUE RW 2018 | Modified Xiaoxianxiong Tang | *Treatise on Typhoid Fever* | Coptidis Rhizoma 6 g, Pinelliae Rhizoma Praeparatum 12 g, Trichosanthis Fructus 20 g | Picrorhiza kurrooa Royle [Plantaginaceae]; Pinellia ternata (Thunb.) Makino [Araceae]; Trichosanthes kirilowii Maxim. [Cucurbitaceae] | One dose per day, taken tiwce daily (150ml per time). The treatment observation period is 4 weeks. |
| ZHOU F 2017 | Modified Xiaoxianxiong Tang | *Treatise on Typhoid Fever* | Coptidis Rhizoma 10 g, Pinelliae Rhizoma Praeparatum 10 g, Trichosanthis Pericarpium 15 g, Trichosanthis Semen 15 g, Angelicae Sinensis Radix 10 g, Salviae Miltiorrhizae Radix et Rhizoma 15 g, Astragali Radix 30 g, Codonopsis Radix 15 g | Picrorhiza kurrooa Royle [Plantaginaceae]; Pinellia ternata (Thunb.) Makino [Araceae]; Trichosanthes kirilowii Maxim. (pericarp); Trichosanthes kirilowii Maxim. (seed); Neolitsea cassia (L.) Kosterm. [Lauraceae]; Salvia miltiorrhiza Bunge [Lamiaceae; Salviae miltiorrhizae radix et rhizoma]; Astragalus mongholicus Bunge [Fabaceae]; Codonopsis pilosula (Franch.) Nannf. [Campanulaceae] | One dose per day, taken tiwce daily (200ml per time). The treatment observation period is 30 days. |
| ZHOU WC 2021 | Modified Xiaoxianxiong Tang | *Treatise on Typhoid Fever* | Trichosanthis Fructus 20 g, Astragali Radix 20 g, Pinelliae Rhizoma Praeparatum 12 g, Notoginseng Radix et Rhizoma 6 g, Coptidis Rhizoma 6 g | Trichosanthes kirilowii Maxim. [Cucurbitaceae]; Astragalus mongholicus Bunge [Fabaceae]; Pinellia ternata (Thunb.) Makino [Araceae]; Kalanchoe pinnata (Lam.) Pers. [Crassulaceae]; Picrorhiza kurrooa Royle [Plantaginaceae] | One dose per day, taken tiwce daily (75ml per time). The treatment observation period is 4 weeks. |
| DONG M 2019 | Modified Zhigancao Tang | *Treatise on Typhoid Fever* | Glycyrrhizae Radix et Rhizoma Praeparata 30 g, Rehmanniae Radix 30 g, Ginseng Radix et Rhizoma Rubra 10 g, Jujubae Fructus 10 g, Asini Corii Colla 9 g, Ophiopogonis Radix 15 g, Cinnamomi Ramulus 10 g, Zingiberis Rhizoma Recens 6 g, Chuanxiong Rhizoma 15 g, Salviae Miltiorrhizae Radix et Rhizoma 18 g. | Glycyrrhiza uralensis Fisch. ex DC. [Fabaceae]; Rehmannia glutinosa (Gaertn.) Libosch. ex DC. [Orobanchaceae]; Panax ginseng C.A.Mey. [Araliaceae]; Ziziphus jujuba Mill. [Rhamnaceae]; Asini Corii Colla (Equus asinus); Ophiopogon japonicus (Thunb.) Ker Gawl. [Asparagaceae]; Angelica sinensis (Oliv.) Diels [Apiaceae]; Zingiber officinale Roscoe [Zingiberaceae]; Conioselinum anthriscoides 'Chuanxiong' [Apiaceae]; Salvia miltiorrhiza Bunge [Lamiaceae; Salviae miltiorrhizae radix et rhizoma] | During decoction, add 10 mL of Baijiu (42°) and soak the herbs in the liquor for 30 minutes to facilitate the dissolution of active ingredients. One dose per day, taken tiwce daily (200ml per time). The treatment observation period is 1 week. |
| GUO QB 2019 | Modified Zhigancao Tang | *Treatise on Typhoid Fever* | Not stated |  | Not stated |
| NIU LD 2020 | Modified Zhigancao Tang | *Treatise on Typhoid Fever* | Rehmanniae Radix 30 g, Glycyrrhizae Radix et Rhizoma Praeparata 30 g, Ophiopogonis Radix 15 g, Ginseng Radix et Rhizoma Rubra 10 g, Asini Corii Colla 9 g, Jujubae Fructus 10 g, Cinnamomi Ramulus 10 g, Chuanxiong Rhizoma 15 g, Zingiberis Rhizoma Recens 6 g, Salviae Miltiorrhizae Radix et Rhizoma 18 g | Rehmannia glutinosa (Gaertn.) Libosch. ex DC. [Orobanchaceae]; Glycyrrhiza uralensis Fisch. ex DC. [Fabaceae]; Ophiopogon japonicus (Thunb.) Ker Gawl. [Asparagaceae]; Panax ginseng C.A.Mey. [Araliaceae]; Asini Corii Colla (Equus asinus); Ziziphus jujuba Mill. [Rhamnaceae]; Angelica sinensis (Oliv.) Diels [Apiaceae]; Conioselinum anthriscoides 'Chuanxiong' [Apiaceae]; Zingiber officinale Roscoe [Zingiberaceae]; Salvia miltiorrhiza Bunge [Lamiaceae; Salviae miltiorrhizae radix et rhizoma] | During decoction, add 10 mL of Baijiu (42°) and soak the herbs in the liquor for 30 minutes to facilitate the dissolution of active ingredients. One dose per day, taken tiwce daily (200ml per time). The treatment observation period is 1 month. |
| WANG J 2017 | Modified Zhigancao Tang | *Treatise on Typhoid Fever* | Glycyrrhizae Radix et Rhizoma Praeparata 30 g, Ginseng Radix et Rhizoma Rubra 10 g, Cinnamomi Ramulus 10 g, Liriopes Radix 15 g, Rehmanniae Radix 30 g, Asini Corii Colla 9 g, Chuanxiong Rhizoma 15 g, Salviae Miltiorrhizae Radix et Rhizoma 18 g, Zingiberis Rhizoma Recens 6 g, Jujubae Fructus 10 pieces. | Glycyrrhiza uralensis Fisch. ex DC. [Fabaceae]; Panax ginseng C.A.Mey. [Araliaceae]; Angelica sinensis (Oliv.) Diels [Apiaceae]; Liriope sp.; Rehmannia glutinosa (Gaertn.) Libosch. ex DC. [Orobanchaceae]; Asini Corii Colla (Equus asinus); Conioselinum anthriscoides 'Chuanxiong' [Apiaceae]; Salvia miltiorrhiza Bunge [Lamiaceae; Salviae miltiorrhizae radix et rhizoma]; Zingiber officinale Roscoe [Zingiberaceae]; Ziziphus jujuba Mill. [Rhamnaceae] | During decoction, add 10 mL of Baijiu (42°) and soak the herbs in the liquor for 30 minutes to facilitate the dissolution of active ingredients. One dose per day, taken tiwce daily (200ml per time). The treatment observation period is 4 weeks. |
| ZHOU XY 2023 | Modified Zhigancao Tang | *Treatise on Typhoid Fever* | Glycyrrhizae Radix et Rhizoma Praeparata 30 g, Rehmanniae Radix 30 g, Ginseng Radix et Rhizoma Rubra 10 g, Asini Corii Colla 10 g, Jujubae Fructus 10 g, Cinnamomi Ramulus 10 g, Ophiopogonis Radix 15 g, Chuanxiong Rhizoma 15 g, Salviae Miltiorrhizae Radix et Rhizoma 15 g, Zingiberis Rhizoma Recens 5 g | Glycyrrhiza uralensis Fisch. ex DC. [Fabaceae]; Rehmannia glutinosa (Gaertn.) Libosch. ex DC. [Orobanchaceae]; Panax ginseng C.A.Mey. [Araliaceae]; Asini Corii Colla (Equus asinus); Ziziphus jujuba Mill. [Rhamnaceae]; Angelica sinensis (Oliv.) Diels [Apiaceae]; Ophiopogon japonicus (Thunb.) Ker Gawl. [Asparagaceae]; Conioselinum anthriscoides 'Chuanxiong' [Apiaceae]; Salvia miltiorrhiza Bunge [Lamiaceae; Salviae miltiorrhizae radix et rhizoma]; Zingiber officinale Roscoe [Zingiberaceae] | One dose per day, taken tiwce daily (200ml per time). The treatment observation period is 4 weeks. |
| DOU J 2023 | Modified Zhishi Xiebai Guizhi Tang | *Treatise on Typhoid Fever* | Trichosanthis Fructus 24 g, Magnoliae Officinalis Cortex 12 g, Aurantii Fructus Immaturus 12 g, Pinelliae Rhizoma 10 g, Ginseng Radix et Rhizoma 10 g, Allii Macrostemonis Bulbus 9 g, Cinnamomi Ramulus 6 g | Trichosanthes kirilowii Maxim. [Cucurbitaceae]; Magnolia officinalis Rehder & E.H.Wilson [Magnoliaceae]; Citrus × aurantium f. aurantium [Rutaceae]; Pinellia ternata (Thunb.) Makino [Araceae]; Panax ginseng C.A.Mey. [Araliaceae]; Allium chinense G.Don [Amaryllidaceae]; Angelica sinensis (Oliv.) Diels [Apiaceae] | One dose per day, taken tiwce daily (150ml per time). The treatment observation period is 3 months. |
| DOU LN 2023 | Modified Zhishi Xiebai Guizhi Tang | *Treatise on Typhoid Fever* | Aurantii Fructus Immaturus 12 g, Magnoliae Officinalis Cortex 12 g, Allii Macrostemonis Bulbus 9 g, Cinnamomi Ramulus 3 g, Trichosanthis Fructus (crushed) 12 g | Citrus × aurantium f. aurantium [Rutaceae]; Magnolia officinalis Rehder & E.H.Wilson [Magnoliaceae]; Allium chinense G.Don [Amaryllidaceae]; Angelica sinensis (Oliv.) Diels [Apiaceae]; Trichosanthes kirilowii Maxim. [Cucurbitaceae] | One dose per day, taken tiwce daily (150ml per time). The treatment observation period is 2 weeks. |
| DU MW 2022 | Modified Zhishi Xiebai Guizhi Tang | *Treatise on Typhoid Fever* | Morindae Officinalis Radix 12 g, Aurantii Fructus Immaturus 12 g, Magnoliae Officinalis Cortex 12 g, Epimedii Folium 15 g, Cinnamomi Ramulus 6 g, Allii Macrostemonis Bulbus 9 g, Trichosanthis Fructus 24 g | Gynochthodes officinalis (F.C.How) Razafim. & B.Bremer [Rubiaceae]; Citrus × aurantium f. aurantium [Rutaceae]; Magnolia officinalis Rehder & E.H.Wilson [Magnoliaceae]; Epimedium brevicornu Maxim.; Angelica sinensis (Oliv.) Diels [Apiaceae]; Allium chinense G.Don [Amaryllidaceae]; Trichosanthes kirilowii Maxim. [Cucurbitaceae] | One dose per day, taken tiwce daily (100ml per time). The treatment observation period is 2 months. |
| JIA F 2021 | Modified Zhishi Xiebai Guizhi Tang | *Treatise on Typhoid Fever* | Trichosanthis Fructus 24 g, Allii Macrostemonis Bulbus 9 g, Aurantii Fructus Immaturus 12 g, Magnoliae Officinalis Cortex 12 g, Cinnamomi Ramulus 6 g | Trichosanthes kirilowii Maxim. [Cucurbitaceae]; Allium chinense G.Don [Amaryllidaceae]; Citrus × aurantium f. aurantium [Rutaceae]; Magnolia officinalis Rehder & E.H.Wilson [Magnoliaceae]; Angelica sinensis (Oliv.) Diels [Apiaceae] | One dose per day, taken tiwce daily (100ml per time). The treatment observation period is 2 months. |
| LI FT 2021 | Modified Zhishi Xiebai Guizhi Tang | *Treatise on Typhoid Fever* | Trichosanthis Fructus 24 g, Allii Macrostemonis Bulbus 9 g, Aurantii Fructus Immaturus 12 g, Magnoliae Officinalis Cortex 12 g, Cinnamomi Ramulus 6 g | Trichosanthes kirilowii Maxim. [Cucurbitaceae]; Allium chinense G.Don [Amaryllidaceae]; Citrus × aurantium f. aurantium [Rutaceae]; Magnolia officinalis Rehder & E.H.Wilson [Magnoliaceae]; Angelica sinensis (Oliv.) Diels [Apiaceae] | One dose per day, taken tiwce daily (100ml per time). The treatment observation period is 2 months. |
| LI YC 2021 | Modified Zhishi Xiebai Guizhi Tang | *Treatise on Typhoid Fever* | Trichosanthis Fructus 30 g, Aurantii Fructus Immaturus 15 g, Allii Macrostemonis Bulbus 15 g, Magnoliae Officinalis Cortex 15 g, Cinnamomi Ramulus 10 g, Ginseng Radix et Rhizoma Rubra (slice) 10 g, Atractylodis Macrocephalae Rhizoma (stir-fried with bran) 10 g, Glycyrrhizae Radix et Rhizoma Praeparata 10 g | Trichosanthes kirilowii Maxim. [Cucurbitaceae]; Citrus × aurantium f. aurantium [Rutaceae]; Allium chinense G.Don [Amaryllidaceae]; Magnolia officinalis Rehder & E.H.Wilson [Magnoliaceae]; Angelica sinensis (Oliv.) Diels [Apiaceae]; Panax ginseng C.A.Mey. [Araliaceae]; Atractylodes macrocephala Koidz. [Asteraceae]; Glycyrrhiza uralensis Fisch. ex DC. [Fabaceae] | One dose per day, taken tiwce daily (200ml per time). The treatment observation period is 3 months. |
| LIU JN 2023 | Modified Zhishi Xiebai Guizhi Tang | *Treatise on Typhoid Fever* | Trichosanthis Pericarpium 12 g, Trichosanthis Semen 12 g, Aurantii Fructus Immaturus 12 g, Magnoliae Officinalis Cortex 12 g, Chuanxiong Rhizoma 9 g, Cyperi Rhizoma 9 g, Acori Tatarinowii Rhizoma 9 g, Allii Macrostemonis Bulbus 9 g, Carthami Flos 6 g, Cinnamomi Ramulus 6 g | Trichosanthes kirilowii Maxim. (pericarp); Trichosanthes kirilowii Maxim. (seed); Citrus × aurantium f. aurantium [Rutaceae]; Magnolia officinalis Rehder & E.H.Wilson [Magnoliaceae]; Conioselinum anthriscoides 'Chuanxiong' [Apiaceae]; Cyperus rotundus L.; Acorus gramineus Aiton [Acoraceae]; Allium chinense G.Don [Amaryllidaceae]; Carthamus tinctorius L. [Asteraceae]; Angelica sinensis (Oliv.) Diels [Apiaceae] | One dose per day, taken tiwce daily (125ml per time). The treatment observation period is 2 weeks. |
| LU S 2011 | Modified Zhishi Xiebai Guizhi Tang | *Treatise on Typhoid Fever* | Trichosanthis Fructus 24 g, Allii Macrostemonis Bulbus 9 g, Aurantii Fructus Immaturus 12 g, Magnoliae Officinalis Cortex 12 g, Cinnamomi Ramulus 6 g | Trichosanthes kirilowii Maxim. [Cucurbitaceae]; Allium chinense G.Don [Amaryllidaceae]; Citrus × aurantium f. aurantium [Rutaceae]; Magnolia officinalis Rehder & E.H.Wilson [Magnoliaceae]; Angelica sinensis (Oliv.) Diels [Apiaceae] | One dose per day, taken tiwce daily (150ml per time). The treatment observation period is 2 weeks. |
| MA XX 2018 | Modified Zhishi Xiebai Guizhi Tang | *Treatise on Typhoid Fever* | Trichosanthis Fructus 23 g, Allii Macrostemonis Bulbus 9 g, Aurantii Fructus Immaturus 12 g, Magnoliae Officinalis Cortex 12 g, Cinnamomi Ramulus 3 g, Coptidis Rhizoma 6 g | Trichosanthes kirilowii Maxim. [Cucurbitaceae]; Allium chinense G.Don [Amaryllidaceae]; Citrus × aurantium f. aurantium [Rutaceae]; Magnolia officinalis Rehder & E.H.Wilson [Magnoliaceae]; Angelica sinensis (Oliv.) Diels [Apiaceae]; Picrorhiza kurrooa Royle [Plantaginaceae] | One dose per day, taken tiwce daily (200 ml per time). The treatment observation period is 2 weeks. |
| SHI YP 2009 | Modified Zhishi Xiebai Guizhi Tang | *Treatise on Typhoid Fever* | Aurantii Fructus Immaturus, Magnoliae Officinalis Cortex, Allii Macrostemonis Bulbus, Cinnamomi Ramulus, Trichosanthis Fructus, Pinelliae Rhizoma Praeparatum, Notoginseng Radix et Rhizoma, Ginseng Radix et Rhizoma | Citrus × aurantium f. aurantium [Rutaceae]; Magnolia officinalis Rehder & E.H.Wilson [Magnoliaceae]; Allium chinense G.Don [Amaryllidaceae]; Angelica sinensis (Oliv.) Diels [Apiaceae]; Trichosanthes kirilowii Maxim. [Cucurbitaceae]; Pinellia ternata (Thunb.) Makino [Araceae]; Kalanchoe pinnata (Lam.) Pers. [Crassulaceae]; Panax ginseng C.A.Mey. [Araliaceae] | One dose per day, taken tiwce daily (150 ml per time). The treatment observation period is 4 weeks. |
| TAN XD 2019 | Modified Zhishi Xiebai Guizhi Tang | *Treatise on Typhoid Fever* | Trichosanthis Fructus 24 g, Allii Macrostemonis Bulbus 9 g, Aurantii Fructus Immaturus 12 g, Magnoliae Officinalis Cortex 12 g, Cinnamomi Ramulus 6 g | Trichosanthes kirilowii Maxim. [Cucurbitaceae]; Allium chinense G.Don [Amaryllidaceae]; Citrus × aurantium f. aurantium [Rutaceae]; Magnolia officinalis Rehder & E.H.Wilson [Magnoliaceae]; Angelica sinensis (Oliv.) Diels [Apiaceae] | One dose per day, taken tiwce daily (200 ml per time). The treatment observation period is 2 months. |
| WANG XH 2009 | Modified Zhishi Xiebai Guizhi Tang | *Treatise on Typhoid Fever* | Trichosanthis Fructus 30 g, Allii Macrostemonis Bulbus 15 g, Cinnamomi Ramulus 12 g, Aurantii Fructus Immaturus 12 g, Salviae Miltiorrhizae Radix et Rhizoma 30 g, Dalbergiae Odoriferae Lignum 15 g, Persicae Semen 10 g, Carthami Flos 12 g, Vinum (Huangjiu) appropriate amount | Trichosanthes kirilowii Maxim. [Cucurbitaceae]; Allium chinense G.Don [Amaryllidaceae]; Angelica sinensis (Oliv.) Diels [Apiaceae]; Citrus × aurantium f. aurantium [Rutaceae]; Salvia miltiorrhiza Bunge [Lamiaceae; Salviae miltiorrhizae radix et rhizoma]; Dalbergia odorifera T.C.Chen [Fabaceae]; Juglans regia L. [Juglandaceae]; Carthamus tinctorius L. [Asteraceae]; Vinum | The treatment observation period is 4 weeks. |
| WANG YG 2013 | Modified Zhishi Xiebai Guizhi Tang | *Treatise on Typhoid Fever* | Aconiti Lateralis Radix Praeparata 9 g, Cinnamomi Ramulus 6 g, Aurantii Fructus Immaturus 9 g, Magnoliae Officinalis Cortex 9 g, Trichosanthis Fructus 15 g, Allii Macrostemonis Bulbus 9 g, Poria 9 g, Atractylodis Macrocephalae Rhizoma 6 g, Salviae Miltiorrhizae Radix et Rhizoma 30 g, Mori Ramulus 30 g, Glycyrrhizae Radix et Rhizoma 6 g | Aconitum carmichaelii Debeaux (prepared); Angelica sinensis (Oliv.) Diels [Apiaceae]; Citrus × aurantium f. aurantium [Rutaceae]; Magnolia officinalis Rehder & E.H.Wilson [Magnoliaceae]; Trichosanthes kirilowii Maxim. [Cucurbitaceae]; Allium chinense G.Don [Amaryllidaceae]; Smilax glabra Roxb. [Smilacaceae]; Atractylodes macrocephala Koidz. [Asteraceae]; Salvia miltiorrhiza Bunge [Lamiaceae; Salviae miltiorrhizae radix et rhizoma]; Morus alba L. [Moraceae]; Glycyrrhiza uralensis Fisch. ex DC. [Fabaceae] | One dose per day, taken tiwce daily. The treatment observation period is 15 days. |
| WU SK 2015 | Modified Zhishi Xiebai Guizhi Tang | *Treatise on Typhoid Fever* | Trichosanthis Fructus 24 g, Aurantii Fructus Immaturus 12 g, Magnoliae Officinalis Cortex 12 g, Pinelliae Rhizoma 10 g, Ginseng Radix et Rhizoma 10 g, Allii Macrostemonis Bulbus 9 g, Cinnamomi Ramulus 6 g | Trichosanthes kirilowii Maxim. [Cucurbitaceae]; Citrus × aurantium f. aurantium [Rutaceae]; Magnolia officinalis Rehder & E.H.Wilson [Magnoliaceae]; Pinellia ternata (Thunb.) Makino [Araceae]; Panax ginseng C.A.Mey. [Araliaceae]; Allium chinense G.Don [Amaryllidaceae]; Angelica sinensis (Oliv.) Diels [Apiaceae] | One dose per day, taken tiwce daily (150 ml per time). The treatment observation period is 4 weeks. |
| WU YJ 2020 | Modified Zhishi Xiebai Guizhi Tang | *Treatise on Typhoid Fever* | Not stated |  | One dose per day, taken tiwce daily (150 ml per time). The treatment observation period is 2 weeks. |
| YU YS 2022 | Modified Zhishi Xiebai Guizhi Tang | *Treatise on Typhoid Fever* | Aurantii Fructus Immaturus 12 g, Magnoliae Officinalis Cortex 12 g, Trichosanthis Fructus 12 g, Allii Macrostemonis Bulbus 9 g, Cinnamomi Ramulus 6 g | Citrus × aurantium f. aurantium [Rutaceae]; Magnolia officinalis Rehder & E.H.Wilson [Magnoliaceae]; Trichosanthes kirilowii Maxim. [Cucurbitaceae]; Allium chinense G.Don [Amaryllidaceae]; Angelica sinensis (Oliv.) Diels [Apiaceae] | One dose per day, taken tiwce daily (200 ml per time). The treatment observation period is 4 weeks. |
| YUAN QY 2021 | Modified Zhishi Xiebai Guizhi Tang | *Treatise on Typhoid Fever* | Trichosanthis Fructus 10 g, Cinnamomi Ramulus 10 g, Aurantii Fructus Immaturus 15 g, Allii Macrostemonis Bulbus 15 g, Salviae Miltiorrhizae Radix et Rhizoma 15 g, Puerariae Lobatae Radix 15 g, Pinelliae Rhizoma Praeparatum 15 g, Glycyrrhizae Radix et Rhizoma Praeparata 3 g | Trichosanthes kirilowii Maxim. [Cucurbitaceae]; Angelica sinensis (Oliv.) Diels [Apiaceae]; Citrus × aurantium f. aurantium [Rutaceae]; Allium chinense G.Don [Amaryllidaceae]; Salvia miltiorrhiza Bunge [Lamiaceae; Salviae miltiorrhizae radix et rhizoma]; Pueraria montana var. lobata (Willd.) Maesen & S.M.Almeida ex Sanjappa & Predeep [Fabaceae]; Pinellia ternata (Thunb.) Makino [Araceae]; Glycyrrhiza uralensis Fisch. ex DC. [Fabaceae] | One dose per day, taken tiwce daily (150 ml per time). The treatment observation period is 2 weeks. |
| ZHANG LH 2024 | Modified Zhishi Xiebai Guizhi Tang | *Treatise on Typhoid Fever* | Trichosanthis Fructus 20 g, Allii Macrostemonis Bulbus 20 g, Aurantii Fructus Immaturus 15 g, Magnoliae Officinalis Cortex 10 g, Salviae Miltiorrhizae Radix et Rhizoma 20 g, Carthami Flos 15 g, Cinnamomi Ramulus 10 g, Pinelliae Rhizoma 10 g | Trichosanthes kirilowii Maxim. [Cucurbitaceae]; Allium chinense G.Don [Amaryllidaceae]; Citrus × aurantium f. aurantium [Rutaceae]; Magnolia officinalis Rehder & E.H.Wilson [Magnoliaceae]; Salvia miltiorrhiza Bunge [Lamiaceae; Salviae miltiorrhizae radix et rhizoma]; Carthamus tinctorius L. [Asteraceae]; Angelica sinensis (Oliv.) Diels [Apiaceae]; Pinellia ternata (Thunb.) Makino [Araceae] | One dose per day, taken tiwce daily (150 ml per time). The treatment observation period is 4 weeks. |
| ZHANG TW 2019 | Modified Zhishi Xiebai Guizhi Tang | *Treatise on Typhoid Fever* | Aurantii Fructus Immaturus 15 g, Magnoliae Officinalis Cortex 15 g, Allii Macrostemonis Bulbus 12 g, Trichosanthis Fructus 30 g, Pinelliae Rhizoma 15 g, Cinnamomi Ramulus 20 g, Aconiti Lateralis Radix Praeparata 9 g | Citrus × aurantium f. aurantium [Rutaceae]; Magnolia officinalis Rehder & E.H.Wilson [Magnoliaceae]; Allium chinense G.Don [Amaryllidaceae]; Trichosanthes kirilowii Maxim. [Cucurbitaceae]; Pinellia ternata (Thunb.) Makino [Araceae]; Angelica sinensis (Oliv.) Diels [Apiaceae]; Aconitum carmichaelii Debeaux (prepared) | The treatment observation period is 4 weeks. |
| ZHOU HY 2015 | Modified Zhishi Xiebai Guizhi Tang | *Treatise on Typhoid Fever* | Trichosanthis Fructus 12 g, Allii Macrostemonis Bulbus 9 g, Cinnamomi Ramulus 12 g, Aurantii Fructus Immaturus 12 g, Magnoliae Officinalis Cortex 12 g, Aconiti Lateralis Radix Praeparata 9 g, Angelicae Sinensis Radix 12 g, Chuanxiong Rhizoma 10 g, Codonopsis Radix 12 g, Atractylodis Macrocephalae Rhizoma 12 g | Trichosanthes kirilowii Maxim. [Cucurbitaceae]; Allium chinense G.Don [Amaryllidaceae]; Angelica sinensis (Oliv.) Diels [Apiaceae]; Citrus × aurantium f. aurantium [Rutaceae]; Magnolia officinalis Rehder & E.H.Wilson [Magnoliaceae]; Aconitum carmichaelii Debeaux (prepared); Neolitsea cassia (L.) Kosterm. [Lauraceae]; Conioselinum anthriscoides 'Chuanxiong' [Apiaceae]; Codonopsis pilosula (Franch.) Nannf. [Campanulaceae]; Atractylodes macrocephala Koidz. [Asteraceae] | One dose per day, taken tiwce daily (150 ml per time). The treatment observation period is 4 weeks. |
| ZHOU LH 2023 | Modified Zhishi Xiebai Guizhi Tang | *Treatise on Typhoid Fever* | Aurantii Fructus Immaturus 12 g, Magnoliae Officinalis Cortex 12 g, Allii Macrostemonis Bulbus 9 g, Cinnamomi Ramulus 3 g, Trichosanthis Fructus 12 g | Citrus × aurantium f. aurantium [Rutaceae]; Magnolia officinalis Rehder & E.H.Wilson [Magnoliaceae]; Allium chinense G.Don [Amaryllidaceae]; Angelica sinensis (Oliv.) Diels [Apiaceae]; Trichosanthes kirilowii Maxim. [Cucurbitaceae] | One dose per day, taken tiwce daily (150 ml per time). The treatment observation period is 4 weeks. |
| ZHU DJ 2018-1 | Modified Zhishi Xiebai Guizhi Tang | *Treatise on Typhoid Fever* | Trichosanthis Pericarpium 12 g, Trichosanthis Semen 12 g, Allii Macrostemonis Bulbus 9 g, Aurantii Fructus Immaturus 12 g, Magnoliae Officinalis Cortex 12 g, Cinnamomi Ramulus 6 g | Trichosanthes kirilowii Maxim. (pericarp); Trichosanthes kirilowii Maxim. (seed); Allium chinense G.Don [Amaryllidaceae]; Citrus × aurantium f. aurantium [Rutaceae]; Magnolia officinalis Rehder & E.H.Wilson [Magnoliaceae]; Angelica sinensis (Oliv.) Diels [Apiaceae] | One dose per day, taken tiwce daily (90 ml per time). The treatment observation period is 2 weeks. |
| ZHU DJ 2018-2 | Modified Zhishi Xiebai Guizhi Tang | *Treatise on Typhoid Fever* | Trichosanthis Pericarpium 12 g, Trichosanthis Semen 12 g, Allii Macrostemonis Bulbus 9 g, Aurantii Fructus Immaturus 12 g, Magnoliae Officinalis Cortex 12 g, Cinnamomi Ramulus 6 g | Trichosanthes kirilowii Maxim. (pericarp); Trichosanthes kirilowii Maxim. (seed); Allium chinense G.Don [Amaryllidaceae]; Citrus × aurantium f. aurantium [Rutaceae]; Magnolia officinalis Rehder & E.H.Wilson [Magnoliaceae]; Angelica sinensis (Oliv.) Diels [Apiaceae] | One dose per day, taken tiwce daily (45 ml per time). The treatment observation period is 2 weeks. |
