# Supplementary material for: Comparative efficacy of Traditional Chinese Medicine classical prescriptions for angina pectoris: a network meta-analysis
Source: Front Pharmacol. 2026 Sep 28;17:1794371. doi: 10.3389/fphar.2026.1794371 (PMC13654956; doi:10.3389/fphar.2026.1794371)
Supplement: Supplementary file 1 [file DataSheet1.ZIP › Supplementary material/data2_Table S3_included research.docx]

**Table S3** Characteristics of included studies.

| Study ID | Age (year) | | random method | Sample size | | Intervention | | period (day) | Outcomes | Sub-group |
| --- | --- | --- | --- | --- | --- | --- | --- | --- | --- | --- |
|  | T | C |  | T | C | T | C |  |  |  |
| CHEN XB  2020(1) | 57.4±4.6 | 56.2±3.6 | not mentioned | 30 | 30 | Biomedicine+DGSN | Biomedicine | 28 | ④⑤⑥⑦ | SAP |
| CUI JK  2020(2) | 55.83±3.17 | 53.83±4.29 | not mentioned | 34 | 34 | Biomedicine+DGSN | Biomedicine | 21 | ③ | SAP |
| DU H  2022(3) | 65.81±6.24 | 64.87±6.51 | Random number table | 39 | 39 | Biomedicine+DGSN | Biomedicine | 14 | ②③ | UA |
| JIA XY  2020(4) | 58.17±6.58 | 61.07±6.40 | doll's random table of clinical cases | 30 | 30 | Biomedicine+DGSN | Biomedicine | 28 | ①②③④⑥⑦⑨ | SAP |
| SUN XH  2022(5) | 67.56±9.37 | 68.19±9.56 | Randomized admission sequence grouping | 25 | 25 | Biomedicine+DGSN | Biomedicine | 90 | ③④⑤⑥⑦⑨ | SAP |
| YUAN QM  2020(6) | 56.41±8.87 | 60.39±8.04 | Random number table | 34 | 33 | Biomedicine+DGSN | Biomedicine | 28 | ①②③⑨ | UA |
| CHEN LP  2022(7) | 54.84±3.96 | 54.92±3.84 | Random number table | 50 | 50 | Biomedicine+GLXBBJ | Biomedicine | 20 | ④⑤⑥⑦ | SAP |
| HE MX  2017(8) | 56.8±7.2 | 57.3±7.8 | not mentioned | 37 | 37 | Biomedicine+GLXBBJ | Biomedicine | 75 | ①④⑤⑥⑦ | SAP |
| HU J  2018(9) | 57.0±11.1 | 56.3±10.7 | Computer table | 45 | 45 | Biomedicine+GLXBBJ | Biomedicine | 14 | ④⑤⑥⑦⑨ | UA |
| LI SB  2015(10) | 68.7±13.2 | 67.9±12.8 | not mentioned | 83 | 83 | Biomedicine+GLXBBJ | Biomedicine | 56 | ④⑤⑥⑦ | SAP |
| YANG WQ  2021(11) | 68.32±2.98 | 67.35±3.26 | not mentioned | 40 | 40 | Biomedicine+GLXBBJ | Biomedicine | 60 | ① | SAP |
| YUE SM  2021(12) | 70.26±3.25 | 70.32±3.10 | not mentioned | 40 | 38 | Biomedicine+GLXBBJ | Biomedicine | 14 | ④⑤⑥⑦ | SAP |
| CAO PZ  2021(13) | 58.5±10.8 | 57,6±10.2 | not mentioned | 30 | 30 | Biomedicine+GLXBBX | Biomedicine | 28 | ④⑥⑦ | SAP |
| QIU WF  2021(14) | 65.74±6.35 | 66.77±6.74 | Random number table | 42 | 42 | Biomedicine+GLXBBX | Biomedicine | 30 | ⑧ | SAP |
| DU FC  2022(15) | 60.25±6.84 | 60.36±6.75 | Random number table | 68 | 68 | Biomedicine+GLXBBX | Biomedicine | 56 | ③ | SAP |
| DU Y  2022(16) | 60.51±7.02 | 60.33±6.98 | not mentioned | 38 | 38 | Biomedicine+GLXBBX | Biomedicine | 28 | ④⑤⑥⑦ | SAP |
| GAN TM  2015(17) | 52.5±8.9 | 51.2±8.4 | not mentioned | 30 | 30 | Biomedicine+GLXBBX | Biomedicine | 30 | ④⑤⑥⑦ | SAP |
| GU Y  2022(18) | 63.25±5.17 | 63.17±5.19 | Random number table | 30 | 30 | Biomedicine+GLXBBX | Biomedicine | 28 | ⑧ | SAP |
| GUO CJ  2024(19) | 62.35±3.16 | 62.24±3.08 | Random number table | 63 | 63 | Biomedicine+GLXBBX | Biomedicine | 28 | ④⑥⑦⑨ | SAP |
| HE J  2017(20) | 61.62±5.48 | 60.48±5.23 | not mentioned | 48 | 41 | Biomedicine+GLXBBX | Biomedicine | 14 | ④⑤⑥⑦ | SAP |
| HUANG PP  2020(21) | 65.31±2.24 | 63.31±2.32 | not mentioned | 40 | 40 | Biomedicine+GLXBBX | Biomedicine | 28 | ②⑨ | SAP |
| JIANG A  2013(22) | 60.35±12.71 | 59.46±12.37 | not mentioned | 50 | 50 | Biomedicine+GLXBBX | Biomedicine | 28 | ①② | SAP |
| LI JL  2009(23) | 57.8 | 56.9 | not mentioned | 60 | 60 | Biomedicine+GLXBBX | Biomedicine | 28 | ①② | UA |
| LI RM  2020(24) | 55.3±6.11 | 56.4±7.28 | Random number table | 49 | 49 | Biomedicine+GLXBBX | Biomedicine | 28 | ⑤⑥⑦ | SAP |
| LI SQ  2021(25) | 55.84±10.18 | 57.06±11.11 | doll's random table of clinical cases | 32 | 33 | Biomedicine+GLXBBX | Biomedicine | 28 | ①②③ | SAP |
| LI W  2018(26) | 60.65±6.86 | 57.50±7.64 | Random number table | 37 | 38 | Biomedicine+GLXBBX | Biomedicine | 28 | ①②③④⑤⑥⑦⑧⑨ | SAP |
| LIU LH  2023(27) | 56.42±4.39 | 56.39±4.43 | not mentioned | 41 | 40 | Biomedicine+GLXBBX | Biomedicine | 28 | ②③ | SAP |
| LIU YL  2020(28) | 58.71±4.18 | 60.29±4.52 | Random number table | 120 | 120 | Biomedicine+GLXBBX | Biomedicine | 90 | ③ | UA |
| LONG C  2019(29) | 57.37±5.71 | 58.47±7.49 | Random number table | 30 | 30 | Biomedicine+GLXBBX | Biomedicine | 28 | ②③④⑥⑦⑨ | SAP |
| LU TX  2017(30) | 61.00±4.85 | 63.80±5.04 | Random number table | 30 | 30 | Biomedicine+GLXBBX | Biomedicine | 14 | ①②③⑨ | SAP |
| MENG M  2012(31) | 54.13±4.55 | 54.30±4.70 | not mentioned | 30 | 30 | Biomedicine+GLXBBX | Biomedicine | 28 | ①②⑨ | SAP |
| MI XS  2011(32) | 无 | 无 | doll's random table of clinical cases | 30 | 30 | Biomedicine+GLXBBX | Biomedicine | 28 | ①②③⑨ | SAP |
| NIU YW  2022(33) | 62.51±12.25 | 61.63±13.47 | not mentioned | 63 | 63 | Biomedicine+GLXBBX | Biomedicine | 28 | ②⑨ | SAP |
| SHU JB  2024(34) | 47.77±6.35 | 47.65±6.21 | Random number table | 44 | 44 | Biomedicine+GLXBBX | Biomedicine | 28 | ④⑥⑦ | SAP |
| SU W  2016(35) | 66.48±8.73 | 64.87±7.24 | Random number table | 33 | 31 | Biomedicine+GLXBBX | Biomedicine | 14 | ③ | UA |
| TIAN DF  2016(36) | 46-74 | 44-76 | Random number table | 43 | 43 | Biomedicine+GLXBBX | Biomedicine | 28 | ②③⑨ | SAP |
| WANG AM  2024(37) | 62.64±10.56 | 62.32±10.24 | Computer table | 20 | 20 | Biomedicine+GLXBBX | Biomedicine | 28 | ④⑤⑥⑦ | SAP |
| WANG CG  2020(38) | 61.29±9.28 | | Coin-tossing method | 53 | 53 | Biomedicine+GLXBBX | Biomedicine | 30 | ③ | SAP |
| WANG EG  2017(39) | 62.6±3.5 | | Random number table | 50 | 50 | Biomedicine+GLXBBX | Biomedicine | 90 | ③ | SAP |
| WANG P  2021(40) | 64.15±7.23 | 63.74±7.78 | Random number table | 33 | 31 | Biomedicine+GLXBBX | Biomedicine | 30 | ①③④⑥⑦⑨ | UA |
| WANG SM  2021(41) | NA | NA | not mentioned | 27 | 37 | Biomedicine+GLXBBX | Biomedicine | 28 | ① | SAP |
| WANG X  2019(42) | 61.69±6.34 | 61.75±5.42 | Random number table | 33 | 33 | Biomedicine+GLXBBX | Biomedicine | 30 | ④⑤⑥⑦ | SAP |
| WEI ZH  2024(43) | 63.48±4.31 | 63.52±4.38 | Lottery method | 37 | 37 | Biomedicine+GLXBBX | Biomedicine | 90 | ⑤⑥⑦ | SAP |
| WEI M  2009(44) | 42-70 | 45-72 | not mentioned | 65 | 54 | Biomedicine+GLXBBX | Biomedicine | 14 | ①②⑨ | UA |
| WU T  2023(45) | 56.82±10.9 | 59.6±10.3 | Random number table | 30 | 30 | Biomedicine+GLXBBX | Biomedicine | 28 | ④⑤⑥⑦ | SAP |
| XV J  2016(46) | 63.23±4.94 | 64.34±4.23 | not mentioned | 40 | 40 | Biomedicine+GLXBBX | Biomedicine | 90 | ⑤⑥⑦⑧ | SAP |
| YAN XS  2018(47) | 63.7±6.4 | | Randomized admission sequence grouping | 54 | 54 | Biomedicine+GLXBBX | Biomedicine | 28 | ①④⑤⑥⑦ | SAP |
| YAN JG  2023(48) | 58.61±4.89 | 58.43±4.56 | not mentioned | 50 | 50 | Biomedicine+GLXBBX | Biomedicine | 60 | ⑧ | UA |
| YANG FQ  2015(49) | 58.3±5.7 | 56.8±6.2 | Random number table | 40 | 40 | Biomedicine+GLXBBX | Biomedicine | 60 | ① | SAP |
| YANG JK  2015(50) | 60.43±9.64 | 61.09±8.94 | The dice method | 35 | 35 | Biomedicine+GLXBBX | Biomedicine | 28 | ①②⑨ | SAP |
| YANG QF  2015(51) | 55.9±5.7 | 56.7±5.8 | not mentioned | 42 | 42 | Biomedicine+GLXBBX | Biomedicine | 14 | ①② | SAP |
| YANG XY  2007(52) | 60.54±2.82 | 61.37±2.63 | Randomized admission sequence grouping | 48 | 42 | Biomedicine+GLXBBX | Biomedicine | 56 | ④⑤⑥⑦⑨ | SAP |
| YANG X  2023(53) | 63.02±8.57 | 63.34±7.45 | Random number table | 37 | 38 | Biomedicine+GLXBBX | Biomedicine | 28 | ①②③④⑤⑥⑦⑨ | UA |
| ZHANG XF  2024(54) | 50.06±5.38 | 49.84±5.54 | Random number table | 50 | 50 | Biomedicine+GLXBBX | Biomedicine | 20 | ③ | SAP |
| ZHANG ZF  2016(55) | 58.2±7.7 | 57.3±8.2 | Random number table | 31 | 31 | Biomedicine+GLXBBX | Biomedicine | 30 | ①②③⑨ | SAP |
| ZHAO HY  2024(56) | 62.78±5.88 | 63.27±5.16 | Touch ball method | 43 | 43 | Biomedicine+GLXBBX | Biomedicine | 60 | ④⑤⑥⑦ | SAP |
| ZHAO YL  2013(57) | 59.47±8.41 | 60.97±6.551 | not mentioned | 30 | 30 | Biomedicine+GLXBBX | Biomedicine | 28 | ②③⑧⑨ | SAP |
| ZHAO YG  2024(58) | 56.24±9.33 | 56.47±9.12 | Random number table | 40 | 40 | Biomedicine+GLXBBX | Biomedicine | 30 | ④⑥⑦⑨ | SAP |
| ZHENG XB  2020(59) | 61.45 | 62.95 | Random number table | 40 | 40 | Biomedicine+GLXBBX | Biomedicine | 56 | ④⑤⑥⑦⑨ | SAP |
| ZHOU HT  2006(60) | 55.3 | 54.9 | not mentioned | 40 | 40 | Biomedicine+GLXBBX | Biomedicine | 30 | ①②③ | UA |
| ZHU DJ  2021(61) | 62.88±7.97 | 63.41±8.00 | Random number table | 32 | 32 | Biomedicine+GLXBBX | Biomedicine | 14 | ③ | UA |
| ZHU ZY  2019(62) | 60.9 | 62.4 | not mentioned | 30 | 30 | Biomedicine+GLXBBX | Biomedicine | 28 | ①③ | SAP |
| ZHUANG HB  2020(63) | 62.59±4.15 | 62.24±4.68 | not mentioned | 46 | 47 | Biomedicine+GLXBBX | Biomedicine | 30 | ④⑤⑥⑦ | SAP |
| HUANG PY  2008(64) | 56.57±7.33 | 53.86±7.91 | not mentioned | 60 | 60 | Biomedicine+GZFL | Biomedicine | 28 | ④⑤⑥⑦⑨ | SAP |
| LU XY  2012(65) | 55.8±10.10 | 55.82±10.13 | Random number table | 78 | 78 | Biomedicine+GZFL | Biomedicine | 28 | ①⑨ | SAP |
| PEI Q  2013(66) | 62.72±8.48 | 60.58±7.03 | Random number table | 36 | 36 | Biomedicine+GZFL | Biomedicine | 28 | ④⑤⑥⑦⑨ | UA |
| XV GY  2017(67) | 52.6±6.9 | | not mentioned | 35 | 35 | Biomedicine+GZFL | Biomedicine | 30 | ①②③ | UA |
| XV Y  2023(68) | 40-73 | 43-75 | not mentioned | 36 | 38 | Biomedicine+GZFL | Biomedicine | 28 | ①②③④⑤⑥⑦⑨ | SAP |
| YU P  2014(69) | 无 | 无 | not mentioned | 33 | 33 | Biomedicine+GZFL | Biomedicine | 28 | ①②③ | SAP |
| ZHENG WD  2015(70) | 56.57±7.33 | 53.86±7.91 | not mentioned | 120 | 120 | Biomedicine+GZFL | Biomedicine | 28 | ④⑤⑥⑦⑨ | SAP |
| ZHONG F  2022(71) | 67.62±4.10 | 67.15±5.23 | not mentioned | 75 | 75 | Biomedicine+GZFL | Biomedicine | 90 | ③④⑤⑥⑦⑨ | SAP |
| ZHOU WR  2017(72) | 53±6.13 | 51.97±5.8 | Random number table | 30 | 30 | Biomedicine+GZFL | Biomedicine | 28 | ③⑨ | SAP |
| AN LJ  (73)2017 | 57.8 | 58.6 | Random number table | 34 | 34 | Biomedicine+LGZG | Biomedicine | 28 | ①②⑨ | SAP |
| DU LY  2019(74) | 59.18±4.62 | 58.27±4.31 | Random number table | 62 | 63 | Biomedicine+LGZG | Biomedicine | 30 | ⑤⑥⑦ | SAP |
| HUA MZ  2023(75) | 61.10±1.33 | 60.57±1.23 | Random number table | 48 | 48 | Biomedicine+LGZG | Biomedicine | 28 | ⑨ | SAP |
| JIANG XQ  2019(76) | 64.08±5.91 | 63.25±5.43 | not mentioned | 46 | 46 | Biomedicine+LGZG | Biomedicine | 28 | ①②⑨ | SAP |
| SU JH  2018(77) | 50.4±7.2 | 50.3±7.1 | Random number table | 41 | 41 | Biomedicine+LGZG | Biomedicine | 28 | ①④⑤⑥⑦ | SAP |
| WU XZ  2020(78) | 54.67±6.03 | 53.69±5.84 | Random number table | 64 | 64 | Biomedicine+LGZG | Biomedicine | 28 | ④⑤⑥⑦ | SAP |
| WU YW  2015(79) | 50.1±7.2 | | not mentioned | 58 | 58 | Biomedicine+LGZG | Biomedicine | 28 | ①②④⑤⑥⑦ | SAP |
| WU ZJ  2019(80) | 63.3±5.57 | 63.21±6.51 | not mentioned | 30 | 30 | Biomedicine+LGZG | Biomedicine | 28 | ④⑤⑥⑦⑧ | SAP |
| XIE WH  2009(81) | 52.32±5.68 | 52.32±5.68 | Random number table | 60 | 60 | Biomedicine+LGZG | Biomedicine | 15 | ④⑥⑦ | UA |
| YU CL  2020(82) | 64.15±12.03 | 64.22±12.10 | Random number table | 45 | 45 | Biomedicine+LGZG | Biomedicine | 28 | ①②⑨ | SAP |
| ZHOU NN  2019(83) | 58.6±3.0 | 57.9±2.8 | not mentioned | 52 | 51 | Biomedicine+LGZG | Biomedicine | 28 | ④⑤⑥⑦ | SAP |
| CHEN JL  2017(84) | 62.68±7.56 | 65.22±6.94 | Random number table | 34 | 32 | Biomedicine+RS | Biomedicine | 14 | ④⑤⑥⑦ | UA |
| FEI Y  2015(85) | 64.17±12.19 | 62.50±12.15 | Random number table | 30 | 30 | Biomedicine+RS | Biomedicine | 14 | ③④⑤⑥⑦⑨ | UA |
| WANG YW  2021(86) | 56.89±5.26 | 54.39±5.71 | Random number table | 32 | 32 | Biomedicine+RS | Biomedicine | 28 | ④⑤⑥⑦⑨ | SAP |
| WEN YY  2023(87) | 65.6±1.7 | 65.5±1.8 | Random number table | 38 | 38 | Biomedicine+RS | Biomedicine | 28 | ③④⑤⑥⑦⑨ | SAP |
| ZHU DJ  2019(88) | 65.67 | 63.49 | Random number table | 30 | 30 | Biomedicine+RS | Biomedicine | 14 | ④⑤⑥⑦ | UA |
| HU R  2004(89) | 59.38±10.66 | 60.22±10.81 | Even-odd number random grouping | 51 | 51 | Biomedicine+SN | Biomedicine | 14 | ①② | SAP |
| JIA ZZ  2017(90) | 52.4±7.96 | 53.9±8.47 | not mentioned | 43 | 43 | Biomedicine+SN | Biomedicine | 21 | ② | SAP |
| QIN J  2001(91) | 57.2±7.2 | 59.6±5.8 | not mentioned | 23 | 23 | Biomedicine+SN | Biomedicine | 14 | ①② | SAP |
| SONG X  2015(92) | 58.4±5.4 | 58.2±5.2 | Random number table | 60 | 60 | Biomedicine+SN | Biomedicine | 14 | ①②⑨ | SAP |
| WANG YF  2017(93) | 56 | 55 | not mentioned | 20 | 20 | Biomedicine+SN | Biomedicine | 14 | ①② | SAP |
| XV C  2021(94) | 50.12±2.57 | 49.78±2.41 | Random number table | 50 | 50 | Biomedicine+XCH | Biomedicine | 56 | ④⑥⑦⑨ | SAP |
| ZHANG XX  2020(95) | 61.7±8.3 | 63.6±9.1 | Random number table | 61 | 61 | Biomedicine+XCH | Biomedicine | 21 | ④⑥⑦ | SAP |
| CAO J  2018(96) | 59.5±3.8 | 61.5±2.0 | Random number table | 35 | 35 | Biomedicine+XXX | Biomedicine | 14 | ②③ | SAP |
| LI AL  2019(97) | 56.3±9.2 | 56.3±9.2 | not mentioned | 49 | 49 | Biomedicine+XXX | Biomedicine | 28 | ① | SAP |
| LI JJ  2010(98) | 57±7.6 | 56±8.8 | not mentioned | 30 | 30 | Biomedicine+XXX | Biomedicine | 21 | ③⑨ | UA |
| LIU F  2014(99) | 55.7±3.55 | 55.6±3.7 | not mentioned | 35 | 35 | Biomedicine+XXX | Biomedicine | 14 | ①③⑨ | UA |
| LIU GY  2016(100) | 56.25±4.62 | 56.35±4.64 | Random number table | 30 | 30 | Biomedicine+XXX | Biomedicine | 28 | ①③ | UA |
| XUE RW  2018(101) | 56.16±7.19 | 55.94±8.45 | Random number table | 43 | 40 | Biomedicine+XXX | Biomedicine | 28 | ①② | SAP |
| ZHOU F  2017(102) | 63.1±3.2 | 62.8±2.7 | not mentioned | 28 | 28 | Biomedicine+XXX | Biomedicine | 30 | ①②③⑨ | UA |
| ZHOU WC  2021(103) | 56.35±7.12 | 56.04±7.36 | not mentioned | 48 | 48 | Biomedicine+XXX | Biomedicine | 28 | ① | UA |
| DONG M  2019(104) | 67.3±3.5 | 67.5±3.3 | not mentioned | 40 | 40 | Biomedicine+ZGC | Biomedicine | 7 | ③ | UA |
| GUO QB  2019(105) | 67.3±4.7 | 66.3±4.5 | Lottery method | 60 | 60 | Biomedicine+ZGC | Biomedicine | 7 | ③ | UA |
| NIU LD  2020(106) | 58.64±3.57 | 58.50±5.8 | Random number table | 25 | 25 | Biomedicine+ZGC | Biomedicine | 30 | ④⑥⑦ | UA |
| WANG J  2017(107) | 64.6±11.1 | 65.4±10.8 | Random number table | 32 | 32 | Biomedicine+ZGC | Biomedicine | 28 | ④⑥⑦ | UA |
| ZHOU XY  2023(108) | 63.57±6.01 | 62.14±6.22 | Random number table | 38 | 38 | Biomedicine+ZGC | Biomedicine | 28 | ④⑥⑦ | UA |
| DOU J  2023(109) | 63.24±2.24 | 63.19±2.31 | not mentioned | 18 | 18 | Biomedicine+ZSXBGZ | Biomedicine | 114 | ④⑥⑦ | SAP |
| DOU LN  2023(110) | 62.8±4.85 | 62.63±4.47 | not mentioned | 43 | 43 | Biomedicine+ZSXBGZ | Biomedicine | 14 | ⑧⑨ | SAP |
| DU MW  2022(111) | 57.68±6.13 | 57.34±6.04 | not mentioned | 40 | 40 | Biomedicine+ZSXBGZ | Biomedicine | 56 | ⑧ | SAP |
| JIA F  2021(112) | 50.150±14.624 | 50.400±15.380 | Random number table | 22 | 21 | Biomedicine+ZSXBGZ | Biomedicine | 60 | ④⑤⑥⑦⑨ | SAP |
| LI FT  2021(113) | 58.14±6.33 | 58.22±6.47 | Random number table | 34 | 34 | Biomedicine+ZSXBGZ | Biomedicine | 60 | ⑧ | SAP |
| LI YC  2021(114) | 64.19±6.25 | 63.25±6.33 | Random number table | 60 | 60 | Biomedicine+ZSXBGZ | Biomedicine | 90 | ②⑧⑨ | SAP |
| LIU JN  2023(115) | 54.58±15.77 | 55.02±15.49 | not mentioned | 106 | 112 | Biomedicine+ZSXBGZ | Biomedicine | 14 | ③⑨ | UA |
| LU S  2011(116) | 无 | 无 | not mentioned | 33 | 31 | Biomedicine+ZSXBGZ | Biomedicine | 14 | ①③⑧ | UA |
| MA XX  2018(117) | 67.13±6.74 | 65.10±6.90 | Random number table | 33 | 31 | Biomedicine+ZSXBGZ | Biomedicine | 14 | ②③④⑤⑥⑦⑧⑨ | SAP |
| SHI YP  2009(118) | 55.09±10.33 | 54.66±10.27 | not mentioned | 30 | 26 | Biomedicine+ZSXBGZ | Biomedicine | 28 | ②③④⑥⑦ | SAP |
| TAN XD  2019(119) | 58.53±8.56 | 60.83±8.63 | Random number table | 81 | 81 | Biomedicine+ZSXBGZ | Biomedicine | 60 | ③④⑤⑥⑦⑨ | SAP |
| WANG XH  2009(120) | 60±11 | 59±9 | not mentioned | 50 | 50 | Biomedicine+ZSXBGZ | Biomedicine | 28 | ①② | SAP |
| WANG YG  2013(121) | 30-79 | 29-81 | not mentioned | 45 | 45 | Biomedicine+ZSXBGZ | Biomedicine | 15 | ①②⑨ | UA |
| WU SK  2015(122) | 62.84±6.76 | 63.91±5.63 | not mentioned | 57 | 57 | Biomedicine+ZSXBGZ | Biomedicine | 28 | ④⑤⑥⑦ | SAP |
| WU YJ  2020(123) | 60.94±3.34 | 61.84±3.05 | not mentioned | 31 | 32 | Biomedicine+ZSXBGZ | Biomedicine | 14 | ①②③⑧⑨ | SAP |
| YU YS  2022(124) | 63.38±5.32 | 63.25±5.21 | Random number table | 50 | 50 | Biomedicine+ZSXBGZ | Biomedicine | 28 | ④⑥⑦⑧ | UA |
| YUAN QY  2021(125) | 49.63±5.41 | 51.09±5.81 | Random number table | 81 | 80 | Biomedicine+ZSXBGZ | Biomedicine | 14 | ②③⑨ | UA |
| ZHANG LH  2024(126) | 65.32±6.98 | 62.91±6.85 | not mentioned | 34 | 35 | Biomedicine+ZSXBGZ | Biomedicine | 28 | ②③⑨ | SAP |
| ZHANG TW  2019(127) | 45.28±4.1 | 49.27±3.6 | not mentioned | 30 | 30 | Biomedicine+ZSXBGZ | Biomedicine | 28 | ①⑤⑥⑦ | SAP |
| ZHOU HY  2015(128) | 64.53±5.37 | 65.33±6.74 | not mentioned | 30 | 30 | Biomedicine+ZSXBGZ | Biomedicine | 28 | ①②③④⑤⑥⑦⑨ | UA |
| ZHOU LH  2023(129) | 29.02±3.15 | 28.18±2.39 | not mentioned | 38 | 35 | Biomedicine+ZSXBGZ | Biomedicine | 28 | ⑧⑨ | UA |
| ZHU DJ  2018-1(130) | 62.88±7.97 | 62.90±7.94 | Random number table | 32 | 30 | Biomedicine+ZSXBGZ | Biomedicine | 14 | ⑧⑨ | UA |
| ZHU DJ  2018-2(131) | 66.62±10.36 | 67.89±9.96 | Random number table | 30 | 30 | Biomedicine+ZSXBGZ | Biomedicine | 14 | 1. ③⑧ | UA |

* T. Trial group；C. Control group；SAP. Stable angina; UA. Unstable angina; ①Angina pectoris efficiency rate；②ECG efficiency rate；③TCM syndrome efficiency rate；④LDL-C；⑤HDL-C；⑥TG；⑦TC；⑧Hs-CRP；⑨Safety.

1. Xuebin C, Lianshe L, Mingjun Z, Ruiwen X. Study On The Effect Of Danggui Sini Decoction On Coronary Heart Disease And Its Mechanism. Modern Journal Of Integrated Traditional Chinese And Western Medicine. 2020;29(7):698-701.
2. Jiankun C, Chunying W, Yaru L. Clinical Observation Of Modified DangguiSini Decoction In TheTreatmentof Cold Coagulated Heart Vessel Type Of Microvascular Angina Pector. Journal Of Emergency In Traditional Chinese. 2021;30(11):1987-90.
3. Hong D, Rui L, Hongyan Z, Hongyuan M, Yuanyuan Y. Clinical Observation Of Modified Danggui Sini Decoction In The Treatment Of Unstable Angina Pectoris Of Coronary Heart Disease (Yin Cold Stagnation). Journal Of Emergency In Traditional Chinese. 2022;31(5):875-7.
4. Xiaoya J. Clinical Observation On The Treatment Of Stable Angina Pectoris With Yinhan Congzhi Syndrome By Modified Danggui Sini Decoction [Master's Degree]2020.
5. Xiuhua S, Hao Z, Yuan M, Linyan Q, Lihong W, Min F. Observation On The Therapeutic Effect Of Danggui Sini Decoction Combined With Western Medicine In The Treatment Of Stable Angina Pectoris Of Coronary Heart Disease. Zhejiang Clinical Medical Journal. 2022;24(7):1035-7.
6. Qimin Y. Clinical Study Of Angelica Sini Decoction In The Treatment Of Unstable Angina Pectoris Of Yin -Cold -Stagnation Coronary Heart Disease [Master's Degree]2020.
7. Liping C, Xiaole Z, Le H, Li Z. Clinical Value Analysis Of Gualou Xiebai Baijiu Decoction In Treatment Of Angina Pectoris Of Coronary Heart Disease. Practical Journal Of Internal Medicine In Traditional Chinese Medicine. 2022;36(9):132-5.
8. Mingxin H. The Therapeutic Effect Of Gualou Xiebai Baijiu Decoction On Angina Pectoris Of Coronary Heart Disease. Heilongjiang Medicine And Pharmacy. 2017;40(5):95-6.
9. Jun H. Clinical Observation On 45 Cases Of Angina Pectoris Treated By Integrative Chinese And Western Medicine. Chinese Journal Of Ethnomedicine And Ethnopharmacy. 2018;27(20):85-6.
10. Shubin L. To Observe The Curative Effect Of Gualou Xiebai Baijiu Decoction In Treating Angina Pectoris Of Coronary Heart Disease. Cardiovascular Disease Electronic Journal Of Integrated Traditional Chinese And Western Medicine. 2015;3(24):134-5.
11. Wanquan Y, Danke Y. Clinical Observation On The Treatment Of Coronary Heart Disease Angina Pectoris With Integrated Traditional Chinese And Western Medicine. Journal Of Practical Traditional Chinese Medicine. 2017;33(6):707-8.
12. Shumei Y. Observation On The Effect Of Gualou Xiebai Baijiu Decoction In The Treatment Of Elderly Patients With Angina Pectoris Of Coronary Heart Disease. Clinical Medical Engineering. 2021;28(2):205-6.
13. Peizhen C. Clinical Observation Of Gualou Xiebai Banxia Decoction Combined With Metoprolol In The Treatment Of Coronary Heart Disease Angina Pectoris With Phlegm Turbidity Obstruction. Yunnan Journal Of Traditional Chinese Medicine And Materia Medica. 2021;42(9).
14. Weifeng Q, Junqiang L. Clinical Effect Of Gualou Xiebai Banxia Decoction Combined With Atorvastatin In The Treatment Of Angina Pectoris Of Coronary Heart Disease And Its Influence On Micro-Inflammatory Reaction Of Patients. Hainan Medical Journal. 2021;32(17):2197-200.
15. Fengchuan D, Peipei S, Jingle Z. To Study The Control Effect Of Modified Gualou Xiebai Banxia Decoction Combined With Western Medicine On Stable Angina Pectoris With Phlegm And Blood Stasis. Chinese Journal Of Public Health Engineering. 2022;21(3):505-7.
16. Yuan D. Analysis Of The Therapeutic Effect Of Modified Gualou Xiebai Banxia Decoction On Angina Pectoris Of Coronary Heart Disease. Heilongjiang Medicine And Pharmacy. 2022;45(3):59-60.
17. Taomei G. The Influence Of Modified Guazhuo Xiebai Banxia Decoction On Myocardial Enzyme Levels And Blood Lipids In Patients With Coronary Heart Disease And Angina Pectoris Due To Internal Obstruction Of Phlegm-Turbidity. Tibetan Medicine. 2015(3):82-4.
18. Yan G, Dianhua L, Xiaoling S, Hong W, Jiansong G. Effect Of Gualou Xiebai Banxia Decoction Combined With Conventional Western Medicine On Stable Angina Pectoris (Phlegm Blocking Heart Vessel). Basic Traditional Chinese Medicine. 2022;1(8):29-33.
19. Changjiang G, Junfeng Z, Yuan F. Clinical Observation On The Modified Gualou Xiebai Banxia Decoction In The Treatment Of Coronary Heart Disease With Angina Pectoris Due To Phlegm Stasis Obstruction. Journal Of Practical Traditional Chinese Medicine. 2024;40(3):485-7.
20. Jun H. Observation On The Therapeutic Effect Of Integrative Traditional Chinese And Western Medicine In Treating Angina Pectoris Of Coronary Heart Disease. Medical Journal Of Chinese People's Health. 2017;29(13):60-2.
21. Peipei H, Yanfeng Z, Yuqin L. Modified Gualou Xiebai Banxia Decoction For The Treatment Of Angina Pectoris In Patients With Coronary Artery Atherosclerotic Heart Disease: 40 Cases. Hunan Journal Of Traditional Chinese Medicine. 2020;36(3):41-3.
22. Ao J. Clinical Observation On 50 Cases Of Stable Angina Pectoris Of Coronary Heart Disease Treated By Integrated Traditional Chinese And Western Medicine. Health Horizon. 2013;21(3):184-5.
23. Junlan L. Clinical Observation On The Treatment Of Unstable Angina Pectoris With Modified Gualou Xiebai Banxia Decoction. Heilongjiang Journal Of Traditional Chinese. 2009(5):10-1.
24. Rongmin L, Jiazhen L. Observation On The Therapeutic Effect Of Modified Gualou Xiebai Banxia Decoction In Treating Angina Pectoris Of Coronary Heart Disease (Phlegm Stasis Obstruction Syndrome). Inner Mongolia Journal Of Traditional Chinese. 2020;39(9):24-5.
25. Shiqi L. Evaluation Of The Therapeutic Effect Of Jiajian Huazhi Xiebai Banxia Decoction In The Treatment Of Unstable Angina Pectoris With The Syndrome Of Mutual Entanglement Of Phlegm And Blood [Master's Degree]2021.
26. Wei L. Research Of Flavored GualouXiebaiBanxia Decoction In The Treatment Of Stable Angina Pectoris And The Influence Of Hs-CRP [Master's Degree]2018.
27. Lihong L, Yan C. Effect Of Gualou Xiebai Banxia Decoction On Stable Angina Pectoris After Percutaneous Coronary Intervention. Journal Of Practical Traditional Chinese Internal Medicine. 2023;37(5):111-3.
28. Yunlong L. The Application Effect Of Guāliú Xièyī Shānào Decoction In The Treatment Of Unstable Angina Pectoris Caused By Phlegm Turbidity Obstruction. Henan Medical Research. 2020;29(24):4553-5.
29. Chen L. Clinical Observation On The Supplementation Of Trichosanthes Fruit Decoction For Treating Training Qi Deficiency And Phlegm-Stasis Type Thoracic Obstruction (Stable Angina Pectoris) [Master's Degree]2019.
30. Tongxing L. The Clinical Study On Treating Stabl Angina Pectoris Of Coronary Heart Disease With Added Gualou Xiebai Banxia Decoction [Master's Degree]2017.
31. Meng M. The Clinical Study Of The Effect Of Jia Wei Gualou Xiebai Banxia Tang On Diastolic Function Of Left Ventricle In Stable Angina Pectoris Patients [Master's Degree]2012.
32. Xueshan M, Zhihua G. Thirty Cases Of Stable Angina Pectoris In Coronary Heart Disease Treated With The Method Of Resolving Phlegm And Removing Blood Stasis. Chinese Medicine Modern Distance Education Of China. 2011;9(7):22-3.
33. Yangwei N. Observation On The Therapeutic Effect Of Modified Gualou Xiebai Banxia Decoction In The Treatment Of Coronary Heart Disease With Angina Pectoris Of Phlegm Turbidity Obstruction Type. Journal Of Practical Traditional Chinese Medicine. 2022;38(4):599-601.
34. Junbo S. Clinical Study On The Treatment Of Recurrent Angina Pectoris In Patients With Coronary Heart Disease After PCI Surgery With Modified Gualou Xiebai Banxia Decoction. Modern Diagnosis And Treatment. 2024;35(11):1608-10.
35. Wei S, Feng G, Chungang Z, Hongjun Z. The Influence Of Guanhuo Xiebai Banxia Decoction On The Plasma Levels Of MMP-9 And TIMP-1 In Patients With Unstable Angina Pectoris Of Phlegm Turbidity Obstruction Type. Journal Of Li-Shizhen Traditional Chinese Medicine. 2016;27(4):890-2.
36. Danfeng T, Guomin S, Min P. Clinical Observation Of Modified Gualou-Xiebai-Banxia Decoction For Refractory Angina With Phlegm-Blood Stasis Type In Coronary Heart Disease Patients After Percutaneous Coronary Intervention. Hebei Journal Of Traditional Chinese Medicine. 2016;38(9):1361-4.
37. Aimei W. A Study On The Therapeutic Effect Of Modified Gualou Xiebai Banxia Decoction In Treating Angina Pectoris Of Coronary Heart Disease With Phlegm Obstruction Syndrome. Special Health. 2024(2):95-6.
38. Chunguang W, Qiusheng Z, Ying L. Observation On The Therapeutic Effect Of Modified Gualou Xiebai Banxia Decoction On Patients With Chest Pain Of Heart Yang Deficiency Type. Special Health. 2020(18):87-8.
39. Enguang W, Sijing Y, Haian D. The Clinical Efficacy Of Modified Gualou Xiebai Banxia Decoction In The Treatment Of Angina Pectoris Of Coronary Heart Disease. Inner Mongolia Journal Of Traditional Chinese. 2017;36(10):32.
40. Ping W. Clinical Study Of Modified Gualou Xiebai Banxia Decoction In The Treatment Of Coronary Heart Disease Patients With Angina Pectoris [Master's Degree]2021.
41. Sumei W. Clinical Study On The Symptom Improvement Of Patients With Chest Pain Due To Phlegm-Turbidity Blockage Type Angina Pectoris Treated With Gualou Xiebai Banxia Decoction. New Mom And New Born. 2021(5):128.
42. Xin W, Yuhang W, Chengwen C. Effect Of Modified Gualou Xiebai Banxia Decoction On Blood Lipid Level And Hemorheology In Patients With Coronary Heart Disease And Angina Pectoris. Heilongjiang Medical Journal. 2019;43(10):1219-20,22.
43. Zhaohong W. The Clinical Effect Of Modified Gualou Xiebai Banxia Decoction In The Treatment Of Angina Pectoris Due To Coronary Heart Disease. Inner Mongolia Journal Of Traditional Chinese Medicine. 2024;43(4):8-9.
44. Ming W, Ping Y, Juxin W, Jinrong Y. Modified Gualou Xiebai Banxia Decoction For The Treatment Of Unstable Angina Pectoris. Chinese Journal Of Integrative Medicine On Cardio-Cerebrovascular Disease. 2009;7(10):1218-9.
45. Tian W, Xiaoping P. Effects Of Gualou Xiebai Banxia Decoction On Stable Angina Pectoris. Chinese Journal Of Medicinal Guide. 2023;25(4):420-4.
46. Jing X. Clinical Observation On The Treatment Of Coronary Heart Disease Angina Pectoris With Modified Gualou Xiebai Banxia Decoction. Asia-Pacific Traditional Medicine. 2016;12(21):130-1.
47. Xiaoshe Y. Effect Of Gualou Xiebai Banxia Decoction On MMP-9 And TIMP-1 In Patients With Phlegm Congestion Type Chest Stuffiness. Clinical Research And Practice. 2018;3(19):130-1.
48. Jiaohuan Y. Clinical Efficacy Analysis Of Modified Guanlu Xiebai Banxia Decoction In The Treatment Of Unstable Angina Pectoris. Contemporary Medicine Forum. 2023;21(15):3-6.
49. Fuqiang Y. The Xinjia Gualou Xiebai Banxia Decoction Was Used As The Main Treatment For 80 Cases Of Coronary Heart Disease With Angina Pectoris. China Medical Engineering. 2015;23(8):125,7.
50. Jiankun Y. Gualouxiebai Banxia Soup Combined With Western Treatment Angina Pectoris Randomized Controlled Study. Journal Of Practical Traditional Chinese Internal Medicine. 2015(12):107-9.
51. Qiongfen Y, Defei B, Hongbo W, Shiliang X. Clinical Efficacy Observation Of Gualou Xiebai Banxia Decoction Combined With Nitroglycerin In The Treatment Of 42 Cases Of Coronary Heart Disease Angina Pectoris. Guide Of China Medicine. 2015;13(1):228.
52. Xiaoyan Y. Clinical Study On The Modified Formula Of Gu Wei Xie Bai Ban Xia Decoction In Treating Stable Angina Pectoris Of Coronary Heart Disease Caused By Phlegm Obstruction [Master's Degree]2007.
53. Xiao Y. Clinical Study On The Treatment Of Unstable Angina Based On "State Target Theory" [Master's Degree]2023.
54. Xingfei Z. The Application Of Gualou Xiebai Banxia Decoction In The Clinical Treatment Of Angina Pectoris In Coronary Heart Disease. The World Of Healthy Living. 2024(16):89-90.
55. Zhifang Z, Jianhua Z, Hua X, Longjiang Q, Xiaohong B, Yonghong Z. Gualou-Xiebai-Banxia Decoction Combined With Conventional Therapy For The Patients With Coronary Heart Disease Angina Pectoris (Stagnancy Of Qi And Blood Stasis) And Heart Failure. International Journal Of Traditional Chinese Medicine. 2016;38(12):1076-9.
56. Heyun Z. Effect Of Western Medicine Combined With Added Gualou Xyebai Banxia Decoction On Patients With Angina Pectoris Syndrome Of Phlegm-Stasis Interjunction After Coronary Heart Disease PCI Diet Health. 2024(11):93-6.
57. Yanlin Z. The Influence Of Flavored Snakegourd Fruit Longstamen Onion Bulb Pinellia Decoction On Inflammatory Factors In Patients With Coronary Heart Disease SAP [Master's Degree]2013.
58. Yonggang Z, Yong C, Yalu L. The Effect Of Modified Gualou Xiebai Banxia Decoction On Stable Angina Pectoris And Its Influence On Cardiac Function Of Patients. Doctor. 2024;9(17):87-90.
59. Xiaobo Z. Clinical Observation On Gualou Xiebai Banxia Decoction Combined With Western Medicine In The Treatment Of Stable Angina Pectoris Of Turbid Phlegm Obstruction Syndrome. Chinese Medicine Modern Distance Education Of China. 2020;18(24):134-7.
60. Hongtu Z. Summary Of 40 Cases Treated With Integrated Traditional Chinese And Western Medicine For Spontaneous Angina Pectoris. Hunan Journal Of Traditional Chinese Medicine. 2006;22(4):14-5.
61. De-jian Z, Qiang W, Shi-rong M, Di Q, Qin-yue C, Yao-yao H, Et Al. The Influence Of Trichosanthes And Chinese Chive And Pinellia Decoction On MMP-9 And TIMP-1 Of Unstable Angina. Henan Traditional Chinese Medicine. 2021;41(3):357-60.
62. Zhiyao Z, Ning L, Kexin L. Clinical Observation Of Angina Pectoris Of Coronary Heart Disease Of Phlegm And Blood Stasis Syndrome Treated With Modified Gualou Xiebai Banxia Tang. World Journal Of Integrated Traditional And Western Medicine. 2019;14(8):1131-3.
63. Hongbiao Z. Observation On The Effect Of Guanhuo Xiebai Banxia Decoction In Treating Angina Pectoris Of Coronary Heart Disease. Practical Clinical Journal Of Integrated Traditional Chinese And Western Medicine. 2020;20(14):13-4.
64. Peiyi H. The Treatment Of 60 Cases Of Stable Exertional Angina Pectoris With Modified Guizhi Fuzhen Decoction. Chinese Journal Of Integrative Medicine On Cardio-Cerebrovascular Disease. 2008;6(10):1207-8.
65. Xiangying L. Clinical Study On The Treatment Of Coronary Heart Disease Angina Pectoris With Modified Guizhi Fuling Decoction. Chinese Journal Of Clinical Research. 2012;25(7):709-10.
66. Qiang P, Wenfeng S, Xide Z. Effects Of Guizhi Fuling Decoction On YKL-40 And Hs-CRP Of Patients With Non-ST Segment Elevation Acute Coronary Syndrome. Chinese Journal Of Integrated Traditional And Western Medicine. 2013;33(2):186-90.
67. Guangyu X, Yutong M. Clinical Efficacy Study Of Guizhi Fuling Decoction In Patients With Unstable Angina Pectoris Syndrome Characterized By Blood Stasis And Heat Congestion. Contemporary Medicine. 2017;23(18):119-21.
68. Ya X. Clinical Study On Xiaoxianxiong Decoction For Treatment Of Refractory Angina Pectoris With Qi Stagnation And Blood Stasis Syndrome [Master's Degree]2023.
69. Peng Y, Haixiao L, Xiaohu C. The Clinical Efficacy Of Guizhi Fuling Decoction In Treating The Syndrome Of Unstable Angina Pectoris With Mutual Obstruction Of Stasis And Heat, And Its Impact On Quality Of Life. Chinese Journal Of Integrative Medicine On Cardio-Cerebrovascular Disease. 2014;12(8):929-31.
70. Wenda Z, Yucheng Z, Kaian K. Observation On The Therapeutic Effect Of Wenchong Huaxue Method In Treating 240 Cases Of Stable Ischemic Angina Pectoris. Guide Of China Medicine. 2015;13(12):191-2.
71. Feng Z. Clinical Study Of Guizhi Fuling Capsule Combined With Trimetazidine In The Treatment Of Angina Pectoris Of Coronary Heart Disease With Phlegm Stasis Obstruction. Journal Of Rare And Uncommon Diseases. 2022;29(4):24-6.
72. Wenrui Z. Clinical Study Of Guizhi Fuling Pill In The Treatment Of Blood Stasis Obstructing Type Chest Stuffy Pain. Shanghai Medical & Pharmaceutical Journal. 2017;38(20):17-9.
73. Lijuan A, Juan L. Clinical Observation Of Flavored Linggui Zhugan Decoction In Treatment Of Coronary Heart Disease. Journal Of Liaoning University Of TCM. 2017;19(03):182-4.
74. Laiyi D, Jie H, Guiqing W, Baojun S. The Effect Of Modified Linggui Zhugan Decoction On Blood Lipid And Hemorheology In Patients With Angina Pectoris Of Coronary Heart Disease. Modern Diagnosis And Treatment. 2019;30(24):4282-3.
75. Mingzhang H. The Value Of Modified Linggui Zhu Gan Decoction In The Treatment Of Patients With Angina Pectoris. Contemporary Medicine. 2023;29(18):45-7.
76. Xiuqin J, Shuqin C. Clinical Research On The Modified Linggui Zhugan Decoction In The Treatment Of Angina Pectoris In Coronary Heart Disease. Prevention And Treatment Of Cardiovascular Disease. 2019;9(30):31-3.
77. Jvhong S. Observation On The Clinical Efficacy Of Modified Linggui Shugan Decoction Combined With Western Medicine In The Treatment Of Angina Pectoris Of Coronary Heart Disease. Chinese Journal Of Clinical Rational Drug Use. 2018;11(25).
78. Xiaozhu W. Clinical Efficacy Analysis Of Modified Linggui Zhuanshan Decoction In The Treatment Of Angina Pectoris Of Coronary Heart Disease. Prevention And Treatment Of Cardiovascular Disease. 2020;10(27):5-6.
79. Yiwen W, Tao L. Observation Of Clinical Effect Of Lingguizhugan Decoction Combined With Western Medicine In The Treatment Of Coronary Heart Disease Stenocardia. Chinese Journal Of Modern Drug Application. 2015;9(11):11-3.
80. Zhijing W. Observation On The Therapeutic Effect Of Combining Chinese And Western Medicines In The Treatment Of Unstable Angina Pectoris. Journal Of Practical Traditional Chinese Medicine. 2019;35(08):996-7.
81. Weihong X. Observation On The Therapeutic Effect Of Modified Linggui Shugan Decoction In Treating 60 Cases Of Unstable Angina Pectoris. Journal Of Emergency In Traditional Chinese Medicine. 2009;18(07):1047+9.
82. Changli Y, Jianjun L. Observation On The Clinical Efficacy Of Modified Linggui Zhugan Decoction Combined With Trimetazidine Hydrochloride Tablets In The Treatment Of Angina Pectoris Of Phlegm Obstructing Heart Meridian Type. Journal Of Sichuan Of Traditional Chinese Medicine. 2020;38(02):80-2.
83. Nanan Z, Jing S. Effect Analysis Of Linggui Zhugan Decoction Modified On Angina Pectoris And Coronary Heart Disease. Heilongjiang Science. 2019;10(16):52-3.
84. Jialin C. Effect Of Ginseng Decoction On Atherogenic Index Of Plasma In Patients With Unstable Angina Pectoris Of Xu Han Type [Master's Degree]2017.
85. Yuan F. Clinical Efficacy Observation Of Ginseng Decoction In The Treatment Of Patients With Unstable Angina Pectoris (With Deficiency-Cold Syndrome). Journal Of Emergency In Traditional Chinese. 2015(4):739-40,41.
86. Yingwei W, Zemin Z, Bo W, Yunhui L, Lufei L. Clinical Observation On The Treatment Of Stable Angina Pectoris Of Coronary Heart Disease With Modified Ginseng Decoction. Information On Traditional Chinese Medicine. 2021;38(2):58-61.
87. Yanyun W. Effect Analysis Of Modified Ginseng Decoction Combined With Western Medicine In Treating Stable Angina Pectoris Of Coronary Heart Disease. Chinese Community Doctors. 2023;39(9):70-2,6.
88. Dejian Z, Wei C, Qi W, SX, Hao C. Clinical Study Of Renshen Decoction In The Treatment Of Unstable Angina Pectoris Of Deficiency Of Heart-Yang And Kidney-Yang. Information On Traditional Chinese Medicine. 2019;36(4):70-3.
89. Rui H. Clinical Observation On The Influence Of Sini Decoction On Cardiac Contractility In Coronary Heart Disease Patients With Angina Pectoris. Tianjin Journal Of Traditional Chinese Medicine. 2004;21(1):28-30.
90. Zhuangzhuang J, Zhigang W. Observation On The Therapeutic Effect Of 43 Cases Of Coronary Heart Disease With Angina Pectoris Of Heart-Kidney Yang Deficiency Treated By Warming Kidney Method. Hunan Journal Of Traditional Chinese Medicine. 2017;33(09):56-8.
91. Jian Q, Weikang W, Junbiao L, Hanchuan L, Yayi Z, Zhiqiang L. Comparison Study On Coronary Heart Disease Patients With Angina Pectoris Singly Or Unitedly Treated With Sini Decoction And Isosorbide Dinitrate. Chinese Journal Of Traditional Medical Science And Technology. 2001(01):6-8+1.
92. Xin S. Observation On The Therapeutic Effect Of Modified Sini Decoction In Treating Angina Pectoris After Coronary Intervention. Cardiovascular Disease Electronic Journal Of Integrated Traditional Chinese And Western Medicine. 2015;3(35):31-2.
93. Yongfa W. A Comparative Study On The Treatment Of Coronary Heart Disease Angina Pectoris With The Use Of Qishui Decoction Alone Or In Combination With Xiaoxintong. Prevention And Treatment Of Cardiovascular Disease. 2017(10):48-50.
94. Chao X. The Clinical Effect Of Xiaochaihu Decoction In The Treatment Of Stagnation Of Qi Type Perimenopausal Angina Pectoris [Master's Degree]2021.
95. Xuexin Z, Yanjun L, Quan L. Clinical Observation Of Modified Xiaochaihu Decoction On Angina Pectoris After Percutaneous Coronary Intervention And Its Effect On Blood Lipid And Hemorheology. Hebei Journal Of Traditional Chinese Medicine. 2020;42(07):990-4.
96. Jun C, Feng L, Jingguo L, Yuelin S, Ying X. Clinical Observation On The Treatment Of Refractory Angina Pectoris After PCI With Additional Administration Of Modified Xiaoxianxiong Decoction. Journal Of Guangxi University Of Chinese Medicine. 2018;21(3):14-7.
97. Ailin L, Xiaoxia J, Lin M, Yongmei X. Clinical Study On The Treatment Of Refractory Angina Pectoris With Stagnation Of Qi And Blood Stasis Syndrome By Modified Xiaoxianxiong Decoction Combined With Conventional Western Medical Therapies. International Journal Of Traditional Chinese Medicine. 2019;41(9):941-5.
98. Jingjun L, Rui W, Jingyu X. Clinical Observation On The Treatment Of Unstable Angina Pectoris With Modified Xiaoxianxiong Decoction. Acta Chinese Medicine And Pharmacology. 2010;38(2):123-4.
99. Fang L. Clinical Research On Serum Concentration Of Resistin In Patients With Unstable Angina Pectoris Treated By Xiaoxianxiong Tang. Hebei Journal Of Traditional Chinese Medicine. 2014(8):1180-2,3.
100. Guoying L. Modified Xiaoxianxiong Decoction Combined With Conventional Western Medicine Treatment For 30 Cases Of Unstable Angina Pectoris. Traditional Chinese Medicinal Research. 2016;29(2):30-1.
101. Ruiwen X, Yingqiang G, Jiejun H. Clinical Study On Xiaoxianxiong Decoction For Treatment Of Refractory Angina Pectoris With Qi Stagnation And Blood Stasis Syndrome. Chinese Journal Of Information On Traditional Chinese Medicine. 2018;25(8):17-20.
102. Fang Z, Qingqiao Y, Jie W. Curative Efficacy Of Modified Xiaoxianxiong Decoction On Unstable Angina With Intermingled Phlegm-Heat Syndrome And Its Effects On Vascular Endothelial Function. Hebei Journal Of Traditional Chinese Medicine. 2017;39(9):1297-300.
103. Weichuang Z. Observation On The Therapeutic Effect Of Modified Xiaozhengchuan Decoction Combined With Conventional Western Medicine Therapy In The Treatment Of Angina Pectoris Caused By Qi Stagnation And Blood Stasis Syndrome. Heilongjiang Journal Of Traditional Chinese Medicine. 2021;50(4):106-7.
104. Mei D. Observation On Clinical Efficacy Of Zhigancao Decoction In Treating Unstable Angina Of Coronary Heart Disease. China's Rural Health. 2019(8):40.
105. Qingbing G. Observation On The Effect Of Zhi Gan Cao Decoction In Treating Unstable Angina Pectoris Of Coronary Heart Disease And Analysis Of Its Effectiveness Rate. Special Health. 2019(25):191-2.
106. Lidong N, Jiang H. Effect Of Zhigancao Decoction On Blood Lipid And Hemorheology In Patients With Unstable Angina Pectoris Of Coronary Heart Disease. China Modern Medicine. 2020;27(6):153-5,66.
107. Jin W, Xiaoyan Y, Xiaodong W. Clinical Observation On Zhigancao Decoction In Treatment Of Unstable Angina Pectoris Of Coronary Heart Disease. Journal Of Hubei University Of Chinese Medicine. 2017;19(6):67-9.
108. Xinyan Z. The Effect Of Traditional Chinese Medicine Was Observed. Advice For Health. 2023;17(7):184-6.
109. Jun D, Pengzhan X. Clinical Observation On Treating Angina Pectoris Of Coronary Heart Disease With Modified Zhishi Xiebai Guizhi Decoction. Inner Mongolia Journal Of Traditional Chinese. 2023;42(7):19-20.
110. Lina D, Xianxing H. A Randomized Controlled Study On The Effect Of Zhishi Xiebai Guizhi Decoction On Phlegm And Blood Stasis Obstruction Of Coronary Heart Disease. The Second National Medical Research Forum; 2023; Xi’an, Shaanxi, China.
111. Maiwen D. To Observe The Clinical Efficacy And Mechanism Of Action Of Zhishi Xiebai Guizhi Decoction In The Treatment Of Patients With Stable Angina Pectoris Due To Phlegm Obstructing The Heart Meridians. Oriental Medicated Diet. 2022(2):57-9.
112. Fei J. A Clinical Study On Treating Syndrome Of Phlegm Blocking Heart Vessel Of Stable Coronary Heart Disease With Zhishi Xiebai Guizhi Decoction Plus Western Medicine. Clinical Journal Of Chinese Medicine. 2021;13(2):11-5.
113. Tao LF, Dan LD. Clinical Study On Immature Bitter Orange And Chinese Chive And Cinnamon Twig Decoction In Treating Stable Coronary Heart Disease With Syndrome Of Phlegm Obstructing Heart Pulse. Henan Traditional Chinese Medicine. 2021;41(7):1003-6.
114. Yuchun L, Xiaomin T, Chaochao C, Jing W. Modified Zhishi Xiebai Guizhi Decoction As Adjuvant Treatment Of Stable Angina Pectoris And The Effect Of Vascular Endothelial Function. Clinical Journal Of Traditional Chinese Medicine. 2021;33(2):349-52.
115. Jianing L, Yaping Z. Clinical Study On Zhishi Xiebai Guizhi Decoction Combined With Western Medicine For Unstable Angina With Syndrome Of Qi Stagnation Due To Congealing Cold Combined Phlegm Stasis. New Chinese Medicine. 2023;55(20):36-9.
116. Shu L, Wei S, Weipeng W, Chungang Z, Shaoyu G, Feng G, Et Al. The Effect Of Zhishi Xiebai Guizhi Decoction On The MMP-9 Activity And Gene Expression In Patients With Unstable Angina Pectoris Of Phlegm-Stasis Cold Syndrome. Proceedings Of The 13th Academic Annual Conference Of The Chinese Society Of Traditional Chinese Medicine For Cardiac Diseases; 2011; Beijing, China.
117. Xunxun M. The Effect Of Huanglian Zhishi Xiebai Guizi Decoction On SOD And Hs-CRP In Patients With Pulmonary Obstruction Heart Disease And Coronary Heart Disease Syndrome [Master's Degree]2018.
118. Yueping S, Jun M. Clinical Observation Of Curative Effect On Modified Zhishi Xiebai Guizhi Decoction Treating Coronary Heart Disease Angina Pectoris. Liaoning Journal Of Traditional Chinese Medicine. 2009(6):962-3.
119. Xiaodong T. Clinical Evaluation Of Zhishi Xiebai Guizhi Decoction On Patients With Phlegm Obstructing Heart Pulse Syndrome Of Stable Coronary Heart Disease And Its Effect On Inflammatory Markers Of MLR, MHR And NLR [Doctoral Degree]2019.
120. Xiuhai W. Clinical Observation On 50 Cases Of Coronary Heart Disease Treated With Modified Zhishi Xiebai Guizhi Decoction. Chinese Journal Of Clinical Rational Drug Use. 2009;2(13):49.
121. Yuguang W, Yancai J. Clinical Observation On Treating 45 Cases Of UA With Zhishi Xiebai Guizhi Decoction Plus Modern Medicine. Clinical Journal Of Chinese Medicine. 2013;5(10):35-6.
122. Shukui W. Clinical Study On The Treatment Of Coronary Heart Disease Angina Pectoris With Addition And Subtraction Of Zhishi Xiebai Guizhi Decoction. Asia-Pacific Traditional Medicine. 2015;11(24):120-1.
123. Yijiao W. Clinical Observation Of Zhishi Xiebai Guizhi Decoction Modified In Angina After PCI Operation (Phlegm And Blood-Stasis Syndrome) [Master's Degree]2020.
124. Youshou Y. The Efficacy Of Zhishi Xiebai Guizhi Decoction Combined With Rosuvastatin In The Treatment Of Unstable Angina Pectoris And Its Effects On Blood Lipid And Hs-CRP Levels. Chinese Journal Of Clinical Rational Drug Use. 2022;15(29):36-9.
125. Ying YQ, Xiang G, Geng BH, Wang LZ, Xi-qian L, Lin L. Effects Of Zhishi Xiebai Guizhi Decoction On Serum Inflammatory Factors And Vascular Endothelial Function In Patients With Unstable Angina Pectoris. China Journal Of Traditional Chinese Medicine And Pharmacy. 2021;36(11):6893-7.
126. Lihui Z. Clinical Study Of Zhishi Xiebai Guizhi Decoction Added Flavor To Treat Coronary Heart Disease From Phlegm [Master's Degree]2024.
127. Tongwen Z, Boren Z. Clinical Observation On The Treatment Of Coronary Heart Disease With Modified Zhishi Xiebai Guizhi Decoction Combined With Western Medicine. Fujian Medical Journal. 2019;41(6):105-7.
128. Hongyuan Z. Clinical Study Of Zhishi Xiebai Guizhi Decoction In Treating Unstable Angina Pectoris Of Coronary Heart Disease [Master's Degree]2015.
129. Lianhong Z. Clinical Observation On The Modified Zhishi Xiebai Guizhi Decoction In The Treatment Of Coronary Heart Disease Angina Pectoris With Internal Obstruction Of Phlegm-Trouble Type. Journal Of Practical Traditional Chinese Medicine. 2023;39(11):2143-5.
130. Dejian Z. Effect Of Zhishi Xiebai Guizhi Decoction On FIB, D-D, Hcy And Hs-CRP In Patients With Unstable Angina Pectoris Due To Stagnation Of Cold, Phlegm, Blood Stasis And Qi Stagnation Syndrome [Master's Degree]2018.
131. Dejian Z, Shu L. Effect Of Zhishi Xiebai Guizhi Decoction On Hcy And Hs-CRP In Patients With Unstable Angina Pectoris. Journal Of Liaoning University Of Traditional Chinese Medicine. 2018;20(6):209-12.
