# Supplementary material for: Comparative efficacy of Traditional Chinese Medicine classical prescriptions for angina pectoris: a network meta-analysis
Source: Front Pharmacol. 2026 Sep 28;17:1794371. doi: 10.3389/fphar.2026.1794371 (PMC13654956; doi:10.3389/fphar.2026.1794371)
Supplement: Supplementary file 1 [file DataSheet1.ZIP › Supplementary material/data3_Table S4-S12_league table of sub-gruop.docx]

Table S4A The league table of Angina pectoris efficiency rate (SAP).

| Intervention | OR (95%CI) | | | | | | | |
| --- | --- | --- | --- | --- | --- | --- | --- | --- |
|  | Biomedicine | Biomedicine+ZSXBGZ | Biomedicine+XXX | Biomedicine+SN | Biomedicine+LGZG | Biomedicine+GZFL | Biomedicine+GLXBBX | Biomedicine+GLXBBJ |
| Biomedicine | 1 |  |  |  |  |  |  |  |
| Biomedicine+ZSXBGZ | 0.20 (0.09, 0.46) | 1 |  |  |  |  |  |  |
| Biomedicine+XXX | 0.57 (0.30, 1.07) | 2.83 (0.99, 8.08) | 1 |  |  |  |  |  |
| Biomedicine+SN | 0.41 (0.21, 0.82) | 2.08 (0.70, 6.11) | 0.73 (0.29, 1.88) | 1 |  |  |  |  |
| Biomedicine+LGZG | 0.20 (0.12, 0.36) | 1.02 (0.37, 2.80) | 0.36 (0.15, 0.85) | 0.49 (0.20, 1.20) | 1 |  |  |  |
| Biomedicine+GZFL | 0.28 (0.14, 0.57) | 1.40 (0.47, 4.22) | 0.50 (0.19, 1.28) | 0.68 (0.25, 1.80) | 1.38 (0.55, 3.42) | 1 |  |  |
| Biomedicine+GLXBBX | 0.31 (0.21, 0.46) | 1.57 (0.63, 3.94) | 0.55 (0.26, 1.16) | 0.76 (0.34, 1.65) | 1.54 (0.77, 3.03) | 1.12 (0.50, 2.48) | 1 |  |
| Biomedicine+GLXBBJ | 0.26 (0.11, 0.61) | 1.28 (0.38, 4.31) | 0.45 (0.15, 1.34) | 0.62 (0.20, 1.88) | 1.26 (0.44, 3.56) | 0.91 (0.30, 2.83) | 0.82 (0.31, 2.14) | 1 |

*OR < 1 indicates a favorable effect of the row intervention. A 95% CI crossing 1 suggests a non-significant difference (P > 0.05).

Table S4B The league table of Angina pectoris efficiency rate (UA).

| Intervention | OR (95%CI) | | | |
| --- | --- | --- | --- | --- |
|  | Biomdicine | Biomedicine+ZSXBGZ | Biomedicine+XXX | Biomedicine+GLXBBX |
| Biomedicine | 1 |  |  |  |
| Biomedicine+ZSXBGZ | 0.24 (0.12, 0.52) | 1 |  |  |
| Biomedicine+XXX | 0.38 (0.21, 0.70) | 1.55 (0.59, 4.10) | 1 |  |
| Biomedicine+GLXBBX | 0.24 (0.14, 0.42) | 0.98 (0.38, 2.51) | 0.63 (0.28, 1.43) | 1 |

*OR < 1 indicates a favorable effect of the row intervention. A 95% CI crossing 1 suggests a non-significant difference (P > 0.05).

Table S5A The league table of ECG efficiency rate (SAP).

| Intervention | OR (95%CI) | | | | | | |
| --- | --- | --- | --- | --- | --- | --- | --- |
|  | Biomedicine | Biomedicine+ZSXBGZ | Biomedicine+XXX | Biomedicine+SN | Biomedicine+LGZG | Biomedicine+GZFL | Biomedicine+GLXBBX |
| Biomedicine | 1 |  |  |  |  |  |  |
| Biomedicine+ZSXBGZ | 0.43 (0.28, 0.64) | 1 |  |  |  |  |  |
| Biomedicine+XXX | 0.57 (0.28, 1.15) | 1.32 (0.58, 3.00) | 1 |  |  |  |  |
| Biomedicine+SN | 0.57 (0.35, 0.90) | 1.31 (0.70, 2.46) | 0.99 (0.42, 2.34) | 1 |  |  |  |
| Biomedicine+LGZG | 0.30 (0.17, 0.52) | 0.70 (0.35, 1.38) | 0.53 (0.22, 1.30) | 0.53 (0.26, 1.09) | 1 |  |  |
| Biomedicine+GZFL | 0.66 (0.32, 1.38) | 1.55 (0.68, 3.60) | 1.17 (0.43, 3.25) | 1.19 (0.50, 2.80) | 2.23 (0.90, 5.53) | 1 |  |
| Biomedicine+GLXBBX | 0.33 (0.25, 0.44) | 0.77 (0.47, 1.28) | 0.58 (0.27, 1.26) | 0.59 (0.34, 1.03) | 1.11 (0.59, 2.05) | 0.50 (0.23, 1.09) | 1 |

*OR < 1 indicates a favorable effect of the row intervention. A 95% CI crossing 1 suggests a non-significant difference (P > 0.05).

Table S5B The league table of ECG efficiency rate (UA).

| Intervention | OR (95%CI) | | | |
| --- | --- | --- | --- | --- |
|  | Biomedicine | Biomedicine+ZSXBGZ | Biomedicine+GLXBBX | Biomedicine+DGSN |
| Biomedicine | 1 |  |  |  |
| Biomedicine+ZSXBGZ | 0.32 (0.17, 0.61) | 1 |  |  |
| Biomedicine+GLXBBX | 0.39 (0.23, 0.64) | 1.21 (0.53, 2.77) | 1 |  |
| Biomedicine+DGSN | 0.58 (0.28, 1.20) | 1.82 (0.69, 4.81) | 1.51 (0.62, 3.67) | 1 |

*OR < 1 indicates a favorable effect of the row intervention. A 95% CI crossing 1 suggests a non-significant difference (P > 0.05).

Table S6A The league table of TCM syndrome efficiency rate (SAP).

| Intervention | OR (95%CI) | | | | |
| --- | --- | --- | --- | --- | --- |
|  | Biomedicine | Biomedicine+ZSXBGZ | Biomedicine+GZFL | Biomedicine+GLXBBX | Biomedicine+DGSN |
| Biomedicine | 1 |  |  |  |  |
| Biomedicine+ZSXBGZ | 0.36 (0.21, 0.62) | 1 |  |  |  |
| Biomedicine+GZFL | 0.22 (0.12, 0.41) | 0.61 (0.27, 1.36) | 1 |  |  |
| Biomedicine+GLXBBX | 0.30 (0.21, 0.45) | 0.83 (0.43, 1.60) | 1.38 (0.66, 2.83) | 1 |  |
| Biomedicine+DGSN | 0.24 (0.10, 0.61) | 0.67 (0.23, 1.94) | 1.11 (0.36, 3.32) | 0.80 (0.30, 2.16) | 1 |

*OR < 1 indicates a favorable effect of the row intervention. A 95% CI crossing 1 suggests a non-significant difference (P > 0.05).

Table S6B The league table of TCM syndrome efficiency rate (UA).

| Intervention | OR (95%CI) | | | | | |
| --- | --- | --- | --- | --- | --- | --- |
|  | Biomedicine | Biomedicine+ZSXBGZ | Biomedicine+ZGC | Biomedicine+XXX | Biomedicine+GLXBBX | Biomedicine+DGSN |
| Biomedicine | 1 |  |  |  |  |  |
| Biomedicine+ZSXBGZ | 0.38 (0.21, 0.68) | 1 |  |  |  |  |
| Biomedicine+ZGC | 0.23 (0.09, 0.55) | 0.59 (0.20, 1.72) | 1 |  |  |  |
| Biomedicine+XXX | 0.28 (0.13, 0.61) | 0.74 (0.28, 1.95) | 1.25 (0.38, 4.10) | 1 |  |  |
| Biomedicine+GLXBBX | 0.38 (0.22, 0.65) | 0.99 (0.44, 2.20) | 1.67 (0.58, 4.76) | 1.34 (0.52, 3.46) | 1 |  |
| Biomedicine+DGSN | 0.25 (0.10, 0.59) | 0.65 (0.23, 1.86) | 1.09 (0.32, 3.82) | 0.88 (0.28, 2.83) | 0.66 (0.24, 1.84) | 1 |

*OR < 1 indicates a favorable effect of the row intervention. A 95% CI crossing 1 suggests a non-significant difference (P > 0.05).

Table 7A The league table of and LDL-C level (SAP).

| Intervention | SMD (95%CI) | | | | | | | | |
| --- | --- | --- | --- | --- | --- | --- | --- | --- | --- |
|  | Biomedicine | Biomedicine+ZSXBGZ | Biomedicine+XCH | Biomedicine+RS | Biomedicine+LGZG | Biomedicine+GZFL | Biomedicine+GLXBBX | Biomedicine+GLXBBJ | Biomedicine+DGSN |
| Biomedicine | 0 |  |  |  |  |  |  |  |  |
| Biomedicine+ZSXBGZ | -3.91 (-8.84, 1.02) | 0 |  |  |  |  |  |  |  |
| Biomedicine+XCH | -6.77 (-15.30, 1.75) | -2.86 (-12.71, 6.99) | 0 |  |  |  |  |  |  |
| Biomedicine+RS | -25.35 (-34.73, -15.98) | -21.44 (-32.03, -10.85) | -18.58 (-31.25, -5.90) | 0 |  |  |  |  |  |
| Biomedicine+LGZG | -4.99 (-10.38, 0.39) | -1.08 (-8.39, 6.22) | 1.78 (-8.31, 11.87) | 20.36 (9.55, 31.17) | 0 |  |  |  |  |
| Biomedicine+GZFL | -9.87 (-15.97, -3.77) | -5.96 (-13.80, 1.89) | -3.10 (-13.58, 7.39) | 15.48 (4.33, 26.64) | -4.88 (-13.02, 3.27) | 0 |  |  |  |
| Biomedicine+GLXBBX | -2.46 (-5.38, 0.46) | 1.45 (-4.27, 7.18) | 4.32 (-4.70, 13.33) | 22.90 (13.08, 32.71) | 2.54 (-3.59, 8.67) | 7.41 (0.65, 14.18) | 0 |  |  |
| Biomedicine+GLXBBJ | -4.87 (-10.89, 1.15) | -0.96 (-8.74, 6.82) | 1.91 (-8.53, 12.34) | 20.48 (9.34, 31.62) | 0.12 (-7.96, 8.20) | 5.00 (-3.57, 13.57) | -2.41 (-9.10, 4.28) | 0 |  |
| Biomedicine+DGSN | -4.38 (-11.34, 2.58) | -0.47 (-9.00, 8.06) | 2.39 (-8.62, 13.40) | 20.97 (9.29, 32.65) | 0.61 (-8.19, 9.42) | 5.49 (-3.77, 14.75) | -1.92 (-9.48, 5.63) | 0.49 (-8.72, 9.69) | 0 |

*SMD < 0 indicates a favorable effect of the row intervention. A 95% CI crossing 0 suggests a non-significant difference (P > 0.05).

Table 7B The league table of and LDL-C level (UA).

| Intervention | SMD (95%CI) | | | | |
| --- | --- | --- | --- | --- | --- |
|  | Biomedicine | Biomedicine+ZSXBGZ | Biomedicine+ZGC | Biomedicine+RS | Biomedicine+GLXBBX |
| Biomedicine | 0 |  |  |  |  |
| Biomedicine+ZSXBGZ | -5.11 (-11.05,0.84) | 0 |  |  |  |
| Biomedicine+ZGC | -6.42 (-11.29,-1.55) | -1.31 (-9.00,6.37) | 0 |  |  |
| Biomedicine+RS | -1.08 (-5.91,3.74) | 4.02 (-3.63,11.68) | 5.34 (-1.52,12.19) | 0 |  |
| Biomedicine+GLXBBX | -8.47 (-14.51,-2.44) | -3.37 (-11.83,5.10) | -2.05 (-9.80,5.70) | -7.39 (-15.11,0.34) | 0 |

*SMD < 0 indicates a favorable effect of the row intervention. A 95% CI crossing 0 suggests a non-significant difference (P > 0.05).

Table S8 The league table of HDL-C level (SAP).

| Intervention | SMD (95%CI) | | | | | | | |
| --- | --- | --- | --- | --- | --- | --- | --- | --- |
|  | Biomedicine | Biomedicine+ZSXBGZ | Biomedicine+RS | Biomedicine+LGZG | Biomedicine+GZFL | Biomedicine+GLXBBX | Biomedicine+GLXBBJ | Biomedicine+DGSN |
| Biomedicine | 0 |  |  |  |  |  |  |  |
| Biomedicine+ZSXBGZ | -2.77 (-8.06, 2.52) | 0 |  |  |  |  |  |  |
| Biomedicine+RS | -4.22 (-12.58, 4.14) | -1.45 (-11.34, 8.44) | 0 |  |  |  |  |  |
| Biomedicine+LGZG | -1.82 (-6.64, 3.00) | 0.95 (-6.21, 8.11) | 2.40 (-7.25, 12.05) | 0 |  |  |  |  |
| Biomedicine+GZFL | -1.58 (-7.48, 4.32) | 1.19 (-6.73, 9.12) | 2.65 (-7.59, 12.88) | 0.24 (-7.37, 7.86) | 0 |  |  |  |
| Biomedicine+GLXBBX | -1.51 (-4.46, 1.45) | 1.27 (-4.79, 7.33) | 2.72 (-6.15, 11.58) | 0.31 (-5.34, 5.97) | 0.07 (-6.53, 6.67) | 0 |  |  |
| Biomedicine+GLXBBJ | -10.92 (-17.14, -4.71) | -8.15 (-16.31, 0.01) | -6.70 (-17.12, 3.72) | -9.10 (-16.97, -1.24) | -9.34 (-17.91, -0.77) | -9.41 (-16.30, -2.53) | 0 |  |
| Biomedicine+DGSN | -2.28 (-10.63, 6.08) | 0.50 (-9.39, 10.39) | 1.95 (-9.87, 13.77) | -0.45 (-10.10, 9.20) | -0.70 (-10.93, 9.53) | -0.77 (-9.63, 8.10) | 8.65 (-1.77, 19.06) | 0 |

*SMD < 0 indicates a favorable effect of the row intervention. A 95% CI crossing 0 suggests a non-significant difference (P > 0.05).

Table S9A The league table of TG level (SAP).

| Intervention | SMD (95%CI) | | | | | | | | |
| --- | --- | --- | --- | --- | --- | --- | --- | --- | --- |
|  | Biomedicine | Biomedicine+ZSXBGZ | Biomedicine+XCH | Biomedicine+RS | Biomedicine+LGZG | Biomedicine+GZFL | Biomedicine+GLXBBX | Biomedicine+GLXBBJ | Biomedicine+DGSN |
| Biomedicine | 0 |  |  |  |  |  |  |  |  |
| Biomedicine+ZSXBGZ | -2.93 (-7.11, 1.25) | 0 |  |  |  |  |  |  |  |
| Biomedicine+XCH | -3.23 (-11.56, 5.11) | -0.29 (-9.62, 9.03) | 0 |  |  |  |  |  |  |
| Biomedicine+RS | -7.85 (-16.24, 0.54) | -4.92 (-14.29, 4.45) | -4.62 (-16.45, 7.20) | 0 |  |  |  |  |  |
| Biomedicine+LGZG | -3.50 (-8.77, 1.77) | -0.57 (-7.29, 6.15) | -0.27 (-10.13, 9.58) | 4.35 (-5.56, 14.25) | 0 |  |  |  |  |
| Biomedicine+GZFL | -13.81 (-19.78, -7.83) | -10.88 (-18.16, -3.59) | -10.58 (-20.83, -0.33) | -5.96 (-16.26, 4.34) | -10.31 (-18.27, -2.34) | 0 |  |  |  |
| Biomedicine+GLXBBX | -2.22 (-5.00, 0.56) | 0.71 (-4.31, 5.72) | 1.00 (-7.78, 9.79) | 5.62 (-3.21, 14.46) | 1.28 (-4.68, 7.23) | 11.58 (4.99, 18.17) | 0 |  |  |
| Biomedicine+GLXBBJ | -6.65 (-12.56, -0.75) | -3.72 (-10.95, 3.51) | -3.42 (-13.64, 6.79) | 1.20 (-9.06, 11.46) | -3.15 (-11.06, 4.76) | 7.16 (-1.24, 15.56) | -4.43 (-10.95, 2.10) | 0 |  |
| Biomedicine+DGSN | -3.77 (-10.59, 3.05) | -0.84 (-8.84, 7.16) | -0.54 (-11.31, 10.23) | 4.08 (-6.73, 14.89) | -0.27 (-8.89, 8.35) | 10.04 (0.97, 19.11) | -1.54 (-8.91, 5.82) | 2.88 (-6.14, 11.91) | 0 |

*SMD < 0 indicates a favorable effect of the row intervention. A 95% CI crossing 0 suggests a non-significant difference (P > 0.05).

Table S9B The league table of TG level (UA).

| Intervention | SMD (95%CI) | | | | | | |
| --- | --- | --- | --- | --- | --- | --- | --- |
|  | Biomedicine | Biomedicine+ZGC | Biomedicine+RS | Biomedicine+LGZG | Biomedicine+GZFL | Biomedicine+GLXBBX | Biomedicine+GLXBBJ |
| Biomedicine | 0 |  |  |  |  |  |  |
| Biomedicine+ZGC | -4.66 (-6.52, -2.80) | 0 |  |  |  |  |  |
| Biomedicine+RS | -1.18 (-2.61, 0.26) | 3.48 (1.14, 5.83) | 0 |  |  |  |  |
| Biomedicine+LGZG | -2.15 (-4.66, 0.35) | 2.51 (-0.61, 5.62) | -0.98 (-3.86, 1.90) | 0 |  |  |  |
| Biomedicine+GZFL | -0.57 (-3.04, 1.89) | 4.09 (1.00, 7.17) | 0.60 (-2.25, 3.45) | 1.58 (-1.93, 5.09) | 0 |  |  |
| Biomedicine+GLXBBX | -1.48 (-2.72, -0.24) | 3.18 (0.95, 5.41) | -0.30 (-2.20, 1.59) | 0.68 (-2.12, 3.47) | -0.91 (-3.66, 1.85) | 0 |  |
| Biomedicine+GLXBBJ | -43.89 (-50.89, -36.90) | -39.23 (-46.47, -32.00) | -42.72 (-49.86, -35.58) | -41.74 (-49.17, -34.31) | -43.32 (-50.73, -35.90) | -42.41 (-49.52, -35.31) | 0 |

*SMD < 0 indicates a favorable effect of the row intervention. A 95% CI crossing 0 suggests a non-significant difference (P > 0.05).

Table S10A The league table of TC level (SAP).

| Intervention | SMD (95%CI) | | | | | | | | |
| --- | --- | --- | --- | --- | --- | --- | --- | --- | --- |
|  | Biomedicine | Biomedicine+ZSXBGZ | Biomedicine+XCH | Biomedicine+RS | Biomedicine+LGZG | Biomedicine+GZFL | Biomedicine+GLXBBX | Biomedicine+GLXBBJ | Biomedicine+DGSN |
| Biomedicine | 0 |  |  |  |  |  |  |  |  |
| Biomedicine+ZSXBGZ | -5.13 (-8.48, -1.77) | 0 |  |  |  |  |  |  |  |
| Biomedicine+XCH | -3.44 (-10.04, 3.15) | 1.68 (-5.72, 9.08) | 0 |  |  |  |  |  |  |
| Biomedicine+RS | -9.86 (-16.57, -3.14) | -4.73 (-12.24, 2.78) | -6.41 (-15.83, 3.00) | 0 |  |  |  |  |  |
| Biomedicine+LGZG | -6.11 (-10.30, -1.92) | -0.98 (-6.35, 4.38) | -2.66 (-10.48, 5.15) | 3.75 (-4.17, 11.66) | 0 |  |  |  |  |
| Biomedicine+GZFL | -19.09 (-24.64, -13.54) | -13.96 (-20.44, -7.48) | -15.65 (-24.26, -7.03) | -9.23 (-17.95, -0.52) | -12.98 (-19.93, -6.03) | 0 |  |  |  |
| Biomedicine+GLXBBX | -2.14 (-4.22, -0.05) | 2.99 (-0.96, 6.94) | 1.31 (-5.61, 8.22) | 7.72 (0.68, 14.75) | 3.97 (-0.71, 8.65) | 16.95 (11.02, 22.88) | 0 |  |  |
| Biomedicine+GLXBBJ | -5.92 (-10.60, -1.24) | -0.79 (-6.55, 4.96) | -2.47 (-10.56, 5.61) | 3.94 (-4.25, 12.12) | 0.19 (-6.09, 6.47) | 13.17 (5.92, 20.43) | -3.78 (-8.90, 1.34) | 0 |  |
| Biomedicine+DGSN | -1.58 (-6.96, 3.81) | 3.55 (-2.79, 9.89) | 1.87 (-6.64, 10.38) | 8.28 (-0.33, 16.89) | 4.53 (-2.29, 11.35) | 17.51 (9.78, 25.24) | 0.56 (-5.21, 6.33) | 4.34 (-2.79, 11.47) | 0 |

*SMD < 0 indicates a favorable effect of the row intervention. A 95% CI crossing 0 suggests a non-significant difference (P > 0.05).

Table S10B The league table of TC level (UA).

| Intervention | SMD (95%CI) | | | | | |
| --- | --- | --- | --- | --- | --- | --- |
|  | Biomedicine | Biomedicine+ZGC | Biomedicine+RS | Biomedicine+LGZG | Biomedicine+GZFL | Biomedicine+GLXBBX |
| Biomedicine | 0 |  |  |  |  |  |
| Biomedicine+ZGC | -20.69 (-30.13, -11.25) | 0 |  |  |  |  |
| Biomedicine+RS | -1.46 (-10.54, 7.62) | 19.23 (6.13, 32.33) | 0 |  |  |  |
| Biomedicine+LGZG | -3.31 (-14.43, 7.82) | 17.38 (2.79, 31.97) | -1.85 (-16.21, 12.51) | 0 |  |  |
| Biomedicine+GZFL | -0.65 (-11.77, 10.46) | 20.03 (5.45, 34.62) | 0.80 (-13.55, 15.16) | 2.65 (-13.07, 18.38) | 0 |  |
| Biomedicine+GLXBBX | -2.05 (-13.18, 9.07) | 18.63 (4.05, 33.22) | -0.59 (-14.95, 13.77) | 1.26 (-14.48, 16.99) | -1.40 (-17.13, 14.33) | 0 |

*SMD < 0 indicates a favorable effect of the row intervention. A 95% CI crossing 0 suggests a non-significant difference (P > 0.05).

Table S11 The league table of Hs-CRP level (SAP).

| Intervention | SMD (95%) | | |
| --- | --- | --- | --- |
|  | Biomedicine | Biomedicine+ZSXBGZ | Biomedicine+GLXBBX |
| Biomedicine | 0 |  |  |
| Biomedicine+ZSXBGZ | -3.54 (-5.37, -1.71) | 0 |  |
| Biomedicine+GLXBBX | -1.60 (-3.59, 0.39) | 1.94 (-0.76, 4.64) | 0 |

*SMD < 0 indicates a favorable effect of the row intervention. A 95% CI crossing 0 suggests a non-significant difference (P > 0.05).

Table S12A The league table of adverse effect (SAP).

| Intervention | OR (95%) | | | | |
| --- | --- | --- | --- | --- | --- |
|  | Biomedicine | Biomedicine+ZSXBGZ | Biomedicine+LGZG | Biomedicine+GZFL | Biomedicine+GLXBBX |
| Biomedicine | 1 |  |  |  |  |
| Biomedicine+ZSXBGZ | 1.19 (0.55, 2.56) | 1 |  |  |  |
| Biomedicine+LGZG | 2.23 (0.84, 5.87) | 1.86 (0.55, 6.36) | 1 |  |  |
| Biomedicine+GZFL | 2.41 (1.22, 4.81) | 2.03 (0.73, 5.64) | 1.09 (0.33, 3.56) | 1 |  |
| Biomedicine+GLXBBX | 1.51 (0.84, 2.69) | 1.26 (0.48, 3.32) | 0.68 (0.22, 2.10) | 0.62 (0.25, 1.54) | 1 |

*OR < 1 indicates that the intervention in the row is associated with a higher rate of adverse reactions compared with the intervention in the column. A 95% CI crossing 1 suggests a non-significant difference (P > 0.05).

Table S12B The league table of adverse effect (UA).

| Intervention | OR (95%) | | | | |
| --- | --- | --- | --- | --- | --- |
|  | Biomedicine | Biomedicine+ZSXBGZ | Biomedicine+XXX | Biomedicine+RS | Biomedicine+GLXBBX |
| Biomedicine | 1 |  |  |  |  |
| Biomedicine+ZSXBGZ | 1.48 (0.94, 2.34) | 1 |  |  |  |
| Biomedicine+XXX | 1.28 (0.32, 5.10) | 0.86 (0.20, 3.71) | 1 |  |  |
| Biomedicine+RS | 1.35 (0.29, 6.30) | 0.91 (0.18, 4.57) | 1.05 (0.13, 8.42) | 1 |  |
| Biomedicine+GLXBBX | 0.40 (0.04, 4.48) | 0.27 (0.02, 3.16) | 0.31 (0.02, 5.05) | 0.30 (0.02, 5.21) | 1 |

*OR < 1 indicates that the intervention in the row is associated with a higher rate of adverse reactions compared with the intervention in the column. A 95% CI crossing 1 suggests a non-significant difference (P > 0.05).
