# Supplementary material for: Comparative efficacy of Traditional Chinese Medicine classical prescriptions for angina pectoris: a network meta-analysis
Source: Front Pharmacol. 2026 Sep 28;17:1794371. doi: 10.3389/fphar.2026.1794371 (PMC13654956; doi:10.3389/fphar.2026.1794371)
Supplement: Supplementary file 1 [file DataSheet1.ZIP › Supplementary material/data4_Table S13 & Figure S6-23_heterogeneity assessment.docx]

Table S13 Results of heterogeneity assessment.

| Outcomes | I^2^ (%) | | |
| --- | --- | --- | --- |
|  | Total | Sub-group-SAP | Sub-group-UA |
| Angina pectoris efficiency rate | 0 | 0 | 0 |
| ECG efficiency rate | 0 | 2 | 3 |
| TCM syndromes efficiency rate | 0 | 0 | 0 |
| LDL-C level | 98 | 98 | 98 |
| HDL-C levels | 97 | 97 | — |
| TG levels | 98 | 98 | 97 |
| TC levels | 98 | 98 | 97 |
| Hs-CRP levels | 97 | 97 | — |
| Adverse effect | 25 | 29 | 12 |

Figure S6 Forest plot of Angina pectoris efficiency rate (Total).

Figure S7 Forest plot of ECG efficiency rate (Total).

Figure S8 Forest plot of TCM syndromes efficiency rate (Total).

Figure S9 Forest plot of LDL-C level (Total).

Figure S10 Forest plot of HDL-C levels (Total).

Figure S11 Forest plot of TG levels (Total).

Figure S12 Forest plot of TC levels (Total).

Figure S13 Forest plot of Hs-CRP levels (Total).

Figure S14 Forest plot of Adverse effect (Total).

Figure S15 Forest plot of Angina pectoris efficiency rate (Sub-group).

Figure S23 Forest plot of Adverse effect (Sub-group).

Figure S16 Forest plot of ECG efficiency rate (Sub-group).

Figure S17 Forest plot of TCM syndromes efficiency rate (Sub-group).

Figure S18 Forest plot of LDL-C level (Sub-group).

Figure S19 Forest plot of HDL-C levels (Sub-group).

Figure S20 Forest plot of TG levels (Sub-group).

Figure S21 Forest plot of TC levels (Sub-group).

Figure S22 Forest plot of Hs-CRP levels (Sub-group).

Figure S23 Forest plot of adverse effects (Sub-group).
