# Supplementary material for: Comparative efficacy of Traditional Chinese Medicine classical prescriptions for angina pectoris: a network meta-analysis
Source: Front Pharmacol. 2026 Sep 28;17:1794371. doi: 10.3389/fphar.2026.1794371 (PMC13654956; doi:10.3389/fphar.2026.1794371)
Supplement: Supplementary file 1 [file DataSheet1.ZIP › Supplementary material/data5_Table S14_details of adverse effect.docx]

**Table S14** Reported adverse reactions.

| Study ID | Intervention | Adverse effects |
| --- | --- | --- |
| JIA XY 2020 | Biomedicine+DGSN | None |
| SUN XH 2022 | Biomedicine+DGSN | 1 case of MACE |
| YUAN QM 2020 | Biomedicine+DGSN | None |
| HU J 2018 | Biomedicine+GLXBBJ | 1 case of facial flushing, 1 case of nausea |
| GUO CJ 2024 | Biomedicine+GLXBBX | 3 cases of gastrointestinal symptoms, 1 case of headache |
| HUANG PP 2020 | Biomedicine+GLXBBX | None |
| LONG C 2019 | Biomedicine+GLXBBX | 1 case of diarrhea |
| MENG M 2012 | Biomedicine+GLXBBX | None |
| MI XS 2011 | Biomedicine+GLXBBX | None |
| NIU YW 2022 | Biomedicine+GLXBBX | 1 case of dizziness, 2 cases of gastrointestinal symptoms |
| TIAN DF 2016 | Biomedicine+GLXBBX | None |
| WANG P 2021 | Biomedicine+GLXBBX | 1 case of decreased appetite |
| WEI M 2009 | Biomedicine+GLXBBX | 2 cases of gastrointestinal reaction |
| YANG JK 2015 | Biomedicine+GLXBBX | None |
| YANG XY 2007 | Biomedicine+GLXBBX | None |
| YANG X 2023 | Biomedicine+GLXBBX | None |
| ZHANG ZF 2016 | Biomedicine+GLXBBX | 1 case of nausea |
| ZHAO YL 2013 | Biomedicine+GLXBBX | None |
| ZHAO YG 2024 | Biomedicine+GLXBBX | 3 cases of gastrointestinal reaction, 1 case of nausea, 1 case of vomiting, 3 cases of dizziness |
| ZHENG XB 2020 | Biomedicine+GLXBBX | 1 case of oral ulcer, 1 case of diarrhea |
| HUANG PY 2008 | Biomedicine+GZFL | None |
| LU XY 2012 | Biomedicine+GZFL | 8 cases of dizziness, distending headache, pulsating sensation in head, facial flushing, palpitation and blood pressure drop |
| PEI Q 2013 | Biomedicine+GZFL | None |
| XV Y 2023 | Biomedicine+GZFL | None |
| ZHENG WD 2015 | Biomedicine+GZFL | None |
| ZHONG F 2022 | Biomedicine+GZFL | 2 cases of arrhythmia, 1 case of stroke, 1 case of cardiac death |
| ZHOU WR 2017 | Biomedicine+GZFL | 1 case of premature ventricular contraction, 2 cases of nausea and vomiting, 2 cases of hypotension |
| AN LJ 2017 | Biomedicine+LGZG | None |
| HUA MZ 2023 | Biomedicine+LGZG | 2 cases of dizziness |
| JIANG XQ 2019 | Biomedicine+LGZG | 2 cases of headache |
| YU CL 2020 | Biomedicine+LGZG | 2 cases of nausea and vomiting, 1 case of leukopenia, 2 cases of liver dysfunction |
| FEI Y 2015 | Biomedicine+RS | None |
| WANG YW 2021 | Biomedicine+RS | None |
| WEN YY 2023 | Biomedicine+RS | 1 case of gastrointestinal reaction |
| SONG X 2015 | Biomedicine+SN | None |
| XV C 2021 | Biomedicine+XCH | None |
| LI JJ 2010 | Biomedicine+XXX | None |
| LIU F 2014 | Biomedicine+XXX | None |
| ZHOU F 2017 | Biomedicine+XXX | 1 case of stomach pain, 1 case of abdominal distension |
| DOU LN 2023 | Biomedicine+ZSXBGZ | 2 cases of gastrointestinal reaction, 1 case of dizziness and palpitation |
| JIA F 2021 | Biomedicine+ZSXBGZ | 2 cases of abdominal pain and diarrhea, 1 case of fever and fatigue |
| LI YC 2021 | Biomedicine+ZSXBGZ | None |
| LIU JN 2023 | Biomedicine+ZSXBGZ | 7 cases of headache and dizziness, 12 cases of gastrointestinal symptoms, 3 cases of spontaneous bleeding, 2 cases of allergic reaction |
| MA XX 2018 | Biomedicine+ZSXBGZ | None |
| TAN XD 2019 | Biomedicine+ZSXBGZ | 5 cases of abdominal pain and diarrhea, 2 cases of fever and fatigue |
| WANG YG 2013 | Biomedicine+ZSXBGZ | None |
| WU YJ 2020 | Biomedicine+ZSXBGZ | None |
| YUAN QY 2021 | Biomedicine+ZSXBGZ | 2 cases of liver dysfunction, 6 cases of gastrointestinal reaction, 2 cases of rash, 3 cases of dizziness, 2 cases of palpitation |
| ZHANG LH 2024 | Biomedicine+ZSXBGZ | 3 cases of abdominal distension |
| ZHOU HY 2015 | Biomedicine+ZSXBGZ | None |
| ZHOU LH 2023 | Biomedicine+ZSXBGZ | 1 case of headache and dizziness, 2 cases of gastrointestinal reaction |
| ZHU DJ 2018-1 | Biomedicine+ZSXBGZ | None |
