# Supplementary material for: Comparative efficacy of Traditional Chinese Medicine classical prescriptions for angina pectoris: a network meta-analysis
Source: Front Pharmacol. 2026 Sep 28;17:1794371. doi: 10.3389/fphar.2026.1794371 (PMC13654956; doi:10.3389/fphar.2026.1794371)
Supplement: Supplementary file 1 [file DataSheet1.ZIP › Supplementary material/data7_Figure S2-S5_figure of sub-gruop.docx]

Figure S2 Network evidence plot (SAP).

(A). Angina pectoris efficiency rate.

(B). ECG efficiency rate.

(C). TCM syndrome efficiency rate.

(D). LDL-C.

(E). HDL-C.

(F). TG.

(G). TC.

(H). Hs-CRP.

(I ). Adverse effect.

Figure S3 Network evidence plot (UA).

(A). Angina pectoris efficiency rate.

(B). ECG efficiency rate.

(C). TCM syndrome efficiency rate.

(D). LDL-C.

(E). TG.

(F). TC.

(G). Adverse effect.

Figure S4 Results from SUCRA (SAP). (SUCRA results are presented as exploratory, probability-based summaries in an open network meta-analysis framework.)

(A). Angina pectoris efficiency rate.

(B). ECG efficiency rate.

(C). TCM syndrome efficiency rate.

(D). LDL-C.

(E). HDL-C.

(F). TG.

(G). TC.

(H). Hs-CRP.

(I ). Adverse effect.

Figure S5 Results from SUCRA (UA). (SUCRA results are presented as exploratory, probability-based summaries in an open network meta-analysis framework.)

(A). Angina pectoris efficiency rate.

(B). ECG efficiency rate.

(C). TCM syndrome efficiency rate.

(D). LDL-C.

(E). TG.

(F). TC.

(G). Adverse effect.
