# Supplementary material for: Comparative efficacy of Traditional Chinese Medicine classical prescriptions for angina pectoris: a network meta-analysis
Source: Front Pharmacol. 2026 Sep 28;17:1794371. doi: 10.3389/fphar.2026.1794371 (PMC13654956; doi:10.3389/fphar.2026.1794371)
Supplement: Supplementary file 1 [file DataSheet1.ZIP › Supplementary material/data8_Figure S24-S28_sensitive analysis.docx]

**sSensitivity Analysis**

Figure S24. Sensitive analysis of LDL-C .

Figure S25. Sensitive analysis of HDL-C .

Figure S26. Sensitive analysis of TG .

Figure S27. Sensitive analysis of TC .

Figure S28. Sensitive analysis of Hs-CRP .
