# Supplementary material for: Comparative efficacy of Traditional Chinese Medicine classical prescriptions for angina pectoris: a network meta-analysis
Source: Front Pharmacol. 2026 Sep 28;17:1794371. doi: 10.3389/fphar.2026.1794371 (PMC13654956; doi:10.3389/fphar.2026.1794371)
Supplement: Supplementary file 1 [file DataSheet1.ZIP › Supplementary material/data9_Evaluation criteria for the effectiveness of TCM syndromes.docx]

**Evaluation criteria for the effectiveness of TCM syndromes**

Treatment response is categorized into four levels according to the percentage reduction in syndrome score:

①Markedly effective: ≥70% reduction in syndrome score, with significant improvement or resolution of clinical symptoms and signs;

②Effective: 30%–70% reduction in syndrome score, with improvement in clinical symptoms and signs;

③Ineffective: <30% reduction in syndrome score, with no obvious improvement in symptoms and signs;

④Worsened: <0% reduction in syndrome score, indicating aggravation of clinical symptoms and signs.

Evaluation scale example (deficiency cold syndrome)：

From [1]Chen Jialin. Effect of Ginseng Decoction on atherogenic index of plasma in patients with unstable angina pectoris of Xu Han type. [D]. Master’s thesis, Nanjing University of Chinese Medicine, 2017.
